# Dihapto-Coordinated Conjugated Carbocycles (η^2^‑C_*n*_H_*n*_ *n* = 5–8): Blurring the Line Between Aromatic and Antiaromatic Hydrocarbons

**DOI:** 10.1021/jacs.5c09111

**Published:** 2025-07-24

**Authors:** Megan N. Ericson, Josh K. Heman-Ackah, Rachel F. Lombardo, Alvin Q. Meng, Mason R. Ortiz, Sofia E. Megert, Diane A. Dickie, W. Dean Harman

**Affiliations:** Department of Chemistry, University of Virginia, Charlottesville, Virginia 22904, United States

## Abstract

The tungsten fragment {WTp­(NO)­(PMe_3_)} (Tp = trispyrazolylborate) is an effective dearomatization agent for benzene and its derivatives. The dihapto-coordination of this system to an arene disrupts its aromatic stability, thereby promoting facile electrophilic additions to the hydrocarbon, which can then be followed by the addition of nucleophiles. This preliminary study endeavors to extend this conceptual approach to other aromatic and antiaromatic carbocycles. Dihapto-coordinated complexes of η^2^-tropylium, η^2^-cyclopentadienyl cation, and η^2^-cyclooctatetraene have been synthesized and characterized using SC-XRD, DFT, CV, and ^1^H, ^31^P, and ^13^C NMR (including COSY, NOESY, HSQC, HMBC). Their fluxional behavior and reactivity toward electrophilic/nucleophilic additions, such as protonation and methylation, are also demonstrated.

## Graphical abstract

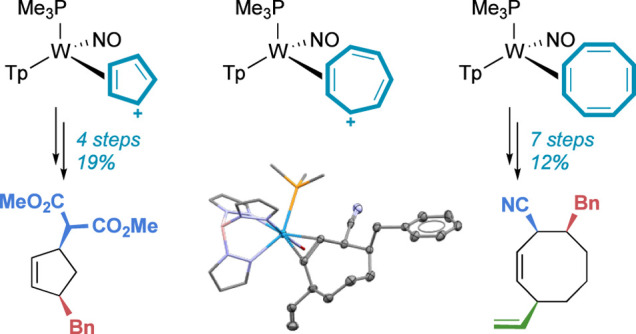

## Introduction

Over the past two decades, the π-base {WTp­(NO)­(PMe_3_)} has been shown to have the unusual ability to dearomatize benzenes through the coordination of two carbons (η^2^).^1,2^ Such binding renders the uncoordinated portion of the arene similar to a conjugated diene, both structurally and chemically. This feature has been used to actualize a wide variety of chemical transformations that are complementary to other dearomatization strategies.^3–8^ Through strong π-donation, the tungsten complex activates the benzene toward the addition of electrophiles and subsequently stabilizes the resulting arenium intermediates, thereby enabling the addition of nucleophiles. The resulting η^2^-diene complexes can be treated sequentially with a second electrophile and nucleophile to provide 1,4-disubstituted cyclohexenes.^9–11^ Owing to the steric bulk of the tungsten complex, electrophiles and nucleophiles stereoselectively add anti to the metal. Therefore, when an enantioenriched form of {WTp­(NO)­(PMe_3_)} is used,^12^ organic compounds can be prepared in high enantiomeric excess.^9,10^ We queried whether a similar process could be developed, starting from other aromatic and antiaromatic hydrocarbons ([C_*n*_H_*n*_]^*m*+^, where *m* = 0 for *n* = 6 or 8, and *m* = 1 for *n* = 5 or 7), with an overall goal of generating additional ring sizes of highly functionalized cycloalkenes (Figure 1).

**Figure 1:**
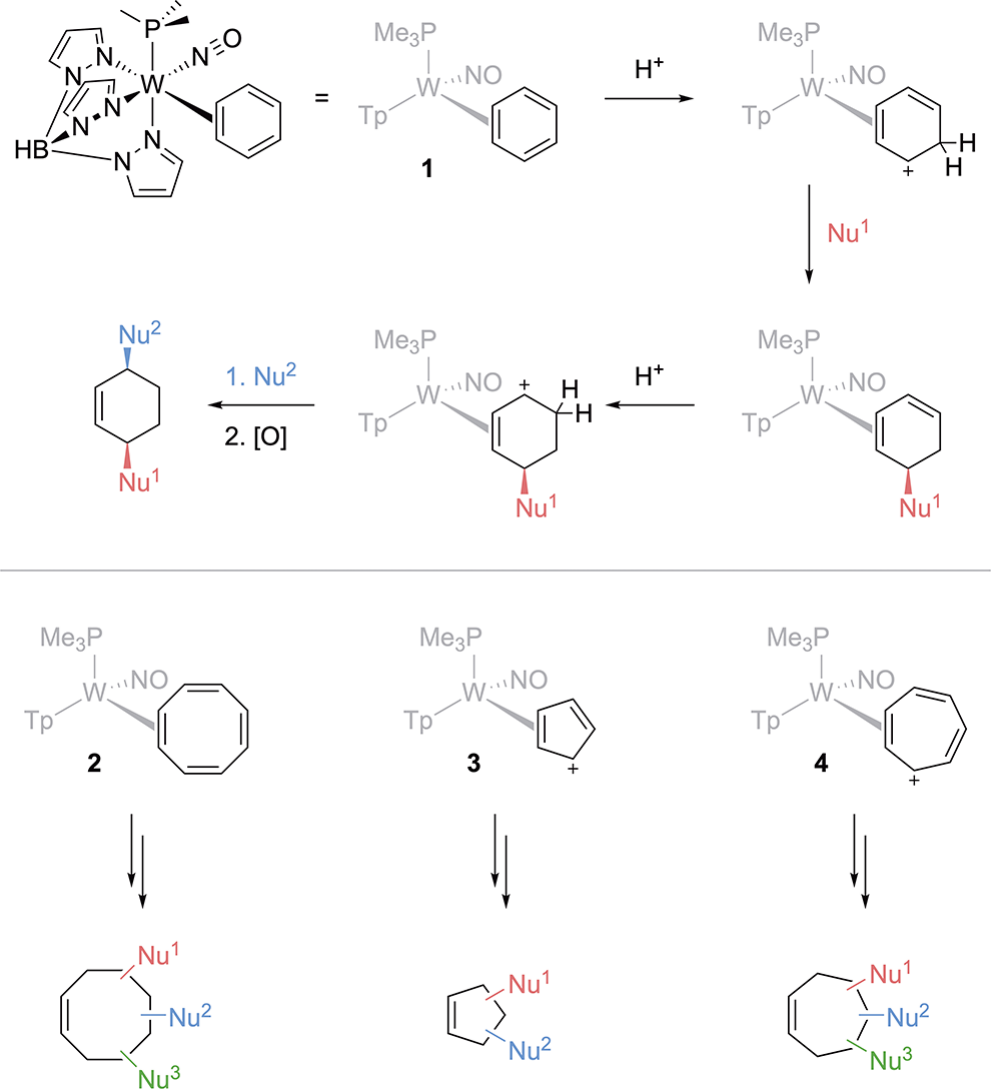
Elaboration of an η^2^-C_6_H_6_ complex (**1**) into difunctionalized cyclohexenes and the potential parallel reactions of η^2^-C_8_H_8_ (**2**), [η^2^-C_5_H_5_]^+^ (**3**), and [η^2^-C_7_H_7_]^+^ (**4**).

In contrast to maximally coordinated metal complexes of cyclic polyenes wherein the entire π-system of the ring is bound, η^2^-coordination to cyclic polyenes that lack rotational symmetry may form multiple constitutional isomers and diastereomers, owing to the asymmetric nature of {WTp­(NO)­(PMe_3_)}. The first milestone of our investigation and the primary subject of this report was to develop methods for the preparation of a family of complexes of the form [WTp­(NO)­(PMe_3_)­(η^2^-C_*n*_H_*n*_)]^*m*+^, where multiple isomers were not anticipated (where *n* = 6 or 8 for *m* = 0, and where *n* = 5 or 7 for *m* = 1). A study of such a series of hydrocarbon complexes would be unparalleled and therefore could offer a new perspective on transition-metal polyene complexes. Hence, we endeavored to synthesize WTp­(NO)­(PMe_3_)­(η^2^-C_8_H_8_) (**2**), [WTp­(NO)­(PMe_3_)­(η^2^-C_5_H_5_)]^+^ (**3**), and [WTp­(NO)­(PMe_3_)­(η^2^-C_7_H_7_)]^+^ (**4**), and compare their fundamental properties with those of WTp­(NO)­(PMe_3_)­(η^2^-C_6_H_6_) (**1**).

## Results and Discussion

The dihapto-coordinated benzene complex **1** is conveniently prepared from W­(CO)_6_ in a four-step procedure on a multigram scale. While this compound is a suitable precursor to other complexes of type WTp­(NO)­(PMe_3_)­(η^2^-L), we have found that the anisole derivative (**5**) is particularly convenient to synthesize on a large scale,^13^ undergoes similar exchange reactions to **1**, and possesses greater thermal stability than the benzene analog (**1**; Figure 2). Therefore, **5** serves as a universal precursor to the desired hydrocarbon complexes. We note that the driving force for this facile substitution reaction is the rearomatization of the benzene ligand.^2^ To synthesize the cyclooctatetraene (COT) complex **2**, a THF solution of **5** was heated with COT for 3 h at 50 °C to drive the replacement of anisole by COT. This reaction mixture was loaded onto a silica column and eluted with ethyl acetate (EtOAc). The product COT complex **2** was precipitated from the solution through the addition of hexanes (yield = 71%). Crystals of **2** were grown from the hexanes filtrate, and its molecular structure was determined through single-crystal X-ray diffraction (SC-XRD; Figure 3). Organic cyclooctatetraene adopts a tub shape to avoid an antiaromatic 8π-electron system and to alleviate angular strain.^14^ However, when reduced, organic COT^2–^ exhibits aromatic planarity. COT has been shown to bind to transition metals as η^8^-COT, η^4^-COT, and η^2^-COT, but most commonly maintains the tub conformation.^15–18^ Similarly to COT^2–^, the η^2^-COT ligand in **2** exhibits semiaromatic planar character:^19,20^ The tungsten causes a flattening of the ring (deviations from the plane are between 0.069(4) and 0.105(4) Å), lengthening in the C=C bonds (1.33 Å to 1.35 Å (mean)), and shortening of C–C bonds (1.47 Å to 1.44 Å (mean)), compared to free COT. These structural changes are consistent with the interpretation of **2** as a tungsten­(II) complex of a semiaromatic [η^2^-C_8_H_8_]^2–^. As seen in the tungsten system, semiaromatic planarity has been observed in crystal structures of Cp_2_Ta­(*n*-isopropyl)­(η^2^-C_8_H_8_),^21^ (d^i^ppe or d^t^bpe)_2_Ni­(η^2^-C_8_H_8_),^22^ and CpMn­(CO)_2_ (η^2^-C_8_H_8_).^23^

**Figure 2:**
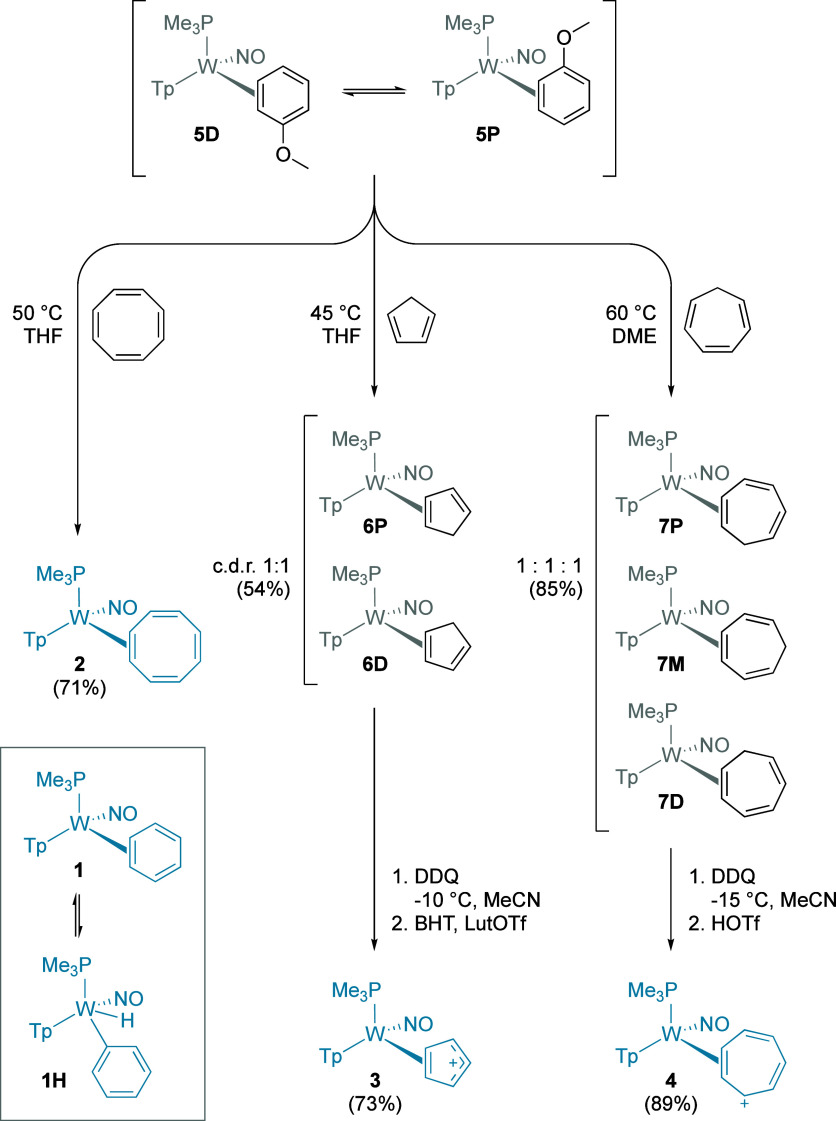
Synthetic details for the target compounds [WTp­(NO)­(PMe_3_)­(η^2^-C_*n*_H_*n*_)]^*m*+^ (where *m* = 0 for *n* = 6 or 8, and *m* = 1 for *n* = 5 or 7). Inset: the analogous benzene complex **1** in equilibrium with its phenyl hydride isomer **1H**.

**Figure 3:**
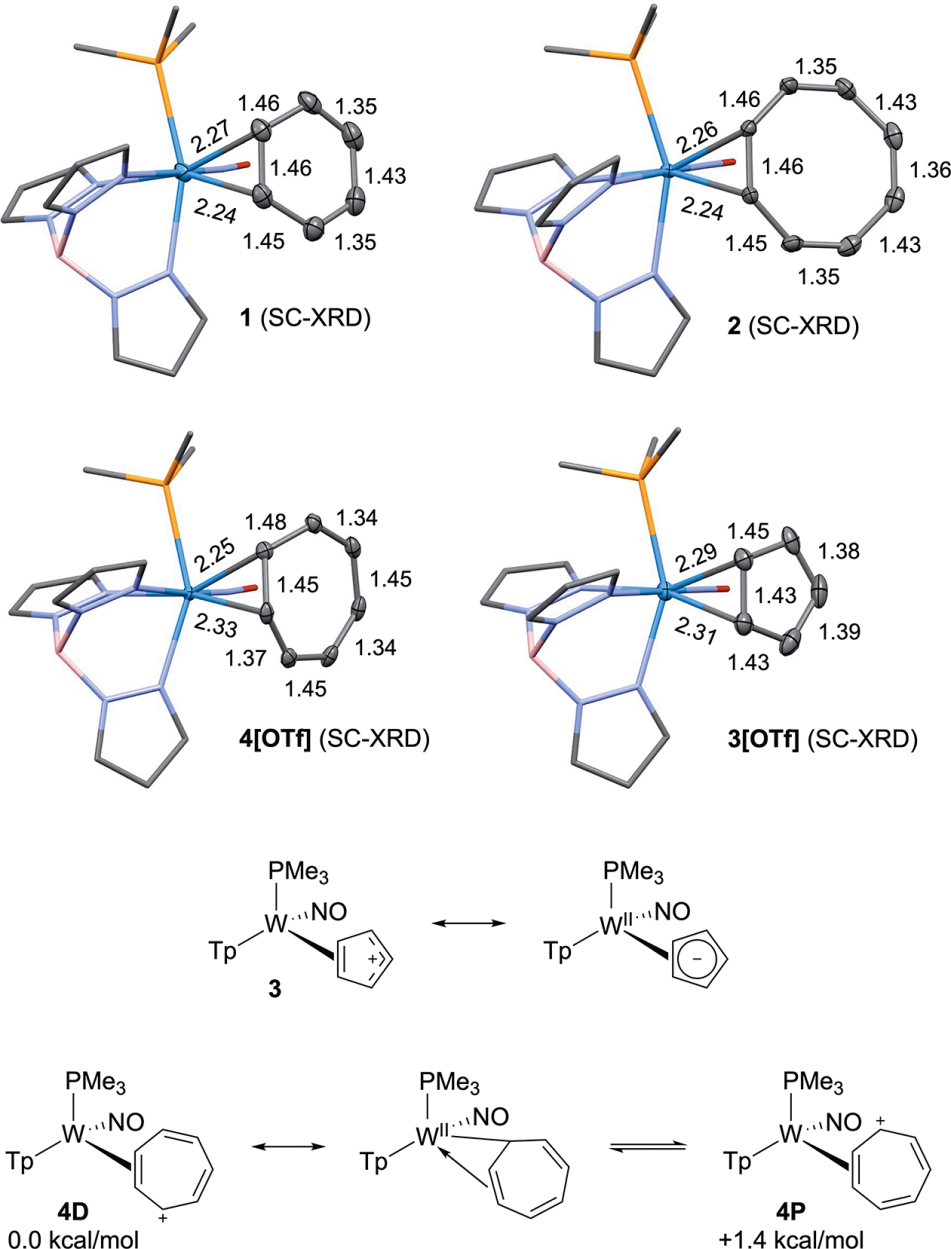
Molecular structures (all distances in Å) for compounds [WTp­(NO)­(PMe_3_)­(η^2^-C_*n*_H_*n*_)]^*m*+^ (where *m* = 0 for *n* = 6 or 8 and *m* = 1 for *n* = 5 or 7; structure of cation [**3**]^+^ is shown from triflate salt), and conformers and resonance contributors for **4**. For clarity, the ancillary ligands are displayed as sticks.

To prepare the cationic dihapto-coordination complexes of [C_5_H_5_]^+^ and [C_7_H_7_]^+^, we envisioned a hydride abstraction from the corresponding η^2^-diene and η^2^-triene complexes, analogous to that observed previously for WTp­(NO)­(PMe_3_)­(η^2^-cyclopentene):^24^ The η^2^-allyl complex [WTp­(NO)­(PMe_3_)­(η^2^-C_5_H_7_)]^+^ can be prepared from WTp­(NO)­(PMe_3_)­(η^2^-C_5_H_8_) and [CPh_3_]­OTf. The cyclopentadiene complex **6** has been reported previously as a mixture of two coordination diastereomers.^25^ Unexpectedly, the treatment of **6** with [CPh_3_]­OTf results in the formation of an electrophilic addition product [WTp­(NO)­(PMe_3_)­(Ph_3_C–C_5_H_6_)]^+^, which was not pursued. After screening several other potential alternative hydride abstractors (e.g., tris­(pentafluorophenyl)­borane, 1,4-benzoquinone, and naphthoquinone), we discovered that 2,3-dichloro-5,6-dicyano-1,4-benzoquinone (DDQ) could convert the diene mixture **6** to the desired cyclopentadienyl complex [**3**]­DDQ, whose molecular structure was confirmed by SC-XRD (crystals were grown by slow evaporation of a DCM solution at room temperature). Based on the lack of OH stretches in IR data, the absence of ^13^C NMR signals, and SC-XRD bond length data, the DDQ-derived counteranion for this product was determined to be the paramagnetic radical anion DDQ^•–^. Presumably, DDQH^–^ produced in the purported hydride abstraction is completely consumed in the presence of excess DDQ to form DDQ^•–^ and DDQH^•^. The neutral DDQH^•^ is likely lost in the filtrate or deprotonated.^26,27^ We thus incorporated the radical scavenger butylated hydroxytoluene into the workup procedure, coupled with 2,6-lutidinium triflate (LutOTf) to neutralize the byproducts of radical quenching and to serve as a source of the (diamagnetic) triflate anion. This anion metathesis was accompanied by a color change of the bulk solid from intense purple-black to light gray, and was verified by ^13^C NMR, IR, and CV. Small single crystals of [**3**]­OTf of markedly higher quality than the DDQ material were obtained from the ethereal filtrate remaining after workup, which confirmed the nature of the anion and allowed for the unambiguous determination of molecular geometry by XRD; all five ring hydrogens were located directly in the electron density map and freely refined. The W–C bond lengths of the dihapto-coordinated carbons are 2.292(3) Å and 2.313(3) Å, while W–C distances of the unbound carbons are 3.011(4) Å, 3.022(4) Å, and 3.400(4) Å. Additionally, bond lengths within the carbon ring are largely symmetric, indicating that there is little preference for charge localization on the carbon “distal” to the phosphine ligand, as previously observed for other η^2^-bound allyls.^25^ These characteristics are consistent with a resonance contributor of **3** where a semiaromatic [C_5_H_5_]^−^ ligand is dihapto-coordinated to W­(II) (Figure 3, **3**). Consistent with this notion, compound **3** is diamagnetic, an observation in contrast to the formally antiaromatic C_5_H_5_^+^ cation, which is computationally predicted to have a paramagnetic ground state.^28^

The cyclopentadienyl cation (Cp^+^) has been studied for its unique properties,^29^ but as a ligand in organometallic complexes it is typically considered to be in its anionic, aromatic form (Cp^–^).^30,31^ Although usually bound as η^5^-Cp and η^3^-Cp, η^1^-Cp complexes have also been observed experimentally.^32–35^ While η^2^-cyclopentadienyl complexes of main-group elements have been structurally characterized (e.g., Al),^36^ compound **3** provides a rare example of a transition metal η^2^-cyclopentadienyl complex. A few η^2^-Cp complexes have been reported with Mn­(II),^37^ but these structures, described as “ring-slipped” or “ambiguous hapticity”, have their distortions attributed to steric factors, and are likely unstable in solution. The compound TiCp_3_ has been shown in the solid state to have one of its Cp rings bound through two carbons.^38^ In no case were we able to find a report of a substitution-inert η^2^-Cp transition metal complex. In contrast, **3** is stable for days in acetonitrile solution.

Direct ligand exchange of tropylium salts for the anisole ligand of **5** were unsuccessful due to the oxidizing properties of this cation.^39^ However, warming a DME solution of cycloheptatriene (CHT) and the anisole complex **5** for 1.5 h (60 °C) resulted in the synthesis of the corresponding CHT complex **7**, which was precipitated from solution by addition of hexanes. NMR data indicate that **7** was isolated as a 1:1:1 mixture of three coordination isomers (Figure 2). Treating this isomeric mixture with DDQ in MeCN followed by an excess of trifluoromethanesulfonic acid (HOTf) resulted in hydride abstraction and anion metathesis, respectively, generating the targeted tropylium species **4** (An improved procedure with 3,6-dichlorotetrazine is also described in the Supporting Information). Upon isolation of this species by precipitation in diethyl ether, crystals of **4** were grown from the filtrate and the molecular structure was determined by SC-XRD (Figure 3). The crystal structure shows that **4** is similar to η^2^-coordinated allyl complexes,^25^ with two short W–C bonds (2.327(2), 2.253(2) Å) and one much longer one (2.772(3) Å). Further, the uncoordinated portion of the π system is distinctly unsymmetric, suggesting a η^1^:η^2^-resonance contributor (Figure 3 and **4D**).^40^ Similar to the bond length changes observed in the η^2^-benzene complex **1**, the tungsten fragment acts as a dearomatization agent in η^2^-tropylium, diminishing the aromatic character of the ring. Whereas the organic tropylium cation has equivalent C–C bonds (1.35 Å),^41,42^ the unbound portion of the ring in compound **4** has alternating long and short C–C bonds (Figure 3). Additionally, the decrease in aromaticity can be seen through the loss of planarity (Supporting Information, deviations from the plane are between 0.023(2) and 0.196(2) Å), as well as a significant upfield-shift in the ring protons of **4** (5.67 ppm c.f., ∼9.3 ppm).^42^ Transition metal complexes of the cycloheptatrienyl cation are typically η^7^ or η^6^,^43–45^ but occasionally have been reported as trihapto-coordinated (η^3^).^46–48^ We are unaware of any reports of structurally characterized η^2^-tropylium complexes, although the structure of an η^2^-alkyne analog of tropylium has been reported.^49,50^

**Figure 4:**
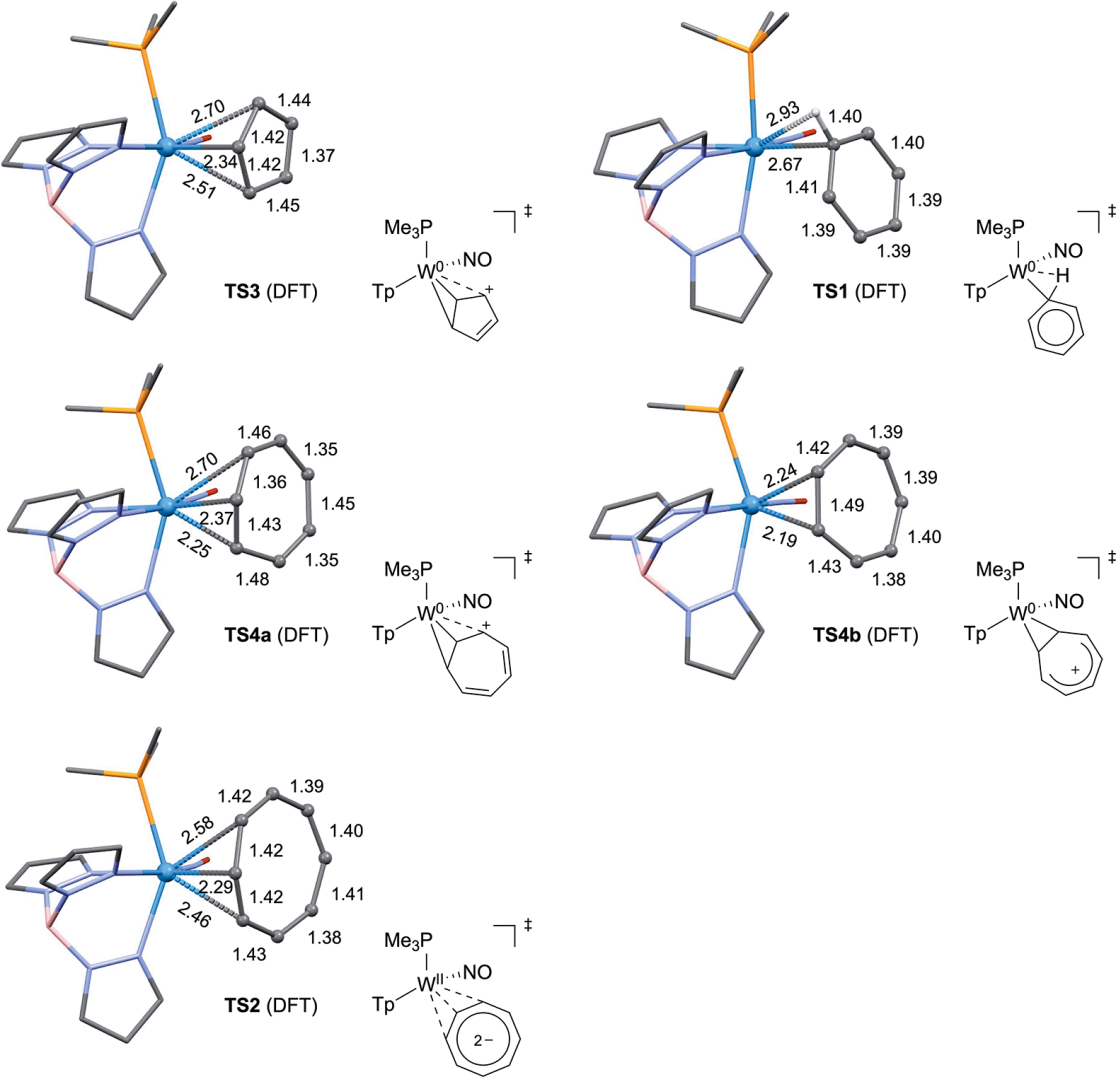
Transition states for ring-walking of compounds [WTp­(NO)­(PMe_3_)­(η^2^-C_*n*_H_*n*_)]^*m*+^ (where for *m* = 0 for *n* = 6 or 8 and *m* = 1 for *n* = 5 or 7). All distances in Å.

### Fluxional Behavior

All four [WTp­(NO)­(PMe_3_)­(η^2^-C_*n*_H_*n*_)]^*m*+^ complexes exhibit fluxional behavior in their ^1^H NMR spectra. In the case of the neutral benzene (**1**) and COT (**2**) complexes, ^1^H NMR spectra are resolved at 25 °C, but elevating the temperature causes coalescence (Figure 4), with free energies of activation determined to be 16 ± 2 and 18 ± 0.9 kcal/mol, respectively (Table 1). In contrast, the cationic complexes **3** and **4** show one doublet (*J*_PH_ ∼2 Hz) corresponding to all the ring protons, and although broadening occurred, the coalesced feature remained to the limit of our experiment (−90 °C), suggesting a very low activation barrier for ring-slipping. The η^2^-benzene **1** was previously studied by Harman and Ess.^51^ ^1^H NMR data for **1** indicate that this species exists in equilibrium with its aryl hydride isomer (**1H**; Figure 2), and replacement of the PMe_3_ ligand by PBu_3_ changes the equilibrium ratio of **1**:**1H** from 10:1 to 1:2.5, a shift attributed largely to entropic considerations.^51^ For WTp­(NO)­(PBu_3_)­(η^2^-benzene) the tungsten hydride was observed to undergo chemical exchange with all ring protons of the dihapto-bound benzene, suggesting the aryl hydride to be a possible participant in the ring-walk mechanism. A DFT study of **1** supported this notion but revealed a very flat potential energy surface near the transition state that includes a ring-slip transition state in which the benzene is fully rearomatized (Figure 4).

**Table 1:** Calculated and Experimentally Observed Activation Free Energies for [WTp­(NO)­(PMe_3_)­(C_*n*_H_*n*_)]^*m*+^ (where *m* = 0 for *n* = 6 or 8 and *m* = 1 for *n* = 5 or 7)

L	*T*_c_ (°C)	Δ*G* ^⧧^_exp_ (kcal/mol)^a^	Δ*G* ^⧧^_calc_ (kcal/mol)^b^
[C_5_H_5_]^+^	<−90	n/a	7.1
C_6_H_6_	35	16 ± 2^c^	12.8
[C_7_H_7_]^+^	<−90	n/a	4.0
C_8_H_8_	50	18 ± 0.9	16.7

a Determined at the coalescence temperature.

b Calculated for 25 °C.

c Determined for the PBu_3_ analog.

A tungsten hydride complex analogous to **1H** was not observed in the ^1^H NMR spectrum of WTp­(NO)­(PMe_3_)­(η^2^-COT) **2**, but we queried whether such a species was energetically accessible. Hence, DFT was used to further study the fluxional properties of the η^2^-polyene complexes **2**–**4**. First, a small benchmarking study was performed (Supporting Information). Functionals and basis sets were chosen based on past studies,^51^ but M06-2X alongside 6-31G­(d,p) with the LANL2DZ effective core potential on W was found to best reproduce the crystallographic data for complexes **1**–**4** (Figure 3). The fluxional behavior of **2**, **3**, and **4** were modeled, essential intermediates and transition states were identified, and an intrinsic reaction coordinate (IRC) energy landscape was calculated for each system (Supporting Information). In the case of the η^2^-COT complex **2**, a κ^1^ transition state (**TS2** in Figure 4; + 16.7 kcal/mol) could be identified as part of a ring-slip mechanism (Figure 4 and Supporting Information), which, in contrast to the benzene analog **1**, was lower in energy than the corresponding hydride intermediates (+17.1 and +27.7 kcal/mol). From the calculated transition state **TS2**, it was also determined that the COT ligand adopts a completely planar structure with C–C bond lengths around the ring approaching 1.4 Å (1.39–1.42 Å)—a structure that is consistent with an aromatic COT dianion (Figure 4).

Similar to many [WTp­(NO)­(PMe_3_)­(η^2^-allyl)] complexes,^25^ the cycloheptatrienyl cation in **4** has two energetically independent conformational isomers where the charge is localized at the “proximal” (**4P**) and “distal” (**4D**) positions. We successfully minimized both conformations of η^2^-tropylium, whose energy difference is 1.36 kcal/mol, favoring **4D** (Figure 3). In addition to a “carbene hydride” species (+17.4 kcal/mol; Supporting Information), two η^2^-C-H complexes were identified (+13.0, +15.0 kcal/mol; Supporting Information), but all three structures were too high energy to be relevant to the ring-slip process. A two-step mechanism was identified, in which the distal conformer **4D** first isomerizes to the proximal form **4P** through an “allyl shift” or “ring-slip” transition state (4.0 kcal/mol; **TS4a** in Figure 4), moving the η^2^-bound carbons by one position. In the second step of the mechanism, **4P** converts again to **4D** through a unique low-energy four-carbon charge transfer transition state (**TS4b** in Figure 4; 1.5 kcal/mol), without changing which two carbons are η^2^-bound. As such, although this second transition state interconnects the same two species (**4D** and **4P**), the identities of the atoms have changed, and it is distinct from **TS4a**. This transition state is also unique because the coordinated ring is mostly planar, with single bonds shortened and double bonds lengthened compared to the ground state (Figure 4).

In contrast to the strongly unsymmetrical η^2^-tropylium complex **4**, the crystal structure for the η^2^-Cp analog **3** resembles a true dihapto-bound ligand with two short tungsten–carbon bonds, and much longer interactions to the adjacent carbons. A small amount of distortion is noted in the uncoordinated portion of the allyl, with one C–C bond having more double bond character than the other, yet separate “distal” and “proximal” conformations could not be computationally located. No carbene-hydride species were identified for **3**, but similar to **4**, two different η^2^-C–H complexes were found at energies (+9.9, +12.6 kcal/mol; Supporting Information) too high to be integral to the ring-slip mechanism. Interestingly, the structure of **TS3** resembles a “proximal” η^2^-allyl species, tightly binding two carbons while more weakly interacting with the third (Figure 4).

### Electrochemical Behavior

Cyclic voltammetric data were collected for hydrocarbon complexes **1–4** in acetonitrile solution using tetrabutylammonium hexafluorophosphate as the electrolyte. The benzene complex **1** features an irreversible anodic wave at −0.13 V NHE at 100 mV/s, at a potential similar to, but lower than, those typically observed for simple alkene complexes of {WTp­(NO)­(PMe_3_)} (c.f., cyclopentene: 0.35 V, NHE).^13^ This is likely a reflection of a much faster rate of hydrocarbon displacement for [W^I^Tp­(NO)­(PMe_3_)­(benzene)]^+^ compared to [W^I^Tp­(NO)­(PMe_3_)­(cyclopentene)]^+^, rather than a difference in the formal reduction potentials of **1** and its cyclopentene analog.^1,2^ Analogously, the η^2^-COT complex **2** shows an anodic wave at 0.37 V (100 mV/s). In contrast to the benzene analog, however, this complex shows an apparent one-electron reduction with an *E*_p,c_ = −2.2 V (100 mV/s).

As expected, the triflate salts of **3** and **4** are far more resistant to oxidation than their neutral counterparts. The η^2^-Cp complex **3** shows its first anodic wave at *E*_p,a_ = 1.26 V, but also features a cathodic wave at *E*_p,c_ = −0.43 V (100 mV/s). By contrast, the η^2^-tropylium complex **4** shows no electrochemical activity between 1.2 V and −1.2 V (NHE). For comparison, typical η^2^-allyl complexes of {WTp­(NO)­(PMe_3_)} show cathodic waves around −0.8 to −1.1 V at 100 mV/s (NHE).^25^ Taken together, these data support the notion that the [C_5_H_5_]^+^ complex **3** is far more prone to ligand reduction compared to typical η^2^-allyl cation complexes of {WTp­(NO)­(PMe_3_)}, possibly leading to an aromatic [C_5_H_5_]^−^. Meanwhile, the tropylium analog **4**, which is already largely aromatic, is more resistant to such a reduction compared to analogous η^2^-allyl cation complexes.

### Methylations

The next phase of this study was to verify that species **2**, **3**, and **4** could be functionalized similarly to species **1**. Methylations were used to demonstrate this synthetic potential. First, we needed to determine whether the reactivity of the η^2^-COT species **2** was analogous to **1**, and whether it could be protonated to form cationic ligands suitable for reactions with nucleophiles. Indeed, the treatment of COT complex **2** with HOTf formed three isomers of the cyclooctatrienyl complex (**8**; see Supporting Information), enriched through equilibration from a ratio of 3:3:1 to 6:1:0. Preliminary experiments of combining **8** with the Grignard reagent MeMgCl indicate that the dominant triene isomer is formed (6:1) and one dominant triene isomer is isolated via acetonitrile precipitation (>20:1; **10**). A similar result was obtained for methylation of the benzene complex **1**. Second, we wanted to verify that the treatment of the cationic complexes **3** and **4** with nucleophiles could generate η^2^-polyene complexes. Treatment of the η^2^-tropylium complex **4** with NaBH_4_ formed two coordination diastereomers of the CHT complex, **7D** and **7P**, in a 1:1 ratio. Notably, the constitutional isomer **7M** was not observed (see Figure 2). In a similar manner, the η^2^-Cp complex **3** reacted with NaBH_4_ to form **6D** and **6P** in a ratio of 2:1. This observation is notable, as the highly unstable antiaromatic (Cp^+^) molecule has been stabilized by the tungsten, allowing it to undergo a nucleophilic addition with hydride. {WTp­(NO)­(PMe_3_)} is an established dearomatization agent, but it apparently can also act as a “de-anti-aromatization agent.” We next added a Grignard reagent (MeMgCl) to the cationic complexes **3** and **4**. In both cases, the addition anti to the metal occurred smoothly, generating two methylated polyene complexes **11** and **12**, respectively (Figure 5). We note that while all four of the methylated complexes **9**–**12** are generated as mixtures of two diastereomers, this is a consequence of the tungsten stereogenic center. The organic ligand for each pair of organometallic diastereomers is identical. Of note, nucleophilic addition to η^5^-Cp complexes is highly unusual,^52^ although the analogous reaction with η^7^-C_7_H_7_ complexes is well established.^53^

**Figure 5:**
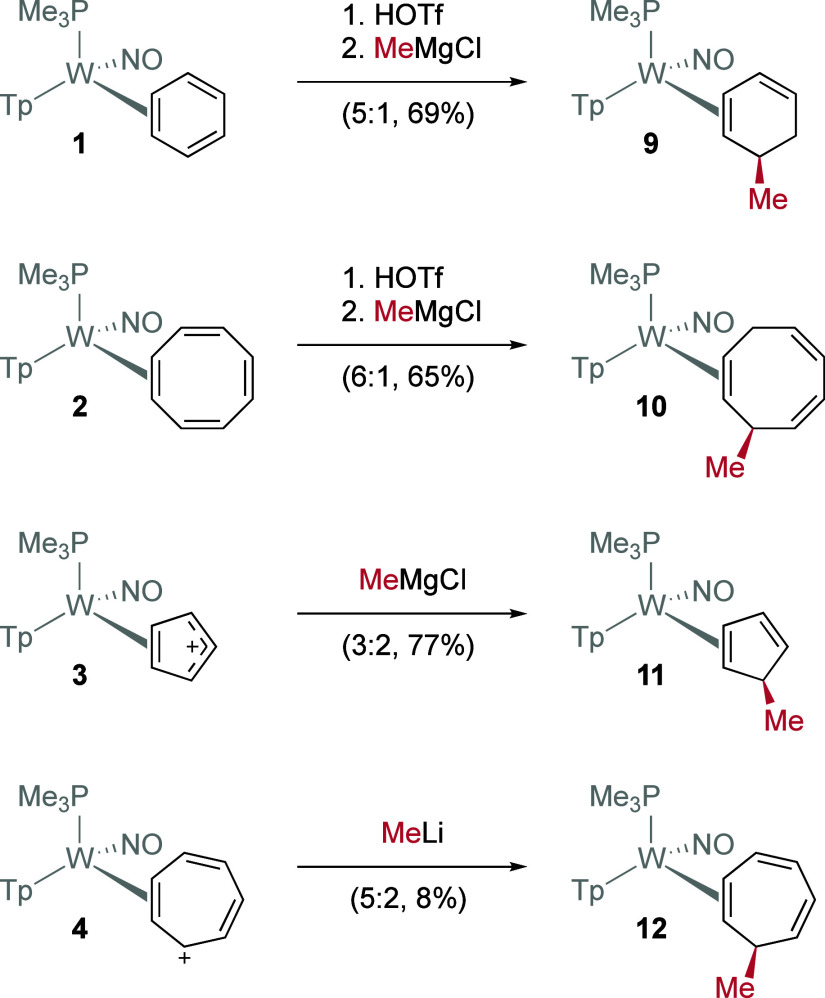
Exploratory methylation reactions with [WTp­(NO)­(PMe_3_)­(η^2^-C_*n*_H_*n*_)]^*m*+^ to generate methylated polyene complexes.

To demonstrate proof of concept, we set out to elaborate the cyclooctatetraene complex **2** into a trisubstituted cyclooctene through the sequential addition of three independent nucleophiles. A full mechanistic investigation of such a process will be disclosed separately, but to demonstrate the synthetic potential of complexes **2**–**4**, we describe the following example, installing three different functionalities on the 8-membered ring: As with the aforementioned methylation, we treated the COT complex **2** with HOTf to yield the equilibrated trienyl complex mixture **8**, which was treated with a benzyl Grignard reagent at room temperature to form a substituted triene complex analogous to **10** (Supporting Information). This was followed by a second addition of proton and nucleophile, in this case NaCN, to generate a disubstituted diene complex (Supporting Information). A final protonation and addition of a vinyl Grignard reagent produced compound **13**, isolated in a diastereomeric ratio of >20:1, with any purported minor diastereomers removed during the isolation procedures. SC-XRD confirms a complex of 3,4,8-trisubstituted cyclooctene in which all three substituents were oriented anti to the metal (Figure 6). Finally, oxidative decomplexation yielded the free organic **14**, a cis, cis-trisubstituted 3,4,8-cyclooctene (dr and ir > 20:1). The overall yield of **14** from the COT complex **2** was 12% over seven steps (74% per step). In analogous fashion, the cyclopentadienyl cation was treated sequentially with a benzyl Grignard reagent, triflic acid, and dimethylmalonate to form a 3,5-disubstituted cyclopentene complex **15** isolated as a 7:4:1 mixture of isomers, which upon oxidative decomplexation yielded the free organic **16** in overall yield from **3** of 4 steps of 19.3% (66% per step; Figure 6; constitutional isomer ratio 10:1). Studies exploring the full scope of such nucleophilic addition sequences for **2**–**4** are currently underway.

**Figure 6:**
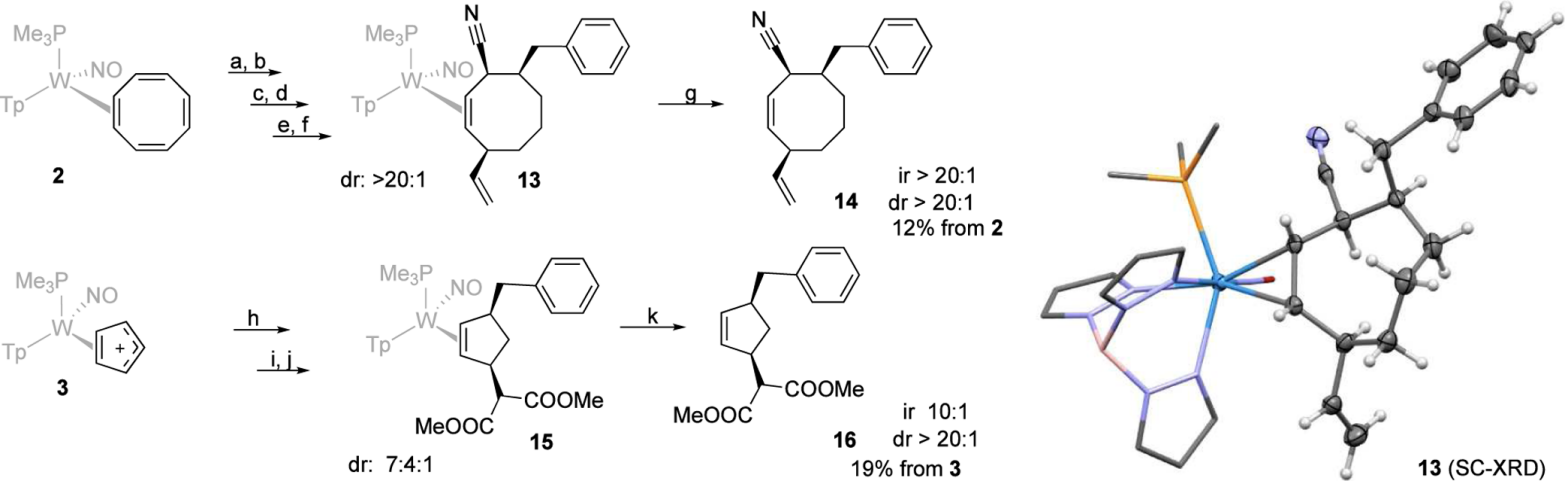
Conversion of COT complex **2** to a cis–cis-3,4,8-trisubstituted cyclooctene complex **13** and oxidative decomplexation of the cyclooctene ligand **14**, and the conversion of cyclopentadienyl complex **3** to cis-3,5-disubstituted cyclopentene complex **15** and its oxidative decomplexation of the cyclopentene **16**. Structure of **13** was confirmed by SC-XRD (hydrogens and 50% thermal ellipsoids shown only on the organic ligand for clarity). Reaction conditions: (a): HOTf/THF (65%). (b): BnMgCl (63%). (c): HOTf/DCM (93%). (d): NaCN (86%) (e): HOTf/DCM (96%). (f): C_2_H_3_MgBr (83%). (g): CAN (47%). (h): BnMgCl (82%). (i): HOTf/MeCN (78%). (j): LiDiMM (72%). (k): CAN (42%).

## Conclusions

With the ultimate goal of developing new methods for the functionalization of cyclic hydrocarbons, it is beneficial to understand the structural and electronic properties of these unique dihapto-coordinated conjugated carbocycles. Structural and electronic properties were studied with SC-XRD, DFT, CV, and NMR. Like the η^2^-benzene complex **1**, the η^2^-cyclooctateraene complex **2** is fluxional at elevated temperatures. In contrast, η^2^-Cp and η^2^-tropylium complexes **3** and **4** are fluxional, but with coalescence occurring far below room temperature. The tungsten fragment used, {WTp­(NO)­(PMe_3_)}, has been successful in the dearomatization and functionalization of several types of aromatic compounds. In particular, WTp­(NO)­(PMe_3_)­(η^2^-benzene) and its derivatives have been transformed into sophisticated organic molecules, and we plan to explore similar chemical pathways with the five- (η^2^-Cp), seven- (η^2^-tropylium), and eight- (η^2^-COT) membered systems. Controlling the addition of nucleophiles and tungsten-alkene isomerizations of these systems will be an important next step in the development of new synthetic methodologies for the preparation of highly functionalized cyclopentanes, cycloheptanes and cyclooctanes based on dihapto-coordinated aromatic (and antiaromatic) hydrocarbons.

## Supplementary Material





## Notes

Synthetic procedures and ^1^H and ^13^C NMR spectra of selected compounds, DFT calculations, and crystallographic information (PDF)
Coordinates of the compounds 2, TS2, 2H-D, 2H-P, 2B, 3, TS3, 3A-D, 3A-P, 4D, 4P, TS4a, TS4b, 4H-P, 4A-D, 4A-P (XYZ)
