# Supplementary material for: Dihapto-Coordinated Conjugated Carbocycles (η2‑C n H n n = 5–8): Blurring the Line Between Aromatic and Antiaromatic Hydrocarbons
Source: J Am Chem Soc. 2025 Jul 24;147(31):28322–30. doi: 10.1021/jacs.5c09111 (PMC12333340; doi:10.1021/jacs.5c09111)
Supplement: Supplementary file 1 [file ja5c09111_si_001.pdf]

------Supplemental Information------

Dihapto-Coordinated Conjugated Carbocycles (η^2-C_nH_n n = 5-8): Blurring the line between aromatic and antiaromatic hydrocarbons

Megan N. Ericson, Josh K. Heman-Ackah, Rachel F. Lombardo, Alvin Q. Meng, Mason R. Ortiz, Sofia E. Megert, Diane A. Dickie, and W. Dean Harman*

Department of Chemistry, University of Virginia, Charlottesville, Virginia 22904, United States

Table of Contents

S1 ········ Supplementary Title Page, Table of Contents
S2 ········ General Procedures
S2 ········ Figure S1: Coupling Data for Tp Labeling
S3 – S14 ········ Experimental Procedures
S15 Compound 2 ········ 1H-NMR and 13C-NMR
S16 Compound 3[DDQ] ········ 1H-NMR and 13C-NMR
S17 Compound 3[OTf] ········ 1H-NMR and 13C-NMR
S18 Compound 4 ········ 1H-NMR and 13C-NMR
S19 Compounds 6D & 6P ········ 1H-NMR and 13C-NMR
S20 Compounds 7D, 7M, & 7P ········ 1H-NMR and 13C-NMR
S21 Compound 7A ········ 1H-NMR and 13C-NMR
S22 Compound 8 ········ 1H-NMR and 13C-NMR
S23 Compound 9 ········ 1H-NMR and 13C-NMR
S24 Compound 10 ········ 1H-NMR and 13C-NMR
S25 Compounds 11D & 11P ········ 1H-NMR and 13C-NMR
S26 Compounds 12D & 12P ········ 1H-NMR and 13C-NMR
S27 Compound 13 ········ 1H-NMR and 13C-NMR
S28 Compound 14 ········ 1H-NMR and 13C-NMR
S29 Compound 16 ········ 1H-NMR and 13C-NMR

S30 – S52 ········ DFT Analysis
S53 – S55 ········ Crystallography
S55 ········ References

General Procedures. NMR spectra were obtained on 500, 600, or 800 MHz spectrometers. Chemical shifts are referenced to tetramethylsilane (TMS) utilizing residual ^{1}H signals of the deuterated solvents as internal standards. Chemical shifts are reported in ppm and coupling constants (*J*) are reported in hertz (Hz). Infrared spectra (IR) were recorded as a solid on a spectrometer with an ATR crystal accessory, and peaks are reported in cm^{-1}. Electrochemical experiments were performed under a nitrogen atmosphere. Most cyclic voltammetric data were recorded at ambient temperature at 100 mV/ s, unless otherwise noted, with a standard three-electrode cell from +1.8 to −1.8 V with a platinum working electrode, acetonitrile solvent, and tetrabutylammonium hexafluorophosphate (TBAH) electrolyte (~1.0 M). All potentials are reported versus the normal hydrogen electrode (NHE) using cobaltocenium hexafluorophosphate ($E_{1/2}$ = −0.78 V, −1.75 V) or ferrocene ($E_{1/2}$ = 0.55 V) as an internal standard. The peak separation of all reversible couples was less than 100 mV. All synthetic reactions were performed in a glovebox under a dry nitrogen atmosphere unless otherwise noted. All solvents were purged with nitrogen prior to use. Deuterated solvents were used as received from Cambridge Isotopes and were purged with nitrogen under an inert atmosphere. When possible, pyrazole protons of the tris(pyrazolyl)borate (Tp) ligand were uniquely assigned (e.g., "Tp3B") using two-dimensional NMR data (see Figure S1). If unambiguous assignments were not possible, Tp protons were labeled as "Pz". BH peaks (around 4−5 ppm) in the ^{1}H-NMR spectra are not assigned due to their quadrupole broadening; However, confirmation of the BH group is provided by IR data (ca 2500 cm^{-1}). All cationic complexes have triflate as the counteranion unless explicitly noted otherwise.

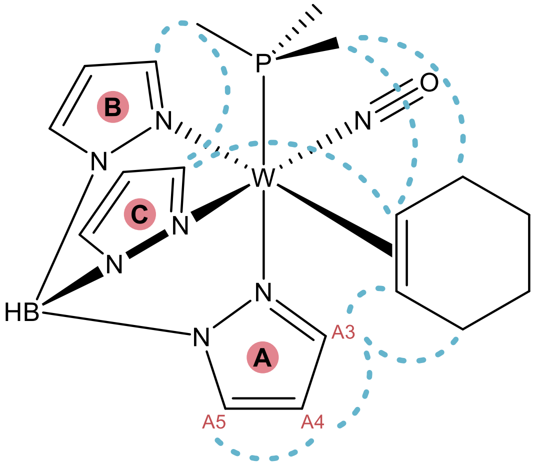

Figure S1: Analysis of NOE and P-H coupling data for Tp labeling.

Characterization of Compounds.

Compounds **1**, **5**, and **9** have been previously reported (see reference 3 and 7 of main text).

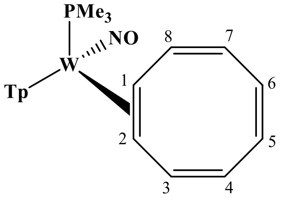

Compound 2. To a 50 mL round bottom flask were added a stir bar, **5** (3.00 g, 3.27 mmol), cyclooctatetraene (2.00 g, 19.2 mmol) and dried THF (6 mL). The round bottom flask was capped with a septum, and the reaction mixture was stirred for 3 hours at 55 °C in an oil bath. Afterwards, a silica column was set up with 60 mL f frit packed with 1 inch of silica over a 500 mL filter flask. The reaction mixture was flushed through with ethyl acetate (300 mL) and dark red decomposition product was caught on the silica. The black-orange filtrate was reduced in vacuo to dryness. The product was redissolved in minimal DCM and precipitated with hexanes (300 mL). The orange solid was collected on a 30 mL fine porosity fritted funnel and desiccated overnight to yield **2** (2.11 g, 70.8%). **^{1}H NMR (800 MHz, CD_3CN, δ, 25 °C)**: 8.34 (d, J = 1.90 Hz, 1H, Pz3A), 8.07 (d, J = 1.92 Hz, 1H, Pz3B), 7.86 (d, J = 2.17 Hz, 1H, Pz5C), 7.82 (d, J = 2.42 Hz, 1H, Pz5B), 7.73 (d, J = 2.42 Hz, 1H, Pz5A), 7.70 (d, J = 2.05 Hz, 1H, Pz3C), 6.42 (dd, J = 5.86, 13.05 Hz, 1H, H8), 6.37 (t, J = 2.27 Hz, 1H, Pz4B), 6.34 (t, J = 2.34 Hz, 1H, Pz4C), 6.33 (m, 1H, H3), 6.20 (t, J = 2.03 Hz, 1H, Pz4A), 5.41 (t, J = 3.40 Hz, 2H, H5/H6), 5.29 (d, J = 13.09 Hz, 1H, H4), 5.21 (d, J = 12.62 Hz, 1H, H7), 2.84-2.80 (m, 1H, H1), 1.66 (t, J = 7.71 Hz, 1H, H2), 1.13 (d, J = 8.58 Hz, 9H, PMe_3). **^{13}C NMR (201 MHz, CD_3CN, δ, 25 °C):** 146.0 (1C, Pz3A), 143.8 (1C, Pz3B), 141.8 (1C, Pz3C), 140.5 (1C, C3), 138.1 (1C, Pz5C), 137.6 (2C, Pz5A/Pz5B), 134.7 (d, J_{PC} = 4.43 Hz, 1C, C8), 124.2 (2C, C5/C6), 120.1 (1C, C7), 118.8 (1C, C4), 107.8 (1C, Pz4B), 107.4 (1C, Pz4C), 106.3 (1C, Pz4A), 56.2 (d, J_{PC} = 9.31 Hz, 1C, C1), 56.0 (1C, C2), 13.6 (d, J_{PC} = 28.70 Hz, 3C, PMe_3). Composition of **2** confirmed by single crystal X-ray diffraction. CV (MeCN, 100 mV/s): Ep,a = +0.37 V, Ep,c = -2.2V (NHE).

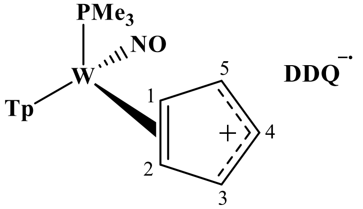

Compound 3[DDQ]. To a test tube were added **6** (129 mg, 0.227 mmol) and 3 mL MeCN. To a second test tube were added a stir bar, DDQ (129 mg, 0.569 mmol), and 2 mL MeCN. Both test tubes were chilled at -10°C in a cold bath for 10 minutes. Next, the solution of **6** was quickly pipetted into the test tube containing DDQ as it remained cold. The dark red reaction mixture was stirred for 15 minutes at -10°C. The reaction mixture was then poured into a 500 mL Erlenmeyer flask with Et_2O (300 mL). A purple precipitate was collected on a 15 mL fine porosity fritted funnel and desiccated under static vacuum to yield **3** as a salt with $DDQ^{\bullet-}$ (148 mg, 82.1%). **^{1}H NMR (800 MHz, CD_3CN, δ, 25 °C):** δ 8.60 (d, J = 2.1 Hz, 1H, Pz), 8.43 (d, J = 2.2 Hz, 1H, Pz), 8.04 (d, J = 2.4 Hz, 1H, Pz), 7.97 (d, J = 2.0 Hz, Pz), 7.81 (d, J = 2.2 Hz, 1H), 7.78 (d, J = 2.4 Hz, 1H, Pz), 6.89 (d, J_{PH} = 2.4 Hz, 5H, H1-5), 6.63-6.33 (t, J = 2.3 Hz, 3H, Pz4A/Pz4B/Pz4C), 1.22 (d, J = 10.2 Hz, 9H, PMe_3). **^{13}C NMR (201 MHz, CD_3CN, δ, 25 °C):** 148.0 (1C, Pz), 146.9 (1C, Pz), 144.7 (1C, Pz), 140.0 (1C, Pz), 139.7 (1C, Pz), 138.4 (1C, Pz), 129.2 (m, 5C, C1-C5), 110.6-108.3 (3C, Pz4A/Pz4B/Pz4C), 13.7 (d, J_{PC} = 33.2 Hz, 3C, PMe_3). Composition of **3[DDQ]** confirmed by single crystal X-ray diffraction. APCI-HRMS (m/z): $[M]^+$ calculated for 569.1450; found 569.1476. CV (MeCN, 50 mV/s): Ep,a = +1.26 V, E1/2 = +0.70 and -0.09V, Ep,c = -0.431 V (NHE).

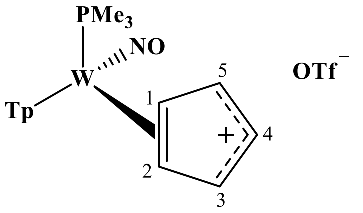

Compound 3[OTf]. For this procedure, all glassware was flame-dried prior to use. To a 60 mL test tube with a stir bar was added **6** (300 mg, 527 μmol, 1 equiv.) and dry MeCN (4 mL). Separately, solutions of DDQ (269 mg, 1.19 mmol, 2.25 equiv.) in dry MeCN (1.5 mL), and butylated hydroxytoluene (290 mg, 1.32 mmol, 2.5 equiv.) and 2,6-lutidinium triflate (143 mg, 556 μmol, 1.05 equiv.) in dry MeCN (5 mL) were prepared in test tubes. All three solutions were chilled to -10 °C. The DDQ solution was transferred to a 3 mL syringe and added *rapidly* to the solution of **6** under maximal stirring, which caused the yellow-brown solution to instantly darken to purple black. After 5 seconds the BHT/lutidinium mixture was added, accompanied by a subtle shift in tint to green/brown black over 30 seconds. After stirring for 1 minute, the solution was precipitated into stirring Et_2O (250 mL, -30 °C), and the gray precipitate collected over a 30 mL fine-porosity fritted disk. Storage of the yellow-brown filtrate over 2 hours yielded numerous clusters of very small black needles suitable for single-crystal XRD analysis on the sidewall of the flask. The precipitate was rinsed through the fritted disk with dry MeCN (5 mL total) into a clean flask and re-precipitated into stirring Et_2O (250 mL, -30 °C). The resulting precipitate was collected over a 30 mL fine-porosity fritted disk, rinsed with Et_2O (50 mL) and pentane (25 mL), and desiccated under active vacuum to yield **3[OTf]** (275 mg, 73%) as a light gray, chunky powder. **^{1}H NMR (600 MHz, CD_3CN, δ, 25 °C):** 8.63 (d, J = 2.3 Hz, 1H, Pz), 8.43 (d, J = 2.3 Hz, 1H, Pz), 8.04 (d, J = 2.4 Hz, 1H, Pz), 7.97 (d, J = 2.5 Hz, 1H, Pz), 7.81 (d, J = 2.3 Hz, 1H, Pz), 7.78 (d, J = 2.5 Hz, 1H, Pz), 6.89 (d, J_{PH} = 2.3 Hz, 5H, H1-H5), 6.64 (t, J = 2.3 Hz, 1H, Pz), 6.55 (t, J = 2.3 Hz, 1H, Pz), 6.33 (t, J = 2.4 Hz, 1H, Pz), 1.23 (d, J_{PH} = 10.2 Hz, 9H, PMe_3). **^{13}C NMR (201 MHz, CD_3CN, δ, 25 °C):** 148.09 (Pz), 146.94 (Pz), 144.79 (Pz), 140.08 (Pz), 139.79 (d, J = 3.1 Hz, Pz), 138.41 (Pz), 129.03 (C1-C5), 122.17 (q, J_{CF} = 320.7 Hz, F_3CSO_3), 110.58 (Pz), 108.98 (Pz), 108.38 (d, J = 5.0 Hz, Pz), 13.72 (d, J_{PC} = 33.7 Hz, PMe_3). IR (ATR, cm^{-1}): ν(BH) 2513.90, ν(NO) 1647.79, ν(F_3CS–O_3) 1260.46, ν(F_3CS–O_3) 1029.59, δ(F_3–CSO_3) 636.01. CV (MeCN, 50 mV/s): $E_{p,c}$ = -0.460 V (NHE). Composition of **3[OTf]** confirmed by single-crystal X-ray diffraction.

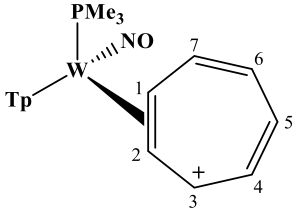

Compound 4[OTf].

From DDQ. In a test tube charged with a stir bar, **7** (100 mg, 0.17 mmol) was dissolved in 1.5 mL MeCN and allowed to stir at -30 °C. Another test tube containing DDQ (42 mg, 0.19 mmol) dissolved in 1 mL MeCN was also chilled at -30 °C. After 10 minutes, the DDQ solution was added to the complex. After 12 hours, HOTf (0.27mmol) was added to the stirring solution in the cold bath. The solution was then immediately precipitated in chilled ether. The precipitate was collected on a 60 mL F frit (154mg). 50mg of the precipitate collected was dissolved in MeCN and reprecipitated in a stirring solution of room temperature ether. Compound **4** was collected on a 60 mL F frit and dried *in vacuo* (36mg, 89%).

From dichlorotetrazine. In a test tube charged with a stir bar, **7** (200 mg, 0.34 mmol) was dissolved in 5.0 mL $CHCl_3$ and allowed to stir at -30 °C. Another test tube containing 3,6-dichloro-1,2,4,5-tetrazine (76 mg, 0.50 mmol) dissolved in 2.4 mL $CHCl_3$ was chilled at -30 °C. After 10 minutes, the 3,6-dichloro-1,2,4,5-tetrazine solution was added to the complex. After 20 minutes, a chilled solution of HOTf in diethyl ether (0.5M, 1.3 mL) was added to the reaction mixture. The reaction mixture was then allowed to stir at -30 °C. After 10 minutes, the reaction mixture was transferred into 200 mL of stirring diethyl ether. A dark grey solid immediately precipitated. The precipitate was collected on a fritted disk, washed with diethyl ether (50mL), and dried desiccated under active vacuum. (223 mg, 0.30 mmol, 89% yield)

^{1}H NMR (800 MHz, CD_3CN, δ, 25 °C): 8.14 (d, J = 2.1 Hz, 1H, Pz), 8.09 (d, J = 2.3 Hz, 1H, Pz), 8.01 (d, J = 2.2 Hz, 1H, Pz), 7.87 (d, J = 2.2 Hz, 1H, Pz), 7.85 (d, J = 2.2 Hz, 1H, Pz), 7.74 (d, J = 2.2 Hz, 1H, Pz), 6.53 (t, J = 2.3 Hz, 1H, Pz), 6.45 (t, J = 2.3 Hz, 1H, Pz), 6.32 (t, J = 2.3 Hz, 1H, Pz), 5.67 (d, J_{PH} = 2.0 Hz, J_{WH} = 20.0 Hz, 7H, H1-H7) 1.21 (d, J_{PH} = 9.6 Hz, 9H, PMe_3). **^{13}C NMR (201 MHz, CD_3CN, δ, 25 °C):** 146.6 (Pz), 145.2 (Pz), 142.6 (Pz), 139.4 (Pz), 139.1 (Pz), 139.7 (Pz), 123.3 (C1-C7), 108.6 (Pz), 108.9 (Pz), 107.6 (Pz), 12.9 (d, J_{PC} = 32.0 Hz, PMe_3). Composition of **4[OTf]** confirmed by single crystal X-ray diffraction.

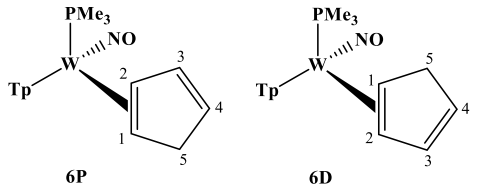

Compounds 6P and 6D. To a 50 mL round bottom flask were added a stir bar, **5** (2.00 g, 3.27 mmol), and 30 mL dried THF. The round bottom flask was sealed with a rubber septum and placed into an oil bath heated to 45°C, yielding a yellow suspension. Immediately, freshly distilled cyclopentadiene monomer (2.81 mL, 2.27 g, 34.3 mmol) was syringed through the septum and into the flask. After stirring for 14 hours, the dark brown reaction mixture was poured into a 500 mL Erlenmeyer flask, and hexanes (350 mL) was added. A purple precipitate was filtered off in a 60 mL fine porosity fritted funnel over a 500 mL filter flask. The tan-colored filtrate was reduced in vacuo to ~100 mL, inducing precipitation. A whitish-tan solid was collected on a 15 mL fine porosity fritted funnel and desiccated under static vacuum to yield **6** (1.01 g, 54.2%) as a ~2:1 ratio of 6P to 6D. **6P** (Major isomer): **^{1}H NMR (800 MHz, CD_3CN, δ, 25 °C):** 8.37 (d, J = 2.0 Hz, 1H, Pz), 8.00 (d, J = 2.0 Hz, 1H, Pz), 7.87 (d, J = 2.4 Hz, 1H, Pz), 7.80 (d, J = 2.4 Hz, 1H, Pz), 7.76 (d, J = 2.4 Hz, 1H, Pz), 7.41 (d, J = 2.2 Hz, 1H, Pz), 6.38 (dd, J = 5.1, 2.2 Hz, 1H, H3), 6.36 (t, J = 2.2 Hz, 1H, Pz), 6.30 (t, J = 2.2 Hz, 1H, Pz), 6.22 (t, J = 2.2 Hz, 1H, Pz), 5.08 (dt, J = 4.9, 2.3 Hz, 1H, H4), 4.33 (ddq, J = 19.3, 6.1, 2.0 Hz, 1H, H5), 3.80 (dt, J = 19.4, 2.2 Hz, 1H, H5), 3.70 (ddt, J = 11.9, 7.4, 2.1 Hz, 1H, H2), 1.90 (td, J = 6.8, 2.9 Hz, 1H, H1), 1.25 (d, J = 8.4 Hz, 9H, PMe_3). **^{13}C NMR (201 MHz, CD_3CN, δ, 25 °C):** 144.0 (1C, Pz), 141.5 (1C, Pz), 140.7 (1C, Pz), 136.9 (d, J_{PC} = 2.9 Hz, 1C, C3), 136.8 (1C, Pz), 136.0 (1C, Pz), 135.6 (1C, Pz), 121.5 (1C, C4), 106.4 (1C, Pz), 105.8 (1C, Pz), 105.7 (1C, Pz), 67.6 (d, J_{PC} = 10.9 Hz, 1C, C2), 56.3 (1C, C1), 42.5 (1C, C5), 13.3 (d, J_{PC} = 28.4 Hz, 3C, PMe_3). **6D** (Minor isomer): **^{1}H NMR (800 MHz, CD_3CN, δ, 25 °C):** 8.16 (d, J = 2.0 Hz, 1H, Pz), 8.00 (d, J = 2.0 Hz, 1H, Pz), 7.88 (d, J = 2.4 Hz, 1H, Pz), 7.80 (d, J = 2.4 Hz, 1H, Pz), 7.74 (d, J = 2.4 Hz, 1H, Pz), 7.41 (d, J = 2.2 Hz, 1H, Pz), 6.57 (dq, J = 4.2, 2.0 Hz, 1H, H3), 6.37 (t, J = 2.2 Hz, 1H, Pz), 6.30 (t, J = 2.2 Hz, 1H, Pz), 6.22 (t, J = 2.2 Hz, 1H, Pz), 5.04 (dt, J = 4.9, 2.4 Hz, 1H, H4), 4.40 (ddt, J = 19.2, 6.8, 2.2 Hz, 1H, H5), 3.44 (m, 1H, H5), 3.40 (m, 1H, H1), 2.17 (dq, J = 7.5, 2.2 Hz, 1H, H2), 1.22 (d, J = 8.3 Hz, 9H, PMe_3). **^{13}C NMR (201 MHz, CD_3CN, δ, 25 °C):** 144.1 (1C, Pz), 143.6 (1C, Pz), 141.2 (1C, Pz), 138.0 (1C, C3), 136.6 (1C, Pz), 136.2 (1C, Pz), 135.4 (1C, Pz), 121.2 (1C, C4), 106.5 (1C, Pz), 106.0 (1C, Pz), 105.9 (1C, Pz), 65.7

(1C, C2), 58.0 (d, J_{PC} = 12.5 Hz, 1C, C1), 42.8 (d, J_{PC} = 2.2 Hz, 1C, C5), 12.8 (d, J_{PC} = 28.0 Hz, 3C, PMe_3). Composition of **6P** confirmed by single crystal X-ray diffraction.

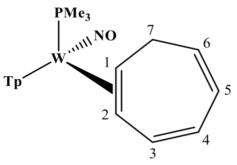

7D

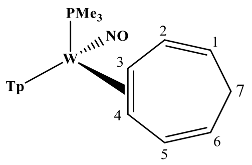

7M

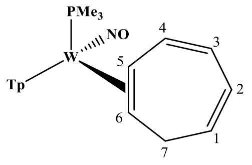

7P

Compounds 7D, 7M, and 7P. In an oven-dried round-bottom flask charged with a stir bar, **5** (5.00 g, 8.18 mmol, 1 equiv.) was combined with 1,3,5-cycloheptatriene (11.3 g, 123 mmol, 15 equiv.) and 1,2-dimethoxyethane (DME, 10 mL). The round-bottom was capped with a septum and stirred in an oil bath at 60 °C for 1.5 h, at which point the reaction was complete by ^{13}P-NMR. A silica plug was packed with hexanes on a 150 mL medium-porosity fritted disk, onto which the reaction mixture was transferred. The plug was eluted with hexanes (300 mL) to remove excess CHT; the filtrate was discarded. Next, the product was eluted as a yellow solution with Et_2O (2 L) followed by DME (500 mL). The solution was reduced to dryness *in vacuo*. The residue was dissolved in minimal DCM, precipitated into hexanes (500 mL), filtered over a 60 mL fine-porosity fritted disk, and desiccated to yield 2.91 g (60.%) of an isomeric mixture of **7** as a light tan solid. IR (ATR, cm^{-1}): ν(NO) = 1561. CV (MeCN, 100 mV/s): Ep,a = +0.37 V (NHE). APCI-HRMS (m/z): $[M]^{+H}$ calculated for $C_{19}H_{28}BN_7OPW^+$, 596.1690; found 596.1673.

Isolation of **7D**: To a 4-dram vial charged with a stir bar, all isomers of **7** (100 mg, 0.17 mmol) dissolved in $CHCl_3$ were left to stir outside of the glovebox for a full 24 h, loosely covered with an unscrewed cap to prevent evaporation. The reaction mixture was precipitated in 300 mL hexanes. A brown precipitate was filtered, and the hexanes filtrate was reduced by 75%. The white solid **7D** formed from the filtrate was filtered over a 30 mL F frit and placed in the desiccator (48 mg, 08 mmol, 48% yield). **^{1}H NMR (800 MHz, $CDCl_3$, δ, 25 °C):** 8.31 (d, J = 1.4 Hz, 1H, PzA3), 8.04 (d, J = 1.4 Hz, 1H, PzB3), 7.70 (d, J = 2.4 Hz, 1H, Pz5B), 7.69 (d, J = 1.7 Hz, 1H, Pz5C), 7.64 (d, J = 2.4 Hz, 1H, PzA5), 7.21 (d, J = 1.7 Hz, 1H, PzC3), 6.28 (d, J = 2.2 Hz, 1H, PzB4), 6.25 (d, J = 2.2 Hz, 1H, PzA4), 6.15 (d, J = 2.2 Hz, 1H, PzC4), 7.03 (dd, J = 11.0, 3.5 Hz, 1H, H3), 6.21-6.18 (m, 1H, H5), 6.10-6.06 (m, 1H, H6), 5.76 (dd, J = 11.0, 5.4 Hz, 1H, H4), 3.18-3.08 (m, 2H, H7), 2.83-2.77 (m, 1H, H1), 1.46-1.41 (m, 1H, H2), 1.26 (d, J_{PH} = 8.1 Hz, 9H, PMe_3). **^{13}C NMR (201 MHz, $CDCl_3$, δ, 25 °C):** 143.8 (PzA3), 143.5 (PzB3), 137.5-135.0 (2C, PzB5/PzC5), 136.2 (PzA5), 139.9 (PzC3), 107.7-105.0 (3C, PzB4/PzA4/PzC4), 141.90 (C3), 128.6 (C5), 128.70 (C6), 122.74 (C4), 34.0 (C7), 55.6 (C1), 55.0 (C2), 14.3 (d, J_{PC} = 27.3 Hz, 3C, PMe3). ^{31}P NMR (500 MHz, DME, δ, 25 °C): J_{WP} = 144.8 Hz.

Isolation of **7M**: After protonation of **7** with HOTf, the resultant WTp(NO)(PMe_3)(η^2-cycloheptadienyl) triflate salt **7A** (160 mg, 0.21 mmol) was dissolved in 3 mL $CHCl_3$ and allowed to stir at -15 °C. Another test tube containing triethylamine (TEA) (0.30 mL, 2.15 mmol) dissolved in 1 mL $CHCl_3$ was also chilled at -15 °C. After 10 minutes, the TEA test tube was added to the test tube containing the complex and the solution was allowed to stir for 15 minutes. Upon removal from the bath, the solution was diluted with 30 mL of $CHCl_3$ and extracted 5x with 30 mL of 1 M NaOH. The organic layer was dried with Na_2SO_4, filtered, and evaporated in vacuo until dryness. The dark brown material was redissolved in $CHCl_3$ and evaporated three times to remove excess TEA. The remaining material was dissolved in $CHCl_3$ and precipitated in 300 mL chilled hexanes. The black precipitate was collected on a 15 mL F frit and discarded. The hexanes filtrate was reduced by 150 mL. The white precipitate formed **7M** was filtered, dried in the desiccator, and collected on a 15 mL F frit (45 mg, 0.08 mmol 35%). **^{1}H NMR (800 MHz, $CDCl_3$, δ, 25 °C):** 8.51 (d, J = 2.0 Hz, 1H, PzA3), 8.06 (d, J = 2.0 Hz, 1H, PzB3), 7.70 (d, J = 2.3 Hz, 1H, PzB5), 7.68 (d, J = 2.4 Hz, 1H, PzA5), 7.59 (d, J = 2.4 Hz, 1H, PzC5), 7.31 (d, J = 2.2 Hz, 1H, PzC3), 6.62-6.56 (m, 2H, H5/H6), 6.28 (t, J = 2.1 Hz, 1H, PzB4), 6.20 (t, J = 2.2 Hz, 1H, PzC4), 6.17 (t, J = 2.2 Hz, 1H, PzA4), 5.24 (dtd, J = 6.9,

4.0, 1.4 Hz, 1H, H1), 5.19 (dtd, J = 6.9, 4.0, 1.4 Hz, 1H, H2), 4.36 (dq, J = 20.9, 2.9 Hz, 1H, H7), 2.79 – 2.73 (m, 1H, H3), 2.72 (dt, J = 21.0, 6.9 Hz, 1H, H7), 1.53 – 1.44 (m, 1H, H4), 1.25 (d, J_{PH} = 8.3 Hz, 9H, PMe3). **^{13}C NMR (201 MHz, CDCl3, δ, 25 °C):** 143.6 (PzA3), 143.4 (PzB3), 137.5-135.0 (2C, PzB5/PzA5), 137.8 (PzC5), 140.3 (PzC3), 107.7-105.0 (3C, PzB4/PzA4/PzC4), 137.5/133.7 (C5/C6), 122.5 (C2), 123.4 (C3), 30.1 (C7), 55.6 (C3), 53.85 (C4), 13.8 (d, J_{PC}= 17.8 Hz, 3C, PMe3). 31P NMR (500 MHz, DME, δ, 25 °C): J_{WP} = 147.5 Hz. Composition of **7M** confirmed by single crystal X-ray diffraction.

Attempts to isolate **7P** from its isomers were unsuccessful. **^{1}H NMR (800 MHz, CDCl$_3$, δ, 25 °C):** 8.15 (d, J = 2.0 Hz, 1H, PzA3), 8.06 (d, J = 1.7 Hz, 1H, PzB3), 7.70 (d, J = 1.7 Hz, 1H, Pz5B), 7.61 (d, J = 2.4 Hz, 1H, Pz5C), 7.59 (d, J = 2.4 Hz, 1H, PzA5), 7.23 (d, J = 2.0 Hz, 1H, PzC3), 6.28 (d, J = 2.2 Hz, 1H, PzB4), 6.22 (d, J = 2.2 Hz, 1H, PzA4), 6.17 (d, J = 2.2 Hz, 1H, PzC4), 6.86 (dd, J = 11.3, 4.5 Hz, 1H, H4), 6.10-6.05 (m, 1H, H2), 6.05-6.01 (m, 1H, H1), 5.61 (dd, J = 11.5, 5.6 Hz, 1H, H3), 3.54 (td, J = 14.5, 4.5, 1H, H7), 3.21-3.17 (m, 1H, H7), 2.80 (td, J = 21.0, 6.8 Hz, 1H, H5), 1.87-1.83 (m, 1H, H6), 1.26 (d, JPH = 8.1 Hz, 9H, PMe$_3$). **^{13}C NMR (201 MHz, CDCl$_3$, δ, 25 °C):** 142.8 (PzA3), 143.9 (PzB3), 137.5-135.0 (PzB5), 135.6, (PzC5) 135.4 (PzA5), 140.0 (PzC3), 106.6-105.0 (3C, PzB4/PzA4/PzC4), 140.3 (C4), 137.0 (C2), 135.4 (C1), 123.3 (C3), 32.1 (C7), 55.9)C5), 67.4 (C6), 13.6 (d, J_{PC}= 17.6, Hz, 3C, PMe$_3$). ^{31}P NMR (500 MHz, DME, δ, 25 °C): J_{WP} = 145.1 Hz. CV (MeCN, 100 mV/s): Ep,a = +0.37 V (NHE).

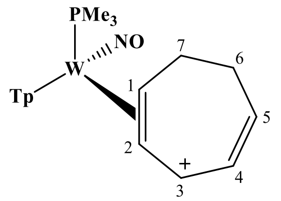

Compound 7A. To a 4-dram vial charged with a stir bar, compounds **7** (150 mg, 0.25 mmol) were dissolved in CHCl$_3$. HOTf diluted in MeCN was added (0.6 mM, 0.30 mmol) and the solution was allowed to stir at 25 °C for 24 h. The solution was pipetted into chilled hexanes. The precipitate obtained was dried in the desiccator and collected on a 30 mL F frit (164.4 mg, 0.22 mmol, 87.5%). **^{1}H NMR (800 MHz, CDCl$_3$, δ, 25 °C):** 8.28 (d, J = 2.2 Hz, 1H, PzB3), 8.19 (d, J = 2.4 Hz, 1H, PzC3), 8.07 (d, J = 2.2 Hz, 1H. PzA3), 7.86 (d, J = 2.4 Hz, 1H, PzC5), 7.83 (d, J = 2.5 Hz, 1H, PzB5), 7.70 (d, J = 2.5 Hz, 1H, PzA5), 6.61 (t, J = 2.3 Hz, 1H, PzC4), 6.54 (ddd, J = 10.5, 5.6, 2.6 Hz, 1H, H4), 6.48 (t, J = 2.3 Hz, 1H, PzB4), 6.36 (td, J = 9.5, 4.3 Hz, 1H, H5), 6.31 (t, J = 2.3 Hz, 1H, PzA4), 6.13 (dd, J = 6.5, 7.5 Hz, 1H, H3), 5.24 (t, J = 9.0 Hz, 1H, H2), 4.99 – 4.92 (m, 1H, H1), 3.17 (td, J = 10.9, 5.2 Hz, 1H, H7), 3.02 (t, J = 13.3 Hz, 1H, H7), 2.63 – 2.57 (m, 1H, H6), 2.37-2.32 (m, 1H, H6), 1.30 – 1.24 (m, 1H), 1.21 (d, J_{PC} =9.3 Hz, 9H, PMe$_3$). **^{13}C NMR (201 MHz, CDCl$_3$, δ, 25 °C):** 145.0 (PzB3), 143.5 (PzC3), 146.9 (PzA3), 137.9-138.5 (3C PzC5/PzB5/PzA5), 109.3 (PzC4), 108.6 (PzB4), 107.4 (PzA4), 124.4 (C4), 138.4 (C5), 126.8 (C3), 109.5 (C2), 77.9 (C1), 36.8 (C7), 31.3 (C6). 14.1 (9C, J_{PH} = 32.2 Hz, PMe$_3$). Composition of **7A** confirmed by single crystal X-ray diffraction.

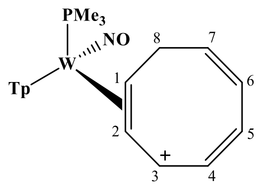

Compound 8. In a 25 mL Erlenmeyer flask charged with a stir bar, **2** (1.00 g, 1.65 mmol) was dissolved in 10 mL dried THF. Next, HOTf (294 mg, 1.96 mmol) was syringed into a test tube with 1 mL dried THF. The HOTf solution was pipetted into the stirring solution of **2** and was let stir for 15 minutes at R.T. as orange precipitate began to form. To further induce precipitation, the crude reaction mixture was chilled at -30°C for 12 hours. Afterward, the orange precipitate **8** was collected as a triflate salt on a 15 mL fine porosity fritted funnel, rinsed with dried THF, and dried in the desiccator (810 mg, 64.8%). **^{1}H NMR (800 MHz, CD_3CN, δ, 25 °C):** 8.33 (d, *J* = 2.02 Hz, 1H, Pz3B), 8.23 (d, *J* = 2.49 Hz, 1H, Pz5A), 8.00 (d, *J* = 2.46 Hz, 1H, Pz5B), 7.96 (d, *J* = 2.46 Hz, 1H, Pz5C), 7.85 (d, *J* = 1.88 Hz, 1H, Pz3C), 7.83 (d, *J* = 2.46 Hz, 1H, Pz3A), 6.88 (dd, *J* = 4.92, 11.64 Hz, 1H, H4), 6.52 (t, *J* = 2.35 Hz, 1H, Pz4C), 6.51 (t, *J* = 2.41 Hz, 1H, Pz4B), 6.49 (dd, *J* = 7.92, 10.45 Hz, 1H, H7), 6.36 (t, *J* = 2.45 Hz, 1H, Pz4A), 6.33 (dd, *J* = 4.68, 9.66 Hz, 1H, H3), 6.15 (dd, *J* = 3.97, 10.67 Hz, 1H, H6), 6.04 (dd, *J* = 3.85, 11.80 Hz, 1H, H5), 4.91 (t, *J* = 9.62 Hz 1H, H2), 4.40-4.35 (m, *J* = 8.41, 16.97, 24.16 Hz, 1H, H1), 3.60 (dt, *J* = 8.84, 13.07 Hz, 1H, H8), 2.48 (dt, *J* = 7.58, 14.09 Hz, 1H, H8), 1.16 (d, *J* = 9.88 Hz, 9H, PMe_3). **^{13}C NMR (201 MHz, CD_3CN, δ, 25 °C):** 148.0 (1C, Pz5A), 146.1 (1C, Pz3B), 144.3 (1C, C7), 143.5 (1C, Pz3C), 139.7-139.5 (3C, Pz5C/Pz5B/Pz3A), 133.3 (1C, C3), 131.7 (1C, C5), 130.2 (1C, C4), 129.5 (1C, C6), 109.6-108.9 (2C, Pz4C/Pz4B), 108.1 (1C, Pz4A), 106.5 (d, J_{PC} = 13.28 Hz, 1C, C2), 73.5 (d, J_{PC} = 11.96 Hz, 1C, C1), 30.7 (d, J_{PC} = 3.73 Hz, 1C, C8), 13.3 (d, J_{PC} = 33.1 Hz, 3C, PMe_3). Composition of **8** confirmed by single crystal X-ray diffraction. ESI-HRMS (m/z): $[M]^+$ calculated for 608.1690; found 608.1697.

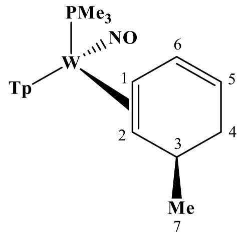

Compound 9. In an oven dried test tube charged with a stir pea, **1** (0.101 g, 0.17 mmol) was dissolved in 5 mL DCM. In another oven dried test tube charged with a stir pea, Diphenylammonium triflate (DPhAT; 0.062 g, 0.19 mmol) was dissolved in DCM. Both test tubes were chilled to -30°C for 20 minutes. The DPhAT solution was added to the solution of **1** and stirred for 30 minutes. The solution was precipitated into chilled ether and desiccated to yield WTp(NO)(PMe_3)(η^2-cyclohexadienyl) as a triflate salt, **1A** (0.81mg, 0.11 mmol, 64%). In an oven dried test tube charged with a stir pea, **1A** (0.80 g, 0.11 mmol) was dissolved in 5 mL dried THF. At room temperature, MeMgCl (3.0 M in THF, 0.07mL, 0.21 mmol) was added to the solution with a syringe. 1mL of H_2O was added to quench the reaction 10 minutes later. The solution was diluted with 10mL of DCM and extracted 3x with 10mL H_2O. The organic layer was collected, dried with Na_2SO_4, filtered over a 30mL fritted disc, and rinsed with 5mL DCM. The remaining solution was reduced to dryness, redissolved in minimal DCM and precipitated into 100mL hexanes. The precipitate was collected over a 15mL fritted disc and discarded. The remaining filtrate was reduced to dryness to yield yellow/white powder **9** in a 5 : 1 ratio (0.045 g, 69%). **^{1}H NMR (800 MHz, CD_2Cl_2, δ, 25 °C):** 8.02 (bs, 2H, Pz3A/Pz3B), 7.74 (d, *J* = 2.45 Hz, 1H, Pz5A or B), 7.72 (d, *J* = 2.21 Hz, 1H, Pz5C), 7.67 (d, *J* = 2.45 Hz, 1H, Pz5A or B), 7.32 (d, *J* = 2.10 Hz, 1H, Pz3C), 6.42 (ddd, *J* = 2.97, 5.09, 8.63 Hz, 1H, H6), 6.30 (t, *J* = 2.15 Hz, 1H, Pz4A or B), 6.24 (t, *J* = 2.10 Hz, 1H, Pz4A or B), 6.21 (t, *J* = 2.30 Hz, 1H, Pz4C), 4.98

(ddd, J = 2.03, 6.30, 8.99 Hz, 1H, H5), 2.86 (ddd, J = 4.94, 10.23, 14.09 Hz, 1H, H1), 2.83 – 2.77 (m, 1H, H3), 2.77 – 2.67 (m, 1H, H4), 1.73 (dd, J = 6.46, 16.34 Hz, 1H, H4), 1.25 (d, J = 8.44 Hz, 9H, PMe_3), 1.21 (d, J = 6.82 Hz, 3H, H7), 1.09 (d, J = 10.32 Hz, 1H, H2). **^{13}C NMR (201 MHz, CD_2Cl_2, δ, 25 °C):** 143.4 (d, J_{PC} = 1.73 Hz, 1C, Pz3A), 142.1 (1C, Pz3B), 140.7 (1C, Pz3C), 136.8 (1C, Pz5A or B), 136.2 (1C, Pz5C), 135.9 (1C, Pz5A or B), 130.9 (d, J_{PC} = 3.27 Hz, 1C, C6), 117.4 (1C, C5), 106.5 - 105.9 (3C, Pz4A/Pz4B/Pz4C), 62.8 (1C, C2), 49.6 (d, J_{PC} = 9.61 Hz, 1C, C1), 30.9 - 30.6 (2C, C3/C4), 26.7 (1C, C7), 14.1 (d, J_{PC} = 27.60 Hz, 3C, PMe_3). ESI-HRMS (m/z): $[M+H]^+$ calculated for 598.1847; found 598.1848.

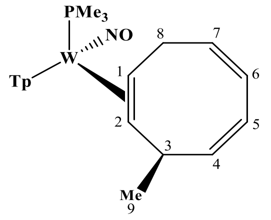

Compound 10. In an oven dried test tube charged with a stir pea, **8** (0.591 g, 0.78 mmol) was dissolved in 50 mL dried THF. At room temperature, with a syringe, MeMgCl (3.0 M in THF, 12 mL, 1.68 mmol) was added to the solution. After 30 minutes of stirring, 2mL of H_2O was added to quench the reaction. The solution was diluted with 100mL of $CDCl_3$ and extracted 3x with 100mL H_2O. The organic layer was collected, dried with Na_2SO_4 and filtered over a 60mL fritted disc and rinsed with 50mL $CDCl_3$. The remaining solution was reduced to dryness, redissolved in minimal MeCN and precipitated into 300mL Et_2O. The precipitate was collected over a 30mL fritted disc and discarded. The remaining ether filtrate was reduced to dryness to yield white powder **10** in a 20 : 1 ratio (0.326 g, 65%). **^{1}H NMR (800 MHz, CD_3CN, δ, 25 °C):** 7.97 (d, J = 2.12 Hz, 1H, Pz3A), 7.95 (d, J = 1.86 Hz, 1H, Pz3B), 7.82 (d, J = 2.18 Hz, 1H, Pz5C), 7.81 (d, J = 2.14 Hz, 1H, Pz5B), 7.78 (d, J = 2.63 Hz, 1H, Pz5A), 7.32 (d, J = 2.06 Hz, 1H, Pz3C), 6.31 (t, J = 2.33 Hz, 1H, Pz4B), 6.27 (t, J = 2.01 Hz, 1H, Pz4A), 6.26 (t, J = 2.12 Hz, 1H, Pz4C), 6.05 (dd, J = 3.06, 10.36 Hz, 1H, H6), 5.87 (q, J = 7.71 Hz ,1H, H7), 5.81 (dd, J = 7.71, 11.00 Hz, 1H, H5), 5.79 (dd, J = 2.97, 10.92 Hz, 1H, H4), 3.64 – 3.59 (m, 1H, H3), 2.78 – 2.70 (m, 1H, H1), 1.17 (d, J = 8.26 Hz, 9H, PMe_3), 1.05 (d, J = 6.97 Hz, 3H, H9), 0.73 (dd, J = 4.18, 11.54 Hz, 1H, H2. **^{13}C NMR (201 MHz, CD_3CN, δ, 25 °C):** 144.1 (1C, Pz3B), 144.0 (1C, Pz3A), 141.7 (1C, Pz3C), 140.9 (1C, C4), 137.6 – 137.5 (2C, Pz5A/P5C), 137.0 (2C, Pz5B/C7), 129.3 (1C, C6), 126.3 (1C, C5), 107.2 – 106.8 (3C, Pz4B/Pz4A/Pz4C), 62.4 (1C, C2), 53.8 (d, J_{PC} = 11.62 Hz, 1C, C1), 37.8 (1C, C3), 34.1 (d, J_{PC} = 2.70 Hz, 1C, C8), 27.3 (1C, C9), 13.26 (d, J_{PC} = 27.43 Hz 1C, PMe_3). Composition of **10** confirmed by single crystal X-ray diffraction.

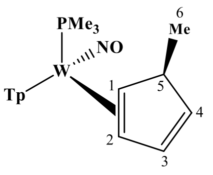

11D

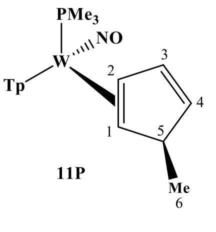

11P

Compounds 11D and 11P. In an oven dried test tube charged with a stir pea, **5** (0.104 g, 0.18 mmol) was dissolved in 5 mL dried THF. In another oven dried test tube charged with a stir pea, DDQ (0.042 g, 0.19 mmol) was dissolved in 2 mL dried THF. Both test tubes were chilled to -15°C for 15 minutes. The solution of DDQ was transferred into the test tube containing compound **5**. To the chilled solution MeMgCl (3.0 M in THF, 0.20 mL, 0.28 mmol) was added via syringe and was stirred at -15°C for 30 minutes. The crude

reaction mixture was then diluted with DCM and extracted 3x with 10 mL H_2O. The reaction was then dried with Na_2SO_4 and filtered to remove drying agent. Afterwards the reaction was reduced to dryness in vacuo in a 125 mL filter flask. The remaining product on the flask was redissolved in minimal DCM, precipitated into 50mL hexanes and filtered over a 15mL fritted disc. The precipitate was discarded. The hexanes filtrate was reduced to dryness and weighed. The white solid **11D and 11P** was collected in a 3 : 2 ratio (0.082 g, 77%). **11D**: **^{1}H NMR (800 MHz, CD_3CN, δ, 25 °C):** 8.14 (d, *J* = 2.0 Hz, 1H, Pz), 8.01 (d, *J* = 2.0 Hz, 1H, Pz), 7.87 (d, *J* = 2.4 Hz, 1H, Pz), 7.80 (d, *J* = 2.3 Hz, 1H, Pz), 7.73 (d, *J* = 2.3 Hz, 1H, Pz), 7.45 (d, *J* = 2.2 Hz, 1H), 6.47 (dt, *J* = 5.1, 1.9 Hz, 1H, H3), 6.37 (t, *J* = 2.3 Hz, 1H, Pz), 6.29 (t, *J* = 2.2 Hz, 1H, Pz), 6.23 (t, *J* = 2.2 Hz, 1H, Pz), 5.01 (dd, *J* = 5.1, 2.4 Hz, 1H, H4), 3.54 (m, 1H, H5), 3.09 (dd, *J* = 13.4, 7.2 Hz, 1H, H1), 2.08 (m, 1H, H2), 1.29 (d, *J* = 7.1 Hz, 3H, H6), 1.22 (d, *J* = 8.3 Hz, 9H, PMe_3). **^{13}C NMR (201 MHz, CD_3CN, δ, 25 °C):** 145.0 (1C, Pz), 144.6 (1C, Pz), 142.2 (1C, Pz), 137.7 (1C, Pz), 137.6 (1C, Pz), 137.2 (1C, C3), 136.3 (1C, Pz), 128.3 (1C, H4), 107.4 (1C, Pz), 107.0 (1C, Pz), 106.8 (1C, Pz), 68.4 (d, *J* = 12.4 Hz, 1C, C1), 64.8 (1C, C2), 50.1 (1C, C5), 25.9 (1C, C6), 13.5 (d, *J* = 27.9 Hz, 3C, PMe_3). **11P**: **^{1}H NMR (800 MHz, CD_3CN, δ, 25 °C):** 8.34 (d, *J* = 2.0 Hz, 1H, Pz), 8.01 (d, *J* = 2.0 Hz, 1H, Pz), 7.87 (d, *J* = 2.4 Hz, 1H, Pz) 7.80 (d, *J* = 2.3 Hz, 1H, Pz), 7.76 (d, *J* = 2.3 Hz, 1H, Pz), 7.40 (d, *J* = 2.2 Hz, 1H, Pz), 6.37 (t, *J* = 2.3 Hz, 1H, Pz), 6.30 (m, 2H, H3 & Pz), 6.22 (t, *J* = 2.2 Hz, 1H, Pz), 5.05 (dd, *J* = 5.1, 2.4 Hz, 1H, H4), 3.90 (dddd, *J* = 9.1, 7.1, 5.5, 1.9 Hz, 1H, H5), 3.60 (ddt, *J* = 12.4, 7.2, 2.0 Hz, 1H, H2), 1.61 (dd, *J* = 7.2, 2.8 Hz, 1H, H1), 1.24 (d, *J* = 8.4 Hz, 9H, PMe_3), 1.15 (d, *J* = 7.2 Hz, 3H, H6). **^{13}C NMR (201 MHz, CD_3CN, δ, 25 °C):** 145.0 (1C, Pz), 142.4 (1C, Pz), 141.4 (1C, Pz), 137.2 (1C, Pz), 137.0 (1C, Pz), 136.5 (1C, Pz), 136.3 (d, J_{PC}= 2.4 Hz, 1C, C3), 128.9 (1C, C4), 107.3 (1C, Pz), 106.7 (1C, Pz), 106.7 (1C, Pz), 67.2 (1C, C1), 66.5 (d, J_{PC} = 10.8 Hz, 1C, C2), 50.5 (1C, C5), 26.7 (1C, C6), 14.2 (d, J_{PC} = 28.6 Hz, 3C, PMe_3). APCI-HRMS (m/z): $[M+H]^+$ calculated for 584.1690; found 584.1690.

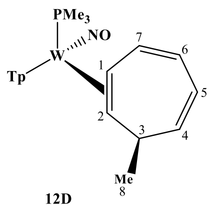

12D

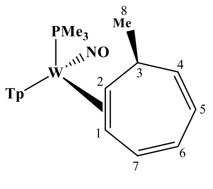

12P

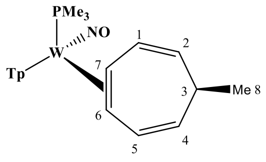

12M

Compound 12. Compound **4** (0.29g, 0.391 mmol) and methyllithium (0.98 mL, 1.6M, diethyl ether) were added to separate test tubes and treated with 1.5 mL anhydrous tetrahydrofuran, respectively. The test tube containing **4** was charged with a stir pea, and both test tubes were capped and allowed to chill at -30 °C for 10 minutes. After chilling, the methyllithium solution was pipetted into the test tube containing **4**, and the reaction mixture was allowed to stir for 1.5h. The reaction was monitored by ^{31}P NMR and determined to be at completion with the disappearance of a single ^{31}P peak corresponding to **4** and the emergence of two ^{31}P peaks in a 5:2 ratio. The reaction mixture was then added to a 125 mL separatory funnel and diluted with 25 mL of dichloromethane (DCM). The reaction mixture was then extracted 3 times with 25 mL of a saturated bicarbonate solution. The aqueous layer was discarded, and the organic layer was dried with magnesium sulfate. The magnesium sulfate was filtered through a 30 mL medium porosity fritted funnel, and the dried organic layer was evaporated to a thin film and was concentrated *in vacuo*. The thin film was dissolved in minimal DCM and pipetted into 100 mL of stirring pentane, precipitating a tan solid. The tan solid was separated from the pentane mixture with a 15 mL fine porosity fritted funnel, and the pentane mixture was concentrated *in vacuo*, resulting in a thin white film. The film was dissolved in 1 mL of DCM and loaded onto an 8-gram basic alumina column and purified via Combiflash column chromatography (40:60, hexanes/ethyl acetate). The fraction containing compound **12** was concentrated *in vacuo*. The resulting film was dried overnight in a vacuum oven at 50 °C to give a yellow oil, **12** (19 mg, 8) Coordination Diastereomer ratio after purification: 5:3:2.5, **12M**:**12D**:**12P**. Composition of **12D** was confirmed via single crystal x-ray diffraction.

Where possible, ^{1}H proton data was assigned, however due to the extent of overlapping ^{1}H and ^{13}C signals only a partial characterization can be done of **12** unambiguously. **12D:** ^{1}H NMR (600 MHz, CD_3CN) δ: 6.71 (dt, *J* = 11.5, 6.9 Hz, 2H), 5.77 – 5.73 (m, 1H), 5.73 – 5.68 (m, 1H), 5.01 (ddd, *J* = 11.5, 6.0, 1.5 Hz, 1H), 3.02 – 2.94 (m, 1H), 2.90 – 2.83 (m, 1H), 1.74 (d, *J* = 1.2 Hz, 1H), 1.42 – 1.40 (m, 3H), 1.22 (d, *J* = 8.4 Hz, 9H). ^{13}C NMR (151 MHz, CD_3CN) δ: 129.0, 82.8, 53.9 (d, *J* = 7.5 Hz), 37.4, 24.6, 13.8 (d, *J* = 28.2 Hz). **12P**: ^{1}H NMR (600 MHz, CD_3CN) δ 6.75 – 6.64 (m, 1H), 5.59 – 5.53 (m, 1H), 5.18 (ddd, *J* = 11.3, 6.4, 0.8 Hz, 1H), 3.40 – 3.28* (m, 1H), 1.58 (d, *J* = 6.7 Hz, 3H), 1.44 – 1.39 (m, 1H), 1.17 (d, *J* = 8.3 Hz, 8H). ^{13}C NMR (151 MHz, CD_3CN) δ 139.9 (d, *J* = 3.1 Hz), 128.1, 83.3 (d, *J* = 13.0 Hz), 54.5, 40.3 (d, *J* = 2.8 Hz), 25.6, 13.3 (d, *J* = 28.3 Hz). **12M:** ^{1}H NMR (600 MHz, CD_3CN) δ: 6.60 – 6.54 (m, 1H), 6.48 (ddd, J = 11.8, 7.6, 2.7 Hz, 1H), 6.36 (t, J = 2.2 Hz, 1H), 6.24 (t, J = 2.2 Hz, 1H), 4.93 (ddd, J = 11.8, 2.7, 1.8 Hz, 1H), 4.88 – 4.82 (m, 1H), 4.36 – 4.29 (m, 1H), 2.80 (ddd, J = 11.6, 10.6, 7.0 Hz, 1H), 1.34 – 1.30 (m, 1H), 1.24 (d, J = 8.6 Hz, 8H), 0.98 (d, J = 7.6 Hz, 3H). ^{13}C NMR (151 MHz, CD_3CN) δ: 137.3, 130.7, 129.9, 56.0 (d, *J* = 8.6 Hz), 55.2, 33.9, 25.0, 14.0 (d, *J* = 28.7 Hz) **APCI-HRMS** (m/z): [M+H]+ calculated for $C_{20}H_{30}BN_7OPW^+$, 610.1846; found 610.1840.

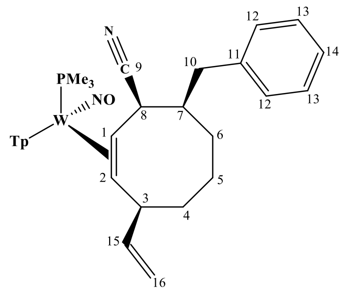

Compound 13. In an oven dried test tube charged with a stir pea, **8** (0.40 g, 0.53 mmol) was dissolved in 5 mL dried THF. To this solution, BnMgCl (1.0 M in Et_2O, 0.80 mL, 0.79 mmol) was added via syringe and the reaction stirred for 30 minutes. The crude reaction mixture was then diluted with DCM and extracted 3x with 10 mL H_2O. The reaction was then dried with Na_2SO_4 and filtered to remove drying agent. Afterwards, the reaction was reduced to dryness in vacuo in a 125 mL filter flask. The remaining product on the flask was redissolved in minimal DCM, precipitated into 50mL hexanes and filtered over a 15mL fritted disc. The precipitate was discarded. The hexanes filtrate was reduced to dryness and weighed. The white solid, a benzyl-substituted cyclooctatriene complex analogous to **10**, was collected (233 mg, 63.1%). Next, the resulting triene complex (286 mg, 0.41 mmol) was dissolved in 3 mL DCM, protonated with HOTf (70.4 mg, 0.47 mmol), and left to stir for 12 hours. Afterwards, 125 mL hexanes was added, precipitating out a whitish tan solid. The precipitated dienyl complex was collected as a triflate salt on a 15 mL fine porosity fritted funnel (314 mg, 93%). Then, in a test tube charged with a stir pea, the dienyl complex (300 mg, 0.35 mmol) was dissolved in 5 mL MeCN. To this solution, NaCN (173 mg, 3.53 mmol) was added with a few drops of DMSO. This reaction mixture was stirred for 12 hours. The crude reaction mixture was then diluted with DCM and extracted 3x with 10 mL H_2O. The reaction was then dried with Na_2SO_4 and filtered to remove drying agent. Afterwards the reaction was reduced to dryness and weighed in vacuo in a 125 mL filter flask. The resulting white solid, a disubstituted diene complex, was collected (220 mg, 85.7%). Following this, the diene complex (193 mg, 0.27 mmol) was dissolved in 3 mL DCM, protonated with HOTf (44 mg, 0.29 mmol), and stirred for 5 minutes. Afterwards, 150 mL hexanes was added, precipitating out a white solid. The precipitated allyl complex was collected as a triflate salt on a 15 mL porosity fritted funnel (224 mg, 96%). Next, in a test tube charged with a stir pea, the allyl complex (125 mg, 0.14 mmol) was dissolved in 5 mL dried THF. To the stirring solution, C_2H_3MgBr (1.0 M in THF, 14% wt., 3.06 mL, 0.42 mmol) was added via syringe. After reacting for 30 minutes, the crude reaction mixture was then diluted with DCM and extracted 3x with 10 mL H_2O. The reaction was then dried with Na_2SO_4 and filtered to remove drying agent. Afterwards the reaction was reduced to dryness in vacuo in a

125 mL filter flask. The remaining product on the flask was redissolved in minimal DCM, precipitated into 50mL hexanes and filtered over a 15mL fritted disc. The precipitate was discarded. The hexanes filtrate was reduced to dryness and weighed. The resulting white solid, the trisubstituted cyclooctene complex **13**, was collected (89 mg, 83%). **^{1}H NMR (600 MHz, CD_2Cl_2, δ, 25 °C):** 7.93 (d, *J* = 2.1 Hz, 1H, Pz), 7.82 (d, *J* = 2.0 Hz, 1H, Pz), 7.80 (d, *J* = 2.2 Hz, 1H, Pz), 7.70 (d, *J* = 2.4 Hz, 1H, Pz), 7.64 (d, *J* = 2.4 Hz, 1H, Pz), 7.33 – 7.28 (m, 4H, H12 & H13), 7.27 (d, *J* = 2.2 Hz, 1H, Pz), 7.20 (t, *J* = 6.1, 1H, H14), 6.32 (t, *J* = 2.2 Hz, 1H, Pz), 6.26 (t, *J* = 2.2 Hz, 1H, Pz), 6.08 (t, *J* = 2.2 Hz, 1H, Pz), 5.29 (m, 1H, H15), 4.22 (d, *J* = 17.5 Hz, 1H, H16), 4.17 (m, 1H, H16), 3.74 (m, 1H, H8), 3.37 (m, 1H, H10), 2.98 (m, 2H, H1 & H3), 2.75 (appt, *J* = 12.2 Hz, 1H, H10), 2.34 (m, 1H, H7), 1.94 (m, 1H, H5), 1.85 (m, 1H, H4), 1.65 – 1.52 (m, 2H, H6), 1.25 (m, 1H, H5), 1.13 (d, *J* = 8.1 Hz, 10H, PMe_3 & H4), 0.93 (appt, *J* = 10.3 Hz, 1H, H2). **^{13}C NMR (201 MHz, CD_2Cl_2, δ, 25 °C):** 148.1 (1C, C15), 146.5 (1C, Pz), 143.1 (1C, Pz), 141.5 (1C, C11), 139.9 (1C, Pz), 137.3 (1C, Pz), 136.7 (1C, Pz), 136.5 (1C, Pz), 129.8 (2C, C12 or C13), 128.6 (2C, C12 or C13), 127.7, (1C, C9), 126.3 (1C, C14), 109.2 (1C, C16), 106.8 (1C, Pz), 106.5 (1C, Pz), 106.0 (1C, Pz), 65.5 (1C, C2), 47.4 (1C, C7), 45.9 (1C, C4), 44.2 (1C, C1), 40.1 (1C, C8), 39.4 (1C, C10), 29.9 (1C, C6), 29.5 (1C, C3), 24.2 (d, *J* = 23.8 Hz, 1C, C5), 14.2 (d, *J* = 27.2 Hz, 3C, PMe_3). Composition of **13** confirmed by single crystal X-ray diffraction.

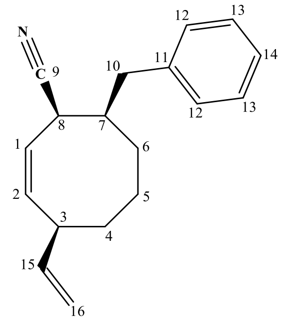

Compound 14. In an oven dried test tube charged with a stir pea, **13** (38 mg, 0.050 mmol) was dissolved in 1 mL acetone. In a separate test tube, cerium(IV) ammonium nitrate (33 mg, 0.060 mmol) was dissolved in 1 mL acetone. The solution of cerium(IV) ammonium nitrate was pipetted into the solution of **13**, and the reaction was stirred for 5 minutes. The crude reaction mixture was then diluted with DCM and extracted 3x with 10 mL H_2O. The reaction was then dried with Na_2SO_4 and filtered to remove drying agent. Afterwards the reaction was reduced to dryness in vacuo in a 125 mL filter flask. Minimal DCM was used to redissolve the product, and 50 mL hexanes was used to precipitate out an orange solid. The solid was discarded on a fritted funnel, and the clear filtrate was transferred into a round-bottom flask and rotovapped to dryness. The resulting white solid **14** was redissolved in MeCN and transferred into a vial, which was reduced to dryness and weighed (6.0 mg, 47%). **^{1}H NMR (800 MHz, CD_3CN, δ, 25 °C):** 7.29 (t, *J* = 7.7 Hz, 2H, H13), 7.21 (m, 1H, H14), 7.20 (m, 2H, H12), 5.87 (ddd, *J* = 17.3, 10.3, 6.9 Hz, 1H, H15), 5.73 (m, 1H, H2), 5.60 (t, *J* = 9.9 Hz, 1H, H1), 5.07 (dd, *J* = 17.3, 1.4 Hz, 1H, H16), 4.99 (m, 1H, H16), 4.03 (m, 1H, H8), 3.12 (m, 1H, H3), 3.05 (dt, *J* = 13.5, 3.0 Hz, 1H, H10), 2.51 (t, *J* = 12.6 Hz, 1H, H10), 2.34 (dp, *J* = 12.7, 4.4 Hz, 1H, H7), 1.66 (m, 2H, H4 & H5), 1.47 (ddd, *J* = 20.3, 9.9, 4.9 Hz, 1H, H6), 1.37 – 1.19 (m, 3H, H4, H5, & H6). **^{13}C NMR (201 MHz, CD_3CN, δ, 25 °C):** 143.13 (1C, C15), 141.10 (1C, C11), 138.08 (1C, C2), 130.01 (2C, C12), 129.32 (2C, C13), 127.11 (1C, C14), 122.53 (1C, C1), 114.23 (1C, C16), 104.97 (1C, C9), 45.64 (1C, C7), 41.96 (1C, C3), 37.44 (2C, C4 & C5), 37.06 (1C, C10), 34.22 (1C, C8), 29.55 (1C, C6). APCI-HRMS (m/z): $[M+H]^+$ calculated for 252.1747; found 252.1733.

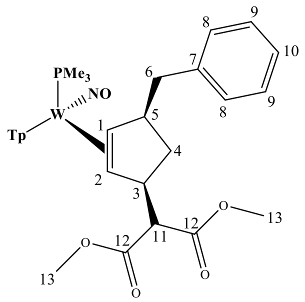

Compound 15. In an oven dried test tube charged with a stir pea, **3[DDQ]** (0.100 g, 0.125 mmol) was dissolved in 3 mL dried THF and chilled to -60 °C. To this solution, BnMgCl (1.0 M in Et_2O, 0.63 mL, 0.62 mmol) was added via syringe and the reaction stirred for 5 hours at -60 °C. The crude reaction mixture was then diluted with DCM and extracted 3x with 10 mL H_2O. The reaction was then dried with Na_2SO_4 and filtered to remove drying agent. Afterwards, the reaction was reduced to dryness in vacuo in a 125 mL filter flask. The remaining product on the flask was redissolved in minimal DCM, precipitated into 50mL hexanes and filtered over a 15mL fritted disc. The precipitate was discarded. The hexanes filtrate was reduced to dryness and weighed. The white solid, a benzyl-substituted cyclopentadiene complex analogous to **11**, was collected (67 mg, 82%). Next, the resulting diene complex (67 mg, 0.101 mmol) was dissolved in 3 mL MeCN, chilled to -10 °C, protonated with HOTf (20 mg, 0.13 mmol), and left to stir for 30 minutes. Afterwards, the reaction mixture was reduced to dryness in vacuo in a 125 mL filter flask and redissolved in minimal DCM. The redissolved reaction was pipetted into 100 mL stirring hexanes, precipitating out a whitish tan solid. The precipitated cyclopentenyl cationic complex was collected as a triflate salt on a 15 mL fine porosity fritted funnel (64 mg, 78%). Then, in an oven dried test tube charged with a stir pea, the cyclopentenyl cationic complex (64 mg, 0.079 mmol) was dissolved in 3 mL dried THF and chilled to -30 °C. To this solution, LiDiMM (40 mg, 0.29 mmol) in minimal dried THF was pipetted in and the reaction stirred for 10 minutes at -30 °C. The crude reaction mixture was then diluted with DCM and extracted 3x with 10 mL H_2O. The reaction was then dried with Na_2SO_4 and filtered to remove drying agent. Afterwards, the reaction was reduced to dryness in vacuo in a 125 mL filter flask. The remaining product on the flask was redissolved in minimal DCM, precipitated into 50mL hexanes and filtered over a 15mL fritted disc. The precipitate was discarded. The hexanes filtrate was reduced to dryness and weighed. The disubstituted complex **15** (45 mg, 72%) was collected in a 7:4:1 mixture of products and was carried on without any attempt to separate the isomers. While NMR characterization was not possible due to an excess of overlapping peaks, the major products are diastereomers of 3,5-disubstituted cyclopentene complex while the minor isomer is suspected to be 3,4-disubstituted cyclopentene complex. APCI-HRMS (m/z): $[M+H]^+$ calculated for 792.2425; found 792.2426.

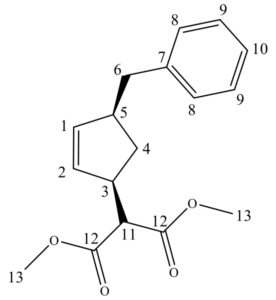

Compound 16. In an oven dried test tube charged with a stir pea, **15** (45 mg, 0.057 mmol) was dissolved in 2 mL acetone. In a separate test tube, cerium (IV) ammonium nitrate (44 mg, 0.080 mmol) was dissolved in 2 mL acetone. The solution of cerium (IV) ammonium nitrate was pipetted into the solution of **15**, and the reaction was stirred for 10 minutes. The crude reaction mixture was then diluted with DCM and

extracted 3x with 10 mL H_2O. The reaction was then dried with Na_2SO_4 and filtered to remove drying agent. Afterwards the reaction was reduced to dryness in vacuo in a 125 mL filter flask. Minimal DCM was used to redissolve the product, and 50 mL hexanes was used to precipitate out an orange solid. The solid was discarded on a fritted funnel, and the clear filtrate was transferred into a round-bottom flask and rotovapped to dryness. Finally, the product was run down a CombiFlash silica 5g cartridge and came off with 100% hexanes. The hexanes fraction was rotovapped to dryness and weighed, yielding **16** (7 mg, 42%). The compound was isolated in a 10:1 isomeric ratio in which the minor isomer is suspected to be a 3,4-disubstituted cyclopentene. **^{1}H NMR (800 MHz, $(CD_3)_2CO$, δ, 25 °C):** 7.28 (t, *J* = 7.4 Hz, 2H, H9), 7.21 (d, *J* = 7.5 Hz, 2H, H8), 7.18 (m, 1H, H10), 5.73 (m, 1H, H2), 5.65 (dd, *J* = 5.9, 2.1 Hz, 1H, H1), 3.69 (s, 3H, H13), 3.68 (s, 3H, H13), 3.27 (m, 2H, H3 & H11), 3.00 (m, 1H, H5), 2.72 (dd, *J* = 13.5, 7.1 Hz, 1H, H6), 2.61 (dd, *J* = 13.4, 8.0 Hz, 1H, H6), 2.23 (m, 1H, H4), 1.25 (m, 1H, H4). **^{13}C NMR (201 MHz, $(CD_3)_2CO$, δ, 25 °C):** 169.49 (2C, C12), 141.67 (1C, C7), 137.09 (1C, C2), 132.41 (1C, C1), 129.74 (2C, C8), 129.11 (2C, C9), 126.71 (1C, C10), 57.85 (1C, C11), 52.49 (2C, C13), 48.00 (1C, C5), 46.11 (1C, C3), 43.10 (1C, C6), 35.24 (1C, C4). APCI-HRMS (m/z): $[M+H]^+$ calculated for 289.1434; found 289.1436.

Figure S2: ^{1}H-NMR (CD_3CN) and ^{13}C-NMR (CD_3CN) of Compound 2

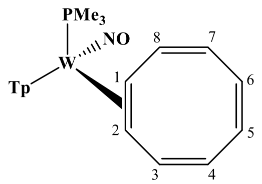

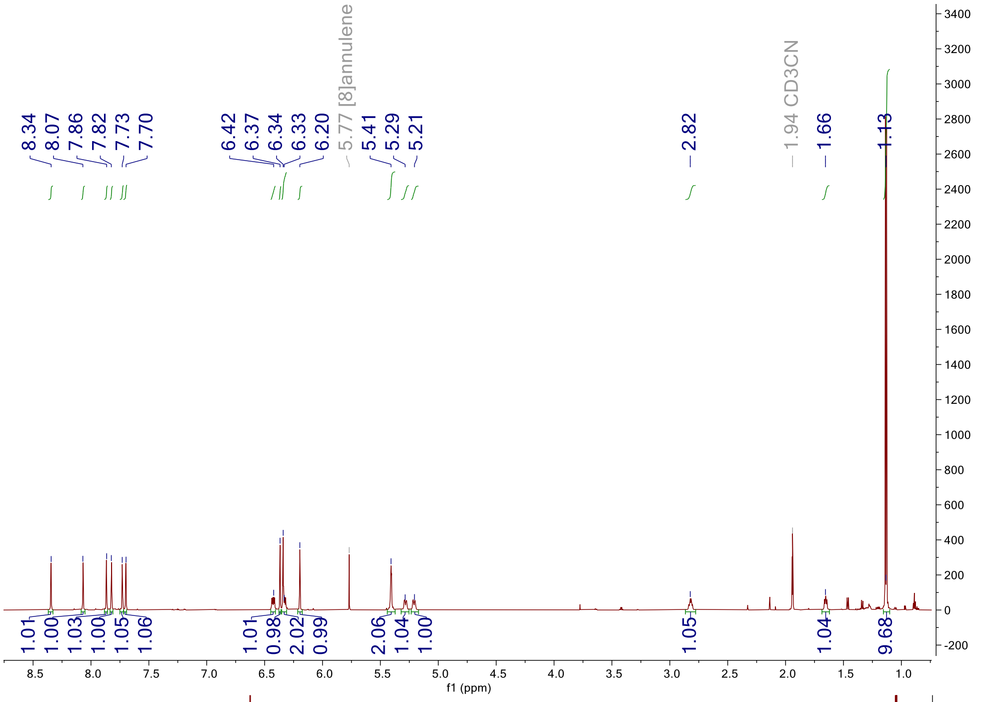

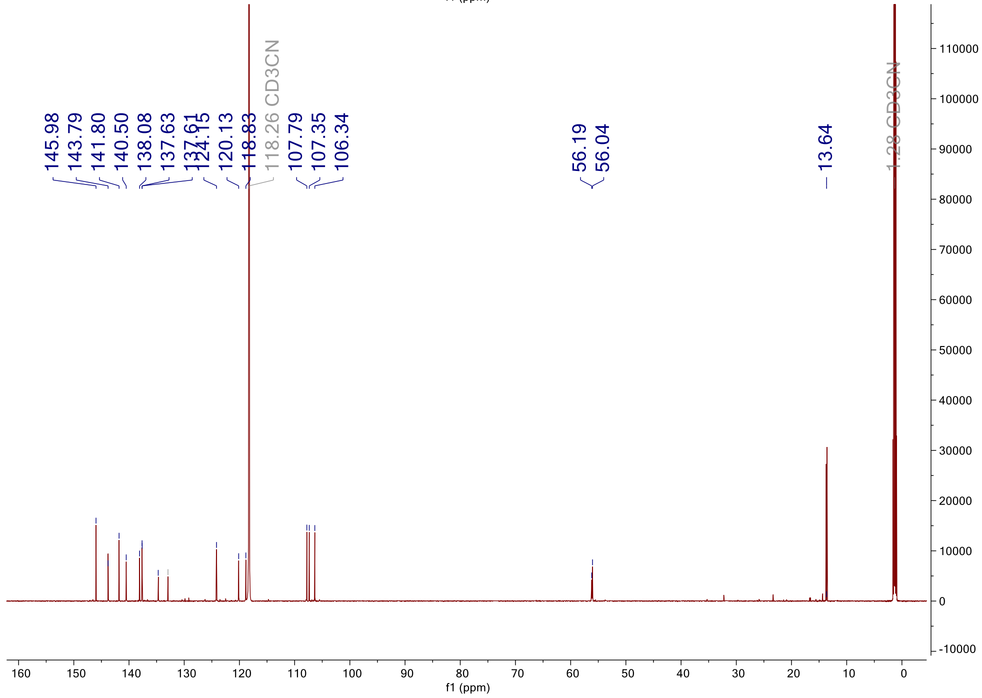

Figure S3: ^{1}H-NMR (CD_3CN) and ^{13}C-NMR (CD_3CN) of Compound 3[DDQ]

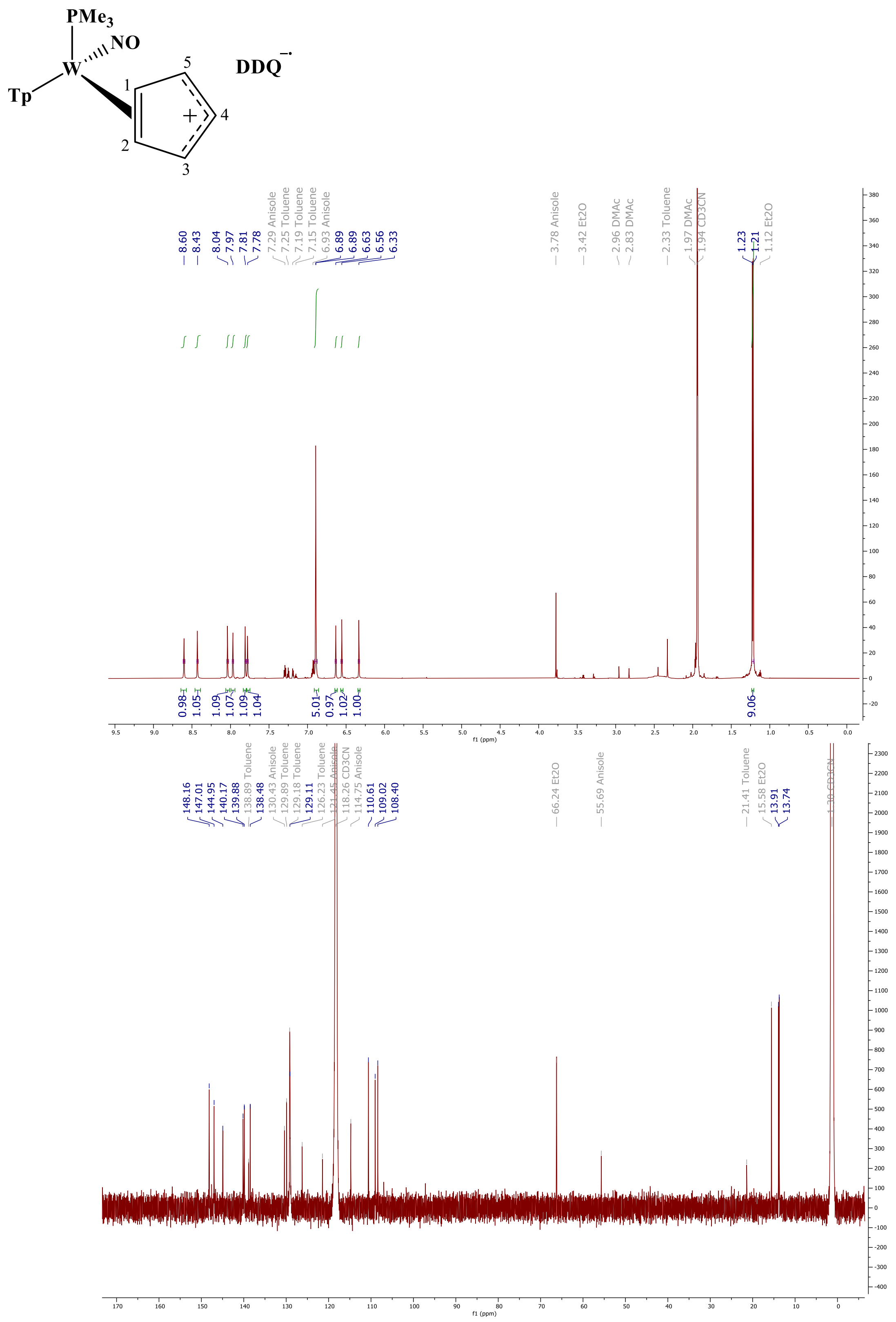

Figure S4: ^{1}H-NMR (CD_3CN) and ^{13}C-NMR (CD_3CN) of Compound 3[OTf]

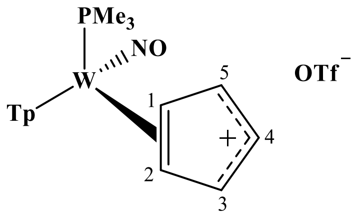

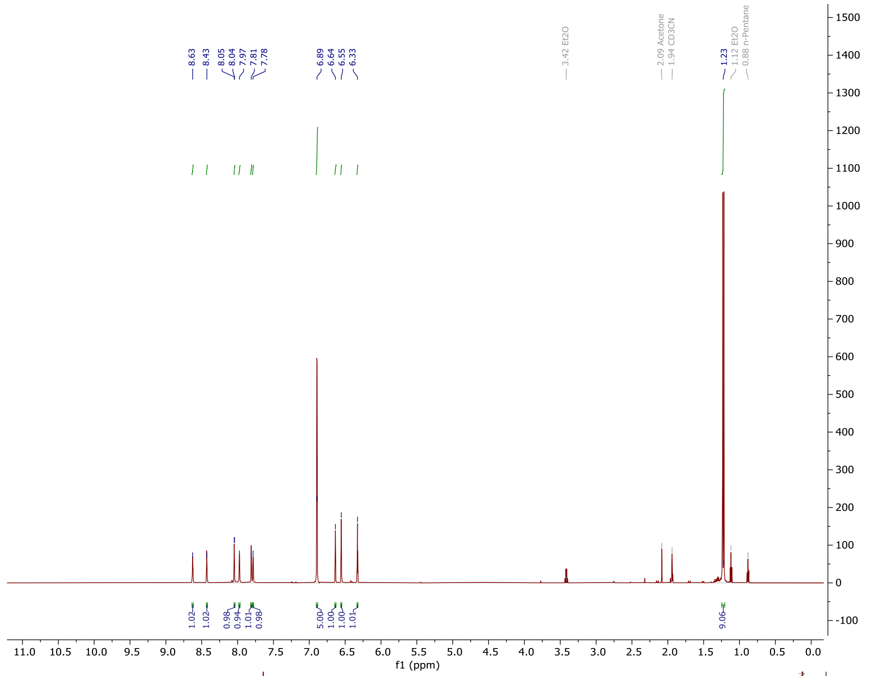

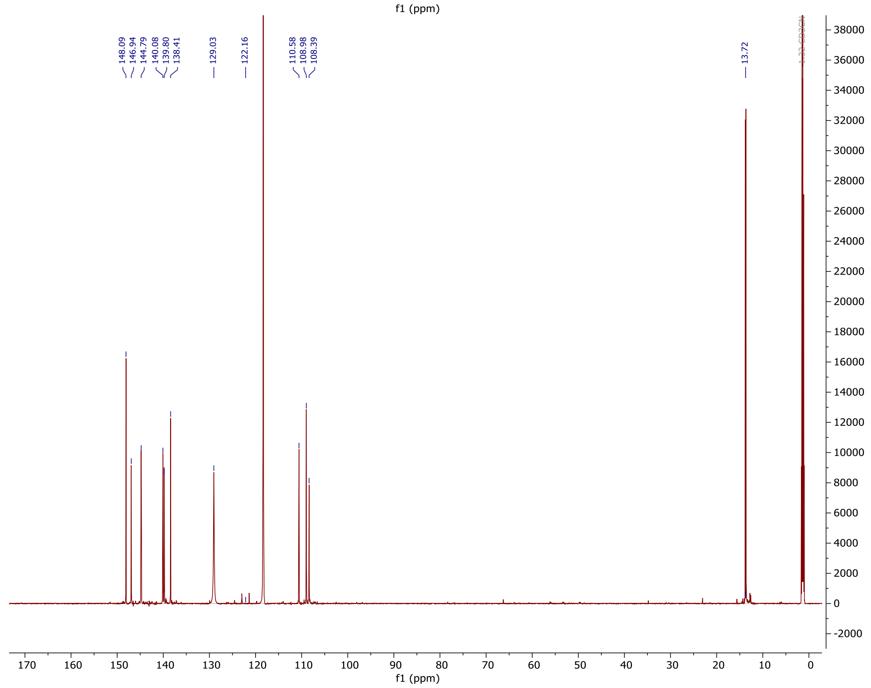

Figure S5: ^{1}H-NMR (CD_3CN) and ^{13}C-NMR (CD_3CN) of Compound 4

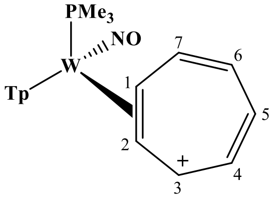

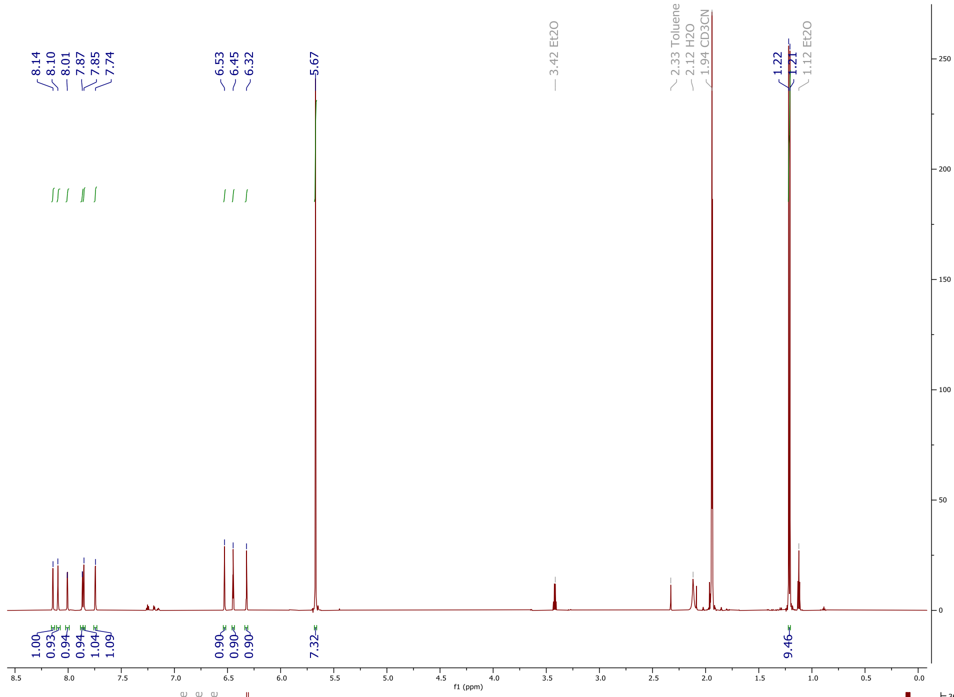

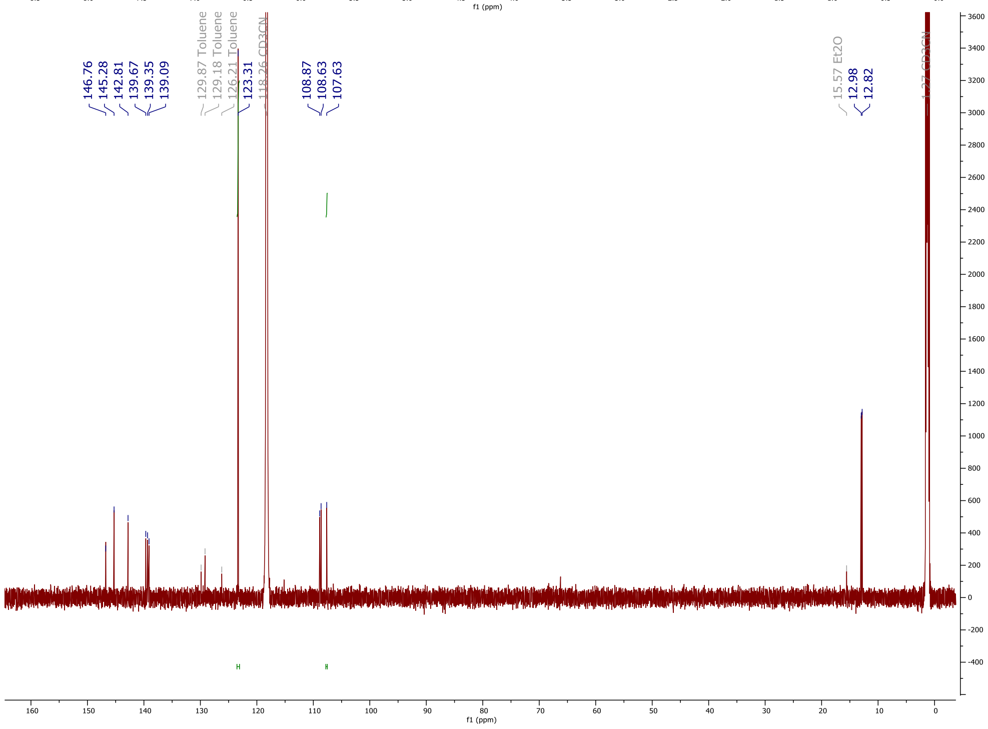

Figure S6: ^{1}H-NMR (CD_3CN) and ^{13}C-NMR (CD_3CN) of Compounds 6D & 6P

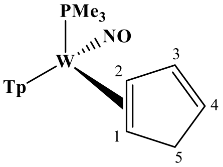

6P

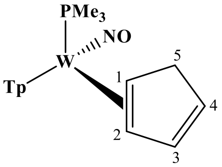

6D

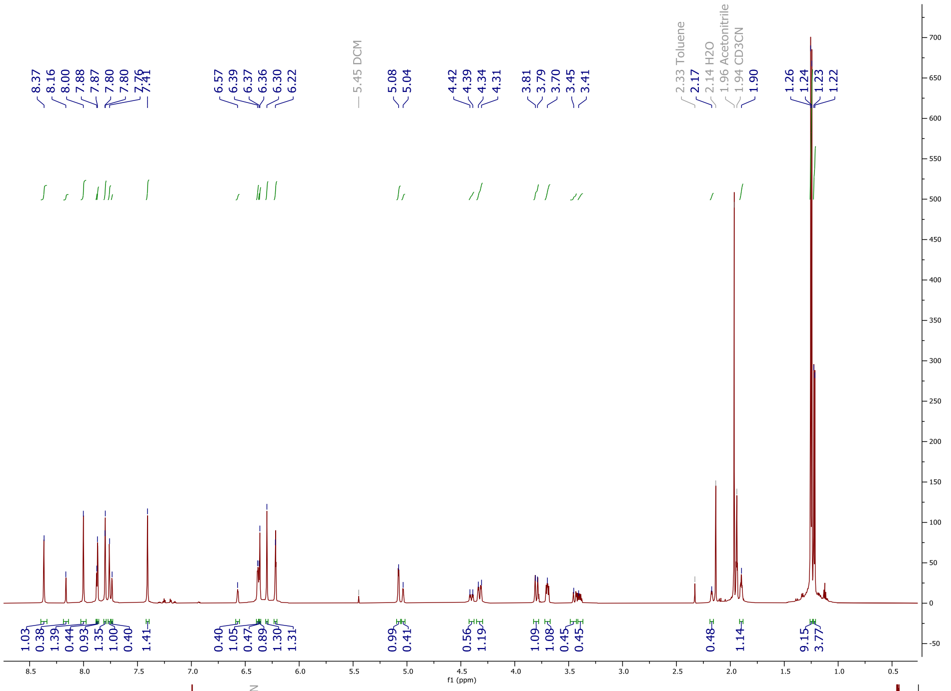

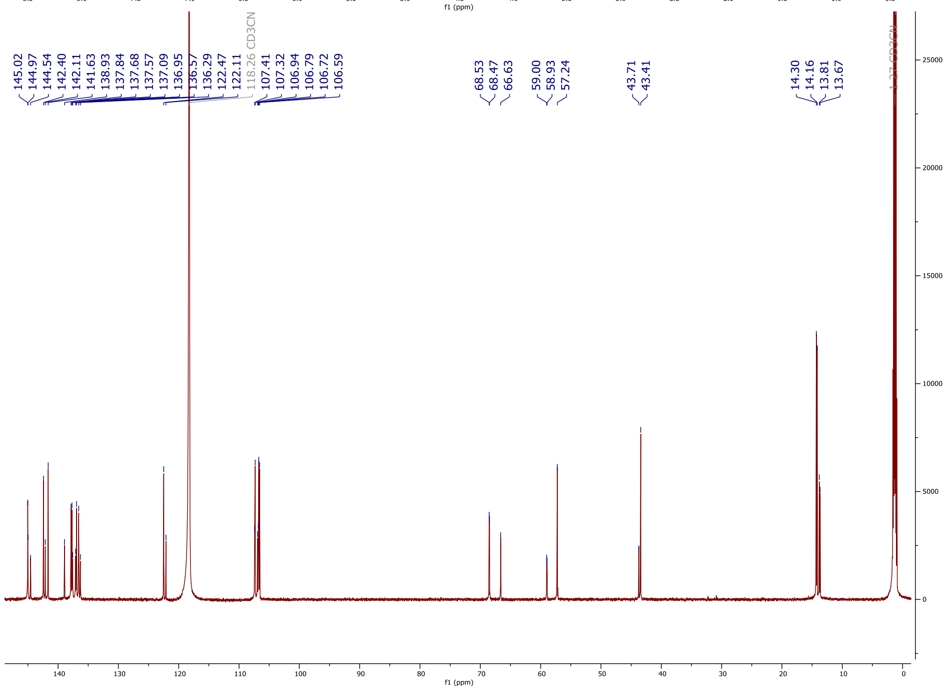

Figure S7: ^{1}H-NMR ($CDCl_3$) and ^{13}C-NMR ($CDCl_3$) of Compounds 7D, 7M, & 7P

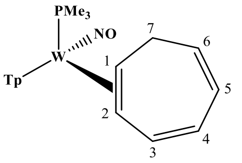

7D

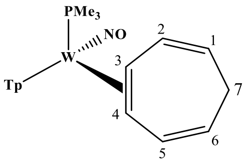

7M

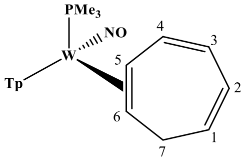

7P

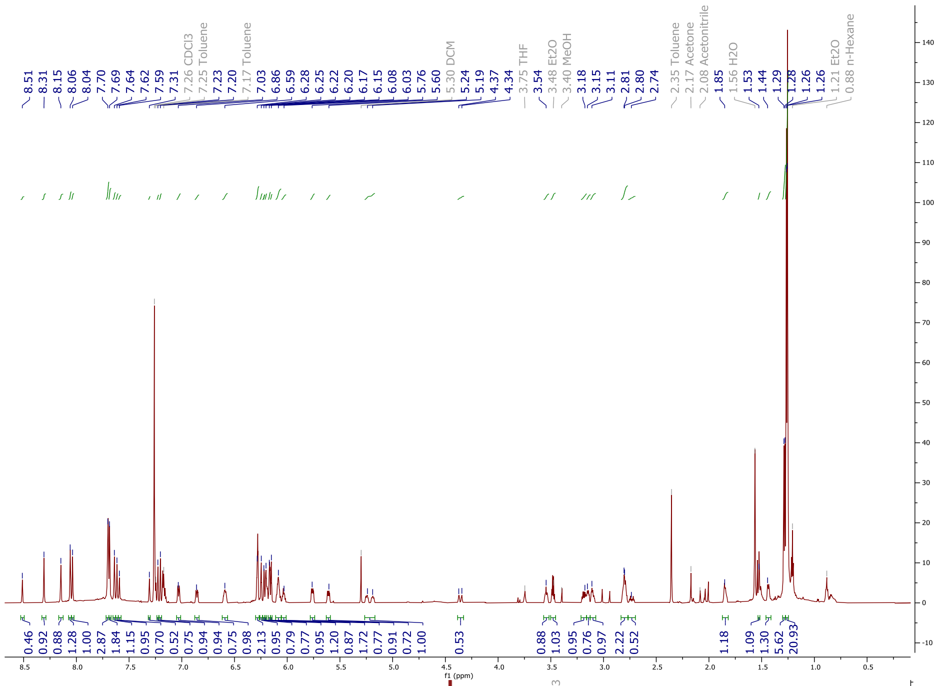

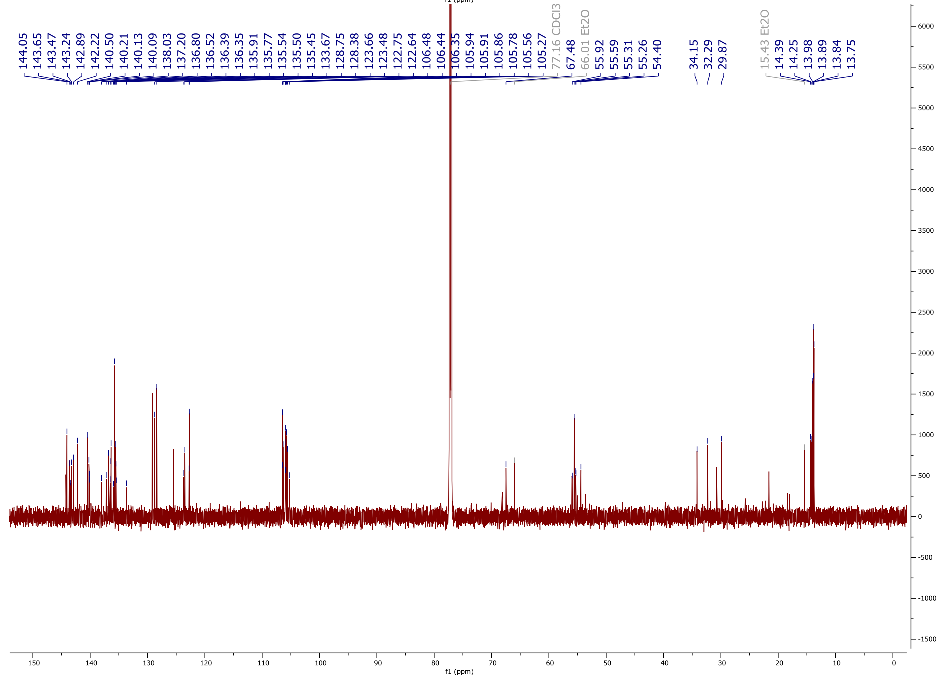

Figure S8: ^{1}H-NMR ($CDCl_3$) and ^{13}C-NMR ($CDCl_3$) of Compound 7A

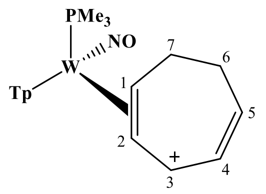

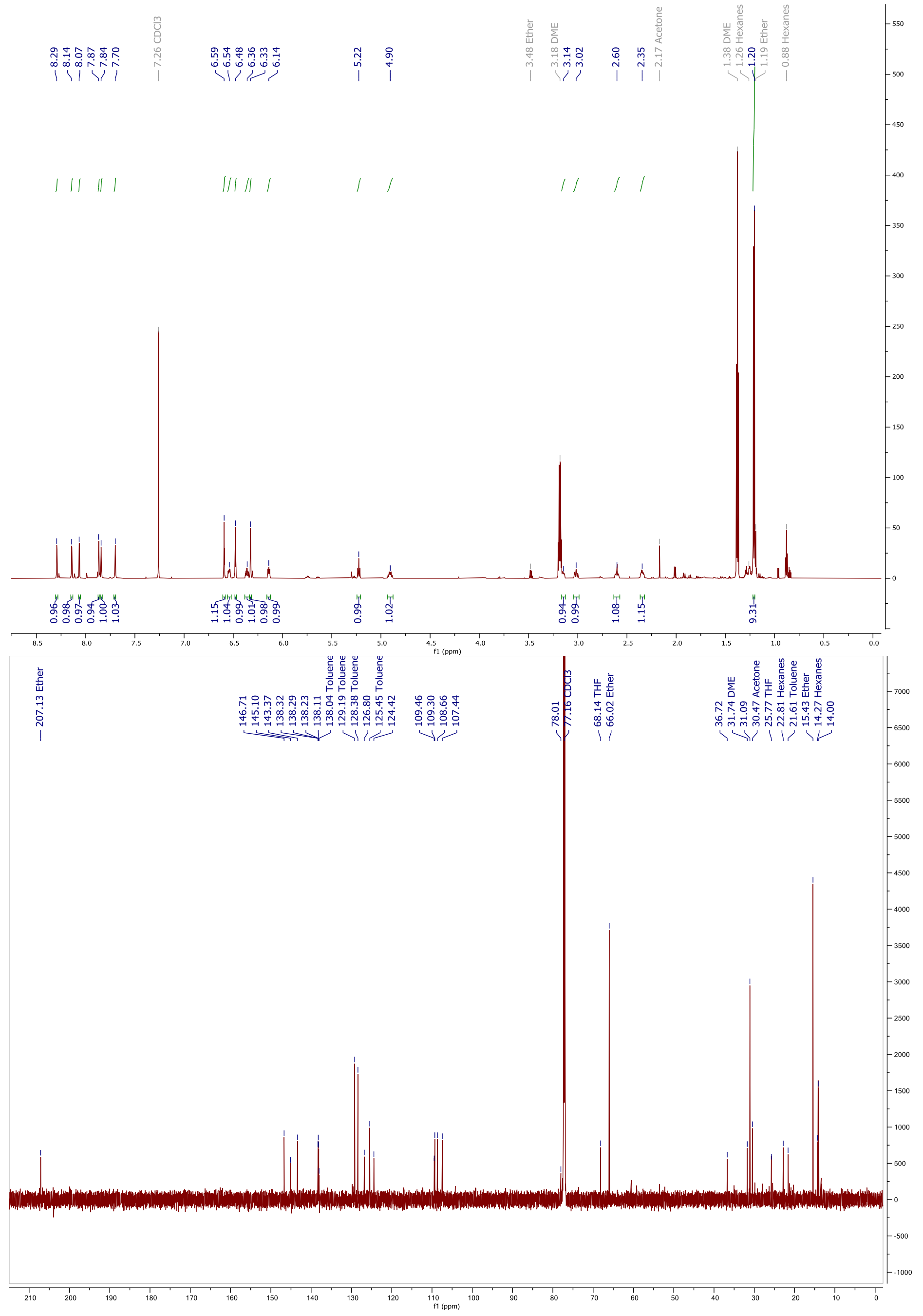

Figure S9: ^{1}H-NMR (CD_3CN) and ^{13}C-NMR (CD_3CN) of Compound 8

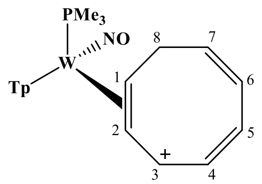

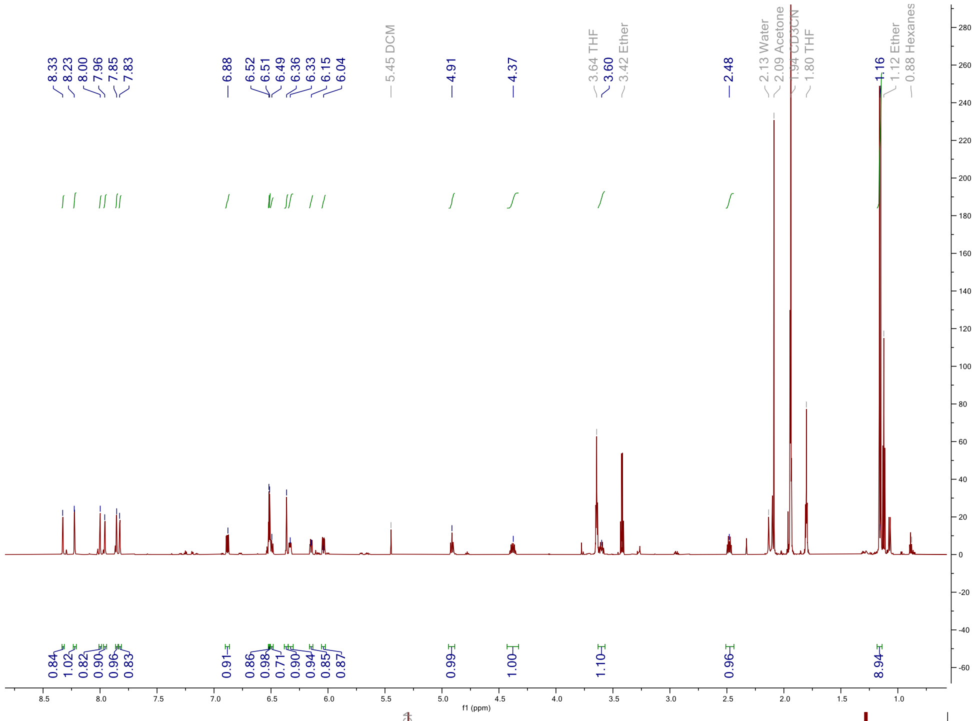

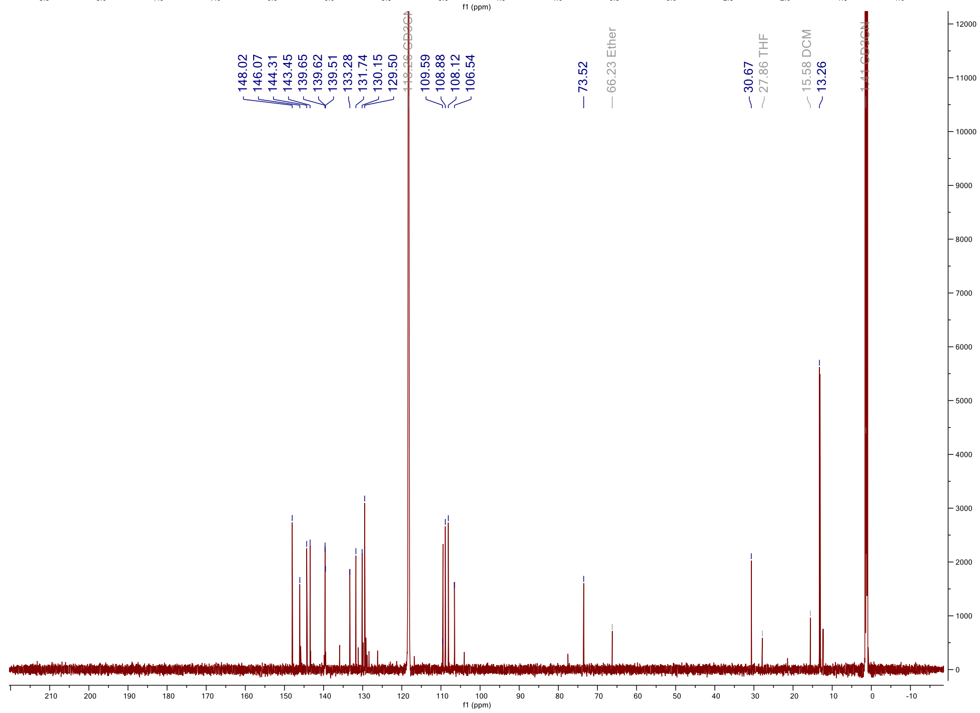

Figure S10: ^{1}H-NMR (CD_3CN) and ^{13}C-NMR (CD_3CN) of Compound 9

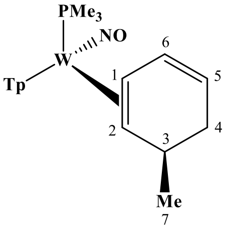

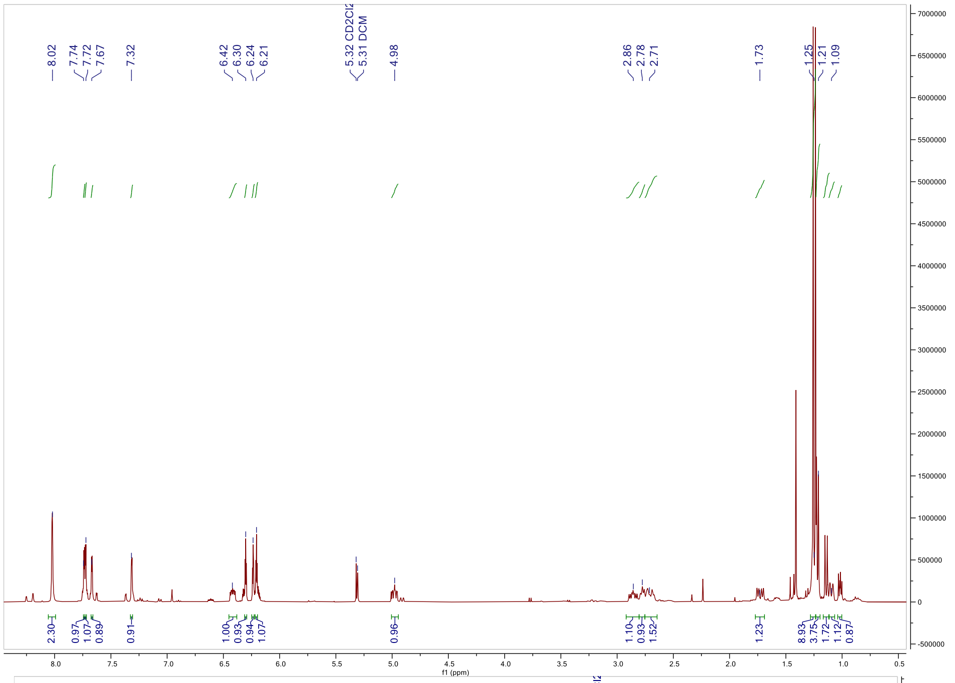

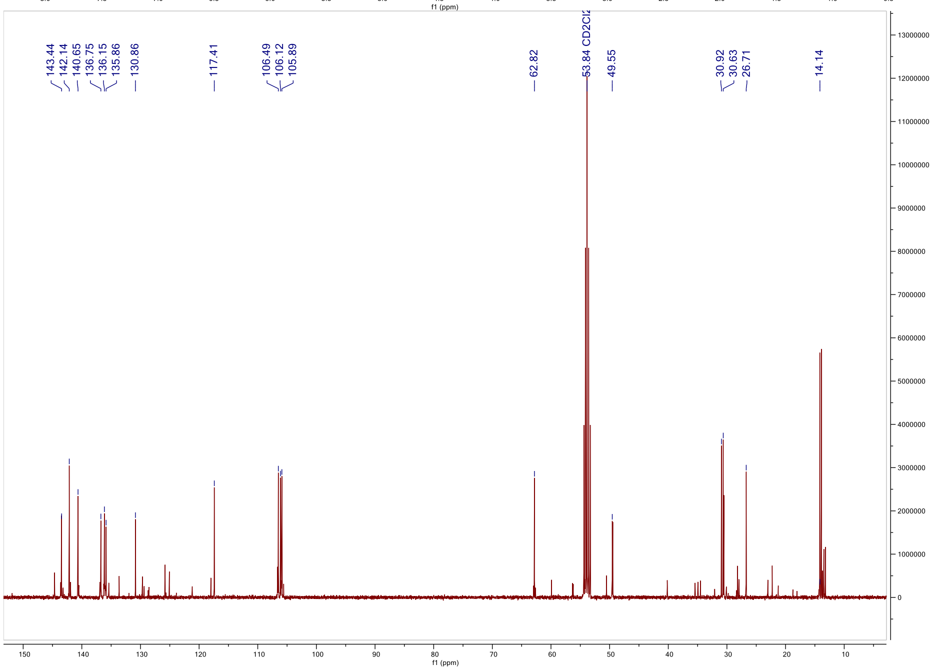

Figure S11: ^{1}H-NMR (CD_3CN) and ^{13}C-NMR (CD_3CN) of Compound 10

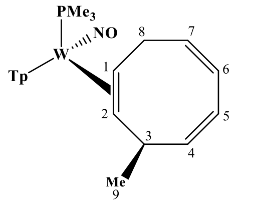

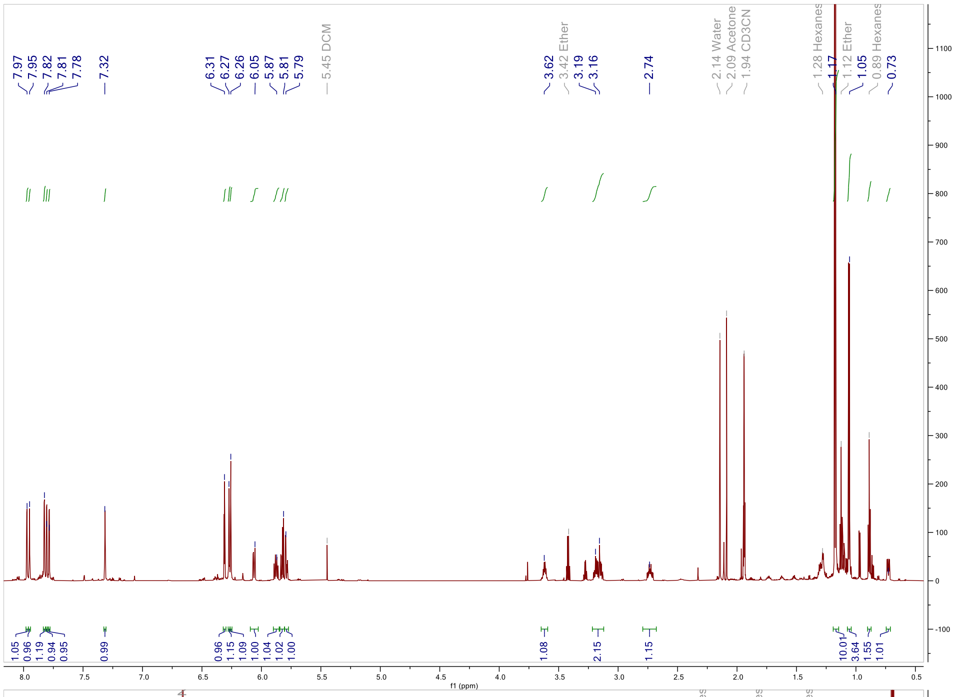

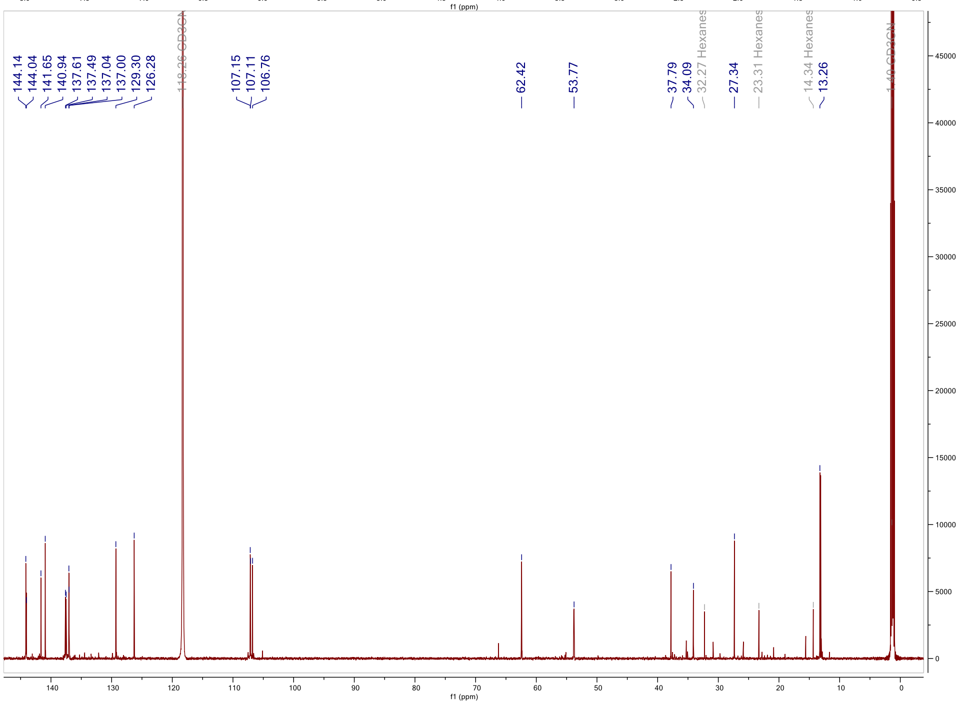

Figure S12: ^{1}H-NMR (CD_3CN) and ^{13}C-NMR (CD_3CN) of Compound 11

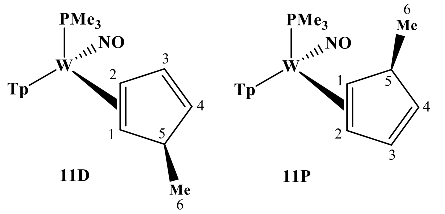

11D 11P

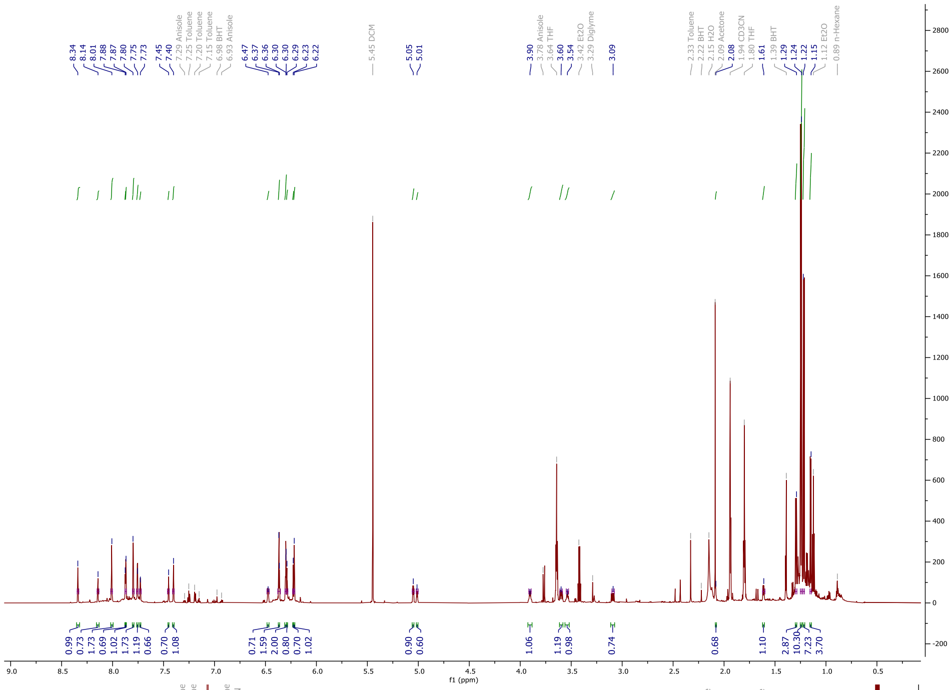

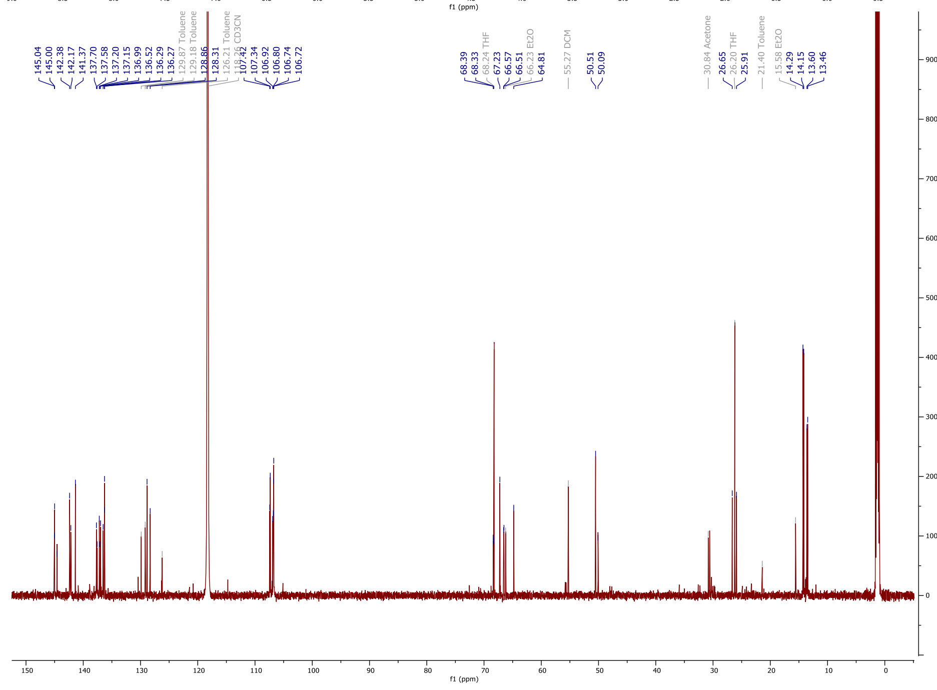

Figure S13: ^{1}H-NMR (CD_3CN) and ^{13}C-NMR (CD_3CN) of Compounds 12D/P/M

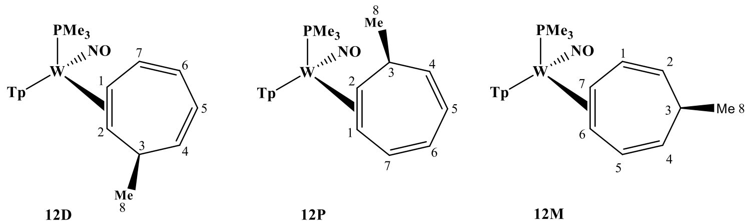

12D 12P 12M

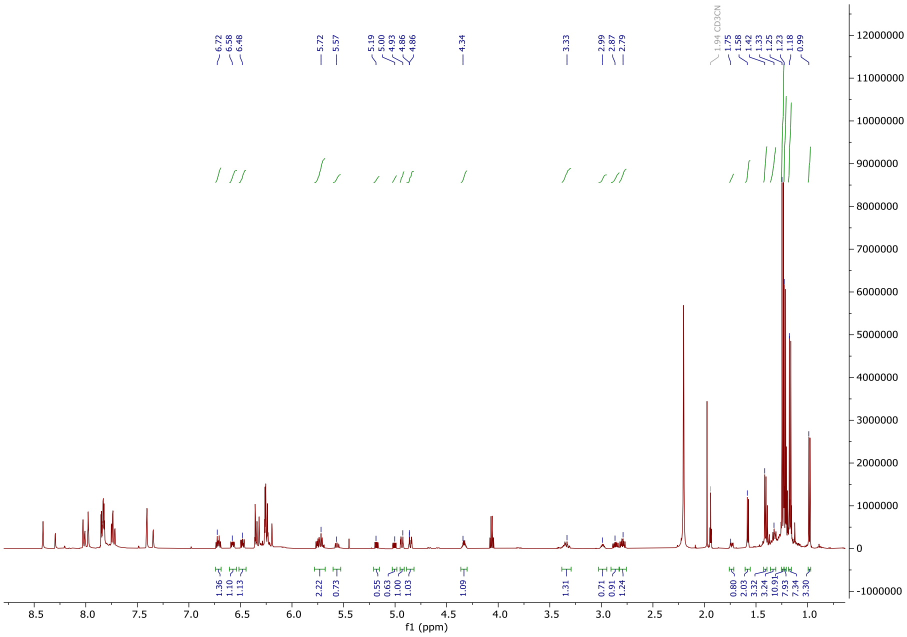

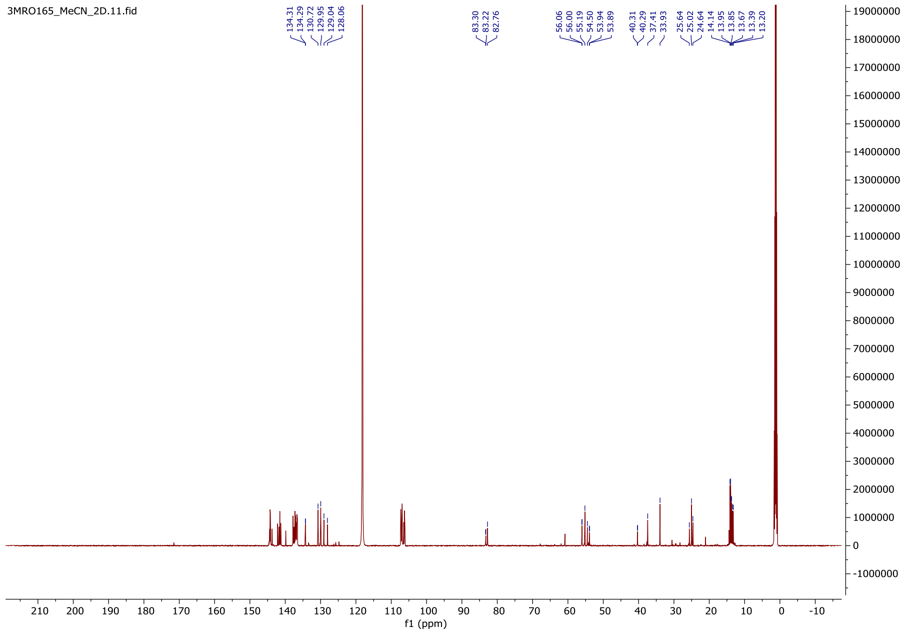

Figure S14: ^{1}H-NMR (CD_3CN) and ^{13}C-NMR (CD_3CN) of Compound 13

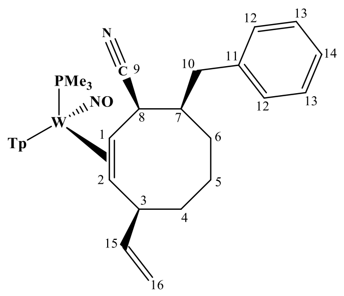

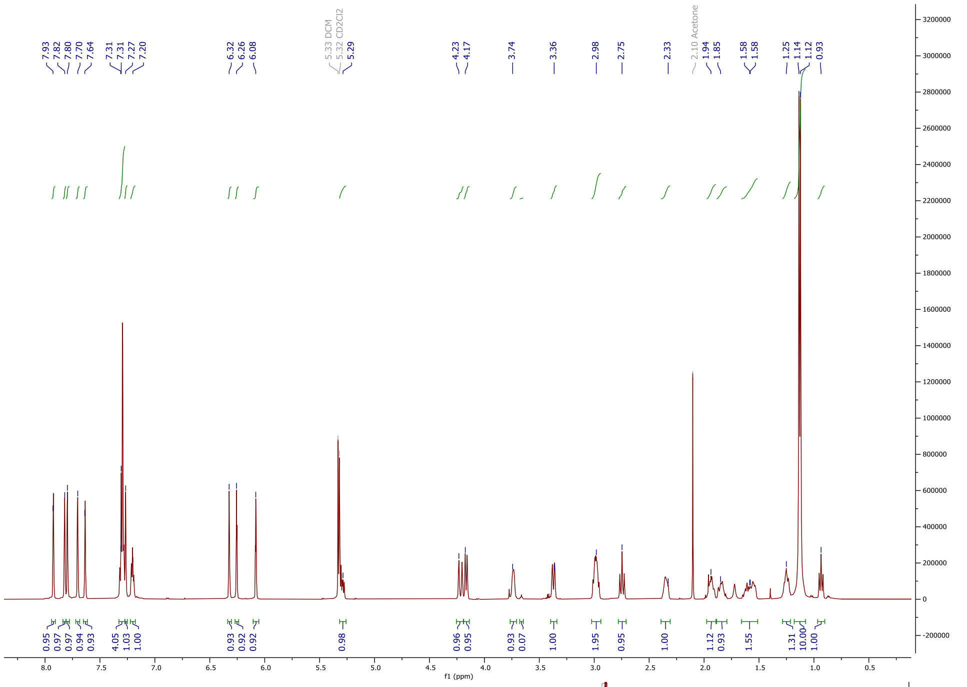

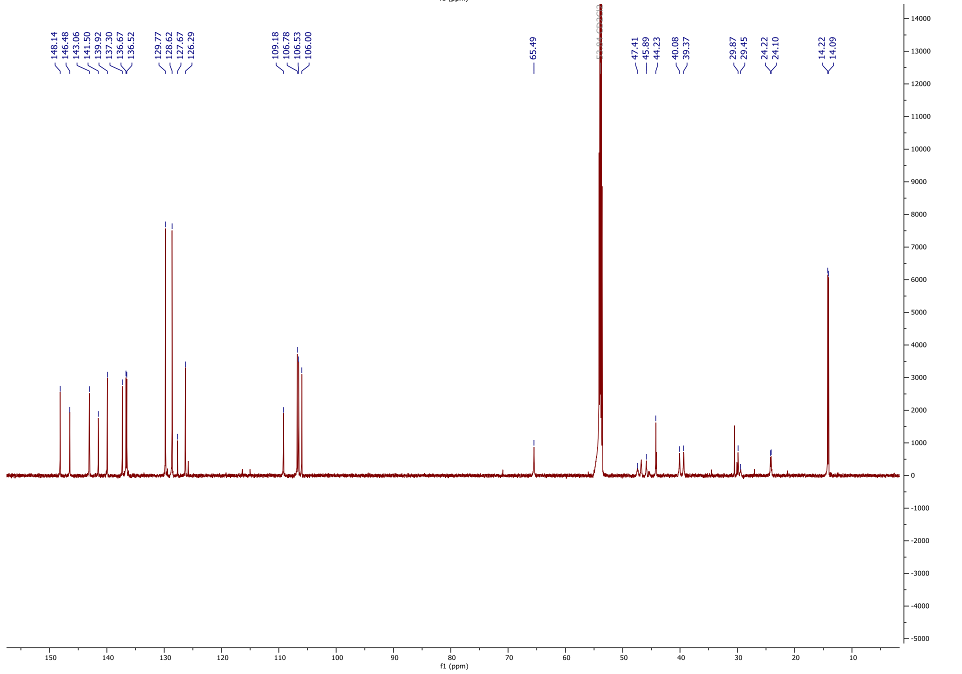

Figure S15: ^{1}H-NMR (CD_3CN) and ^{13}C-NMR (CD_3CN) of Compound 14

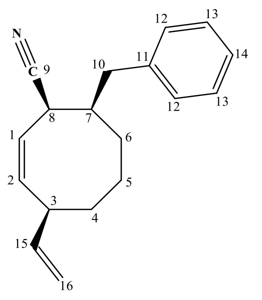

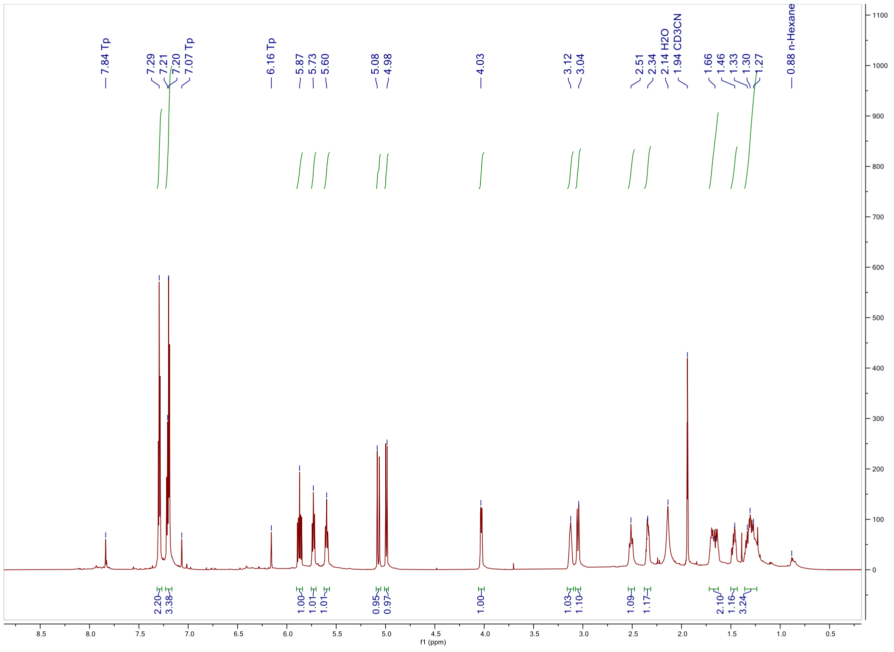

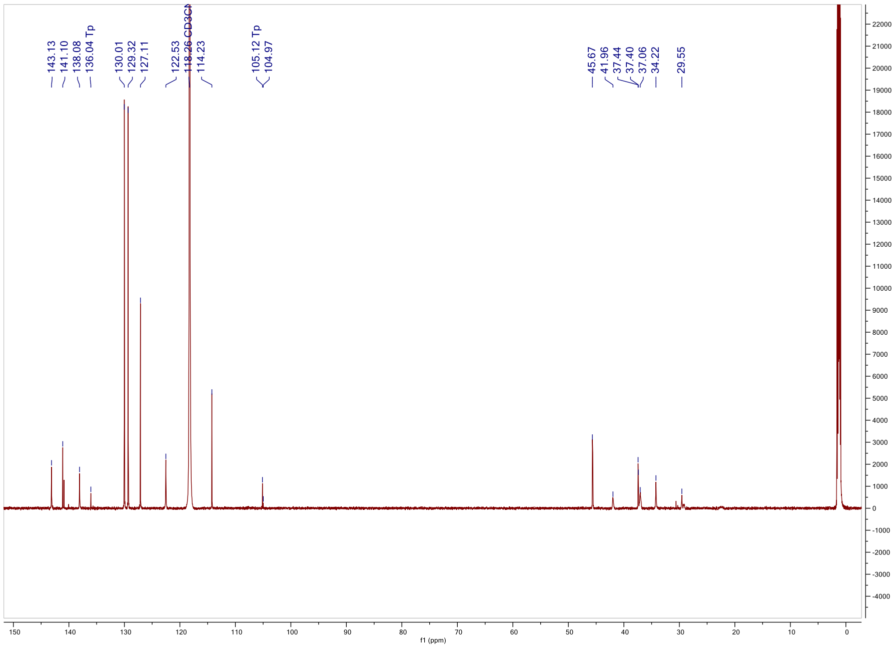

Figure S16: ^{1}H-NMR ($(CD_3)_2CO$) and ^{13}C-NMR ($(CD_3)_2CO$) of Compound 16

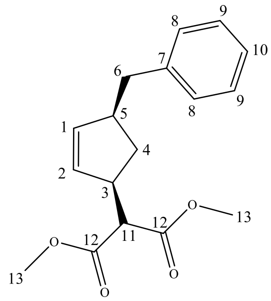

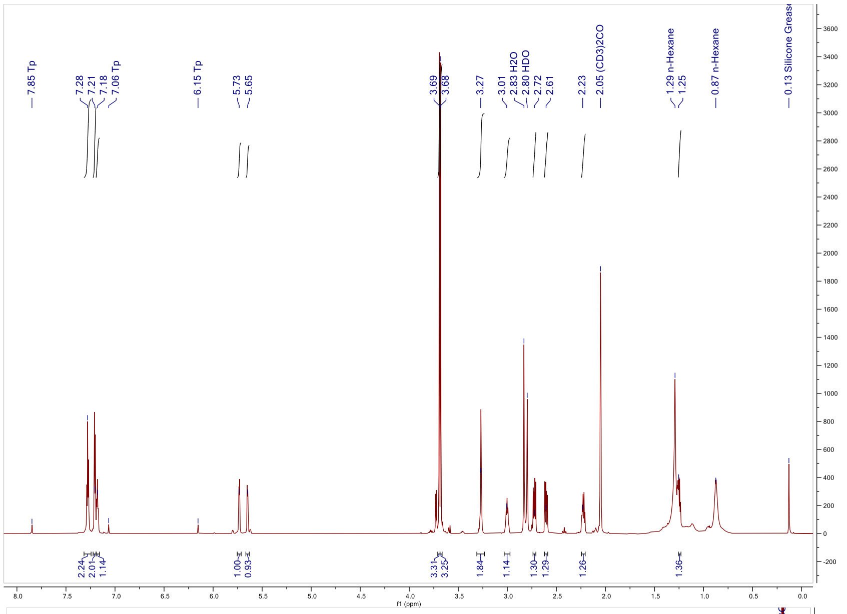

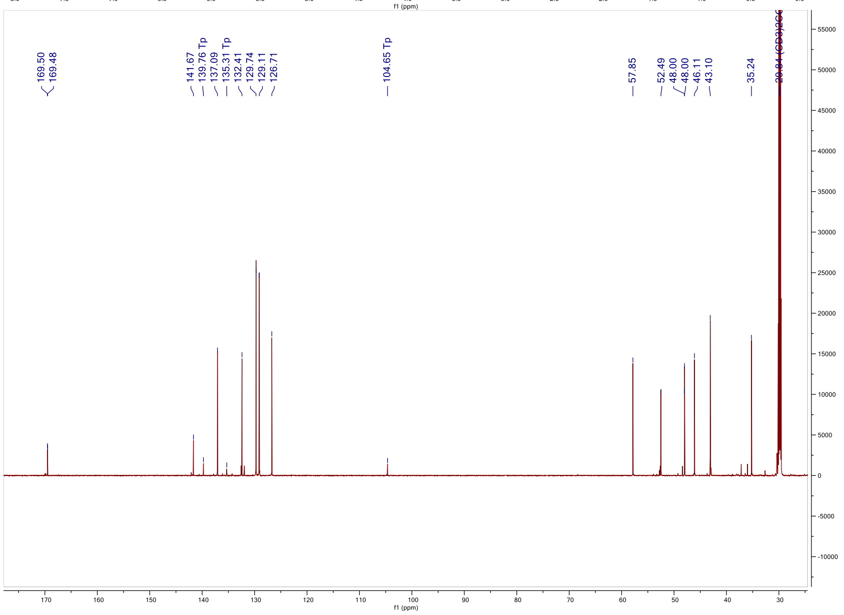

DFT Analysis.

Previous literature demonstrates that an M06 functional and 6-31G** basis set (with the LANL2DZ basis set and core potential on W) accurately corroborates experimental results (see reference 45 of main text). A small benchmarking study was performed against the SC-XRD bond lengths of the η^2-tropylium complex. Based on the results shown below, ground-state structures were optimized at the M062X level of theory using the 6-31G** [LANL2DZ for W] basis set in Gaussian 16. Solvent effects of acetonitrile were modeled using SMD. Gaussian's default criteria were used for optimization, vibrational frequency analysis verified that structures were minima and thermal free energy corrections were applied. IRC calculations were used to confirm transition state studies.

Table S1: benchmarking of functionals and basis sets for η^2-tropylium against SC-XRD data.

Functional	Basis Set	Calculated 'Distal' Conformer (W-C3/W-C8)	XRD Bond Lengths (W-C3/W-C8)
M06	6-31G(d,p) & LANL2DZ on W	2.98/3.20	2.77/3.25
M06	CEP-31G	3.17/3.12	2.77/3.25
M062X	CEP-31G	2.98/3.29	2.77/3.25
**M062X**	**6-31G(d,p) & LANL2DZ on W**	**2.80/3.22**	**2.77/3.25**
B3LYP	CEP-31G	3.25/3.18	2.77/3.25
B3LYP	6-31G(d,p) & LANL2DZ on W	3.09/3.22	2.77/3.25

Figure S17: Comparison of DFT using M062X/6-31G(d,p) & LANL2DZ on W and SC-XRD data of Compound 2.

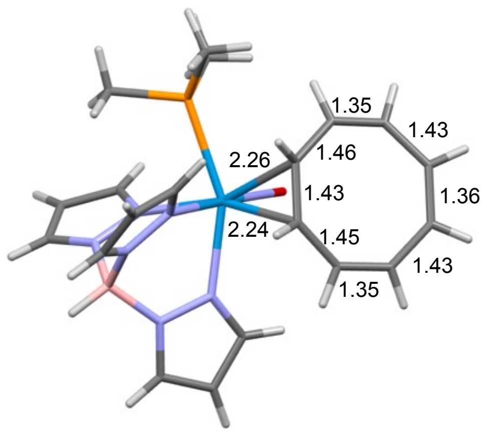

Bond Lengths via SC-XRD.

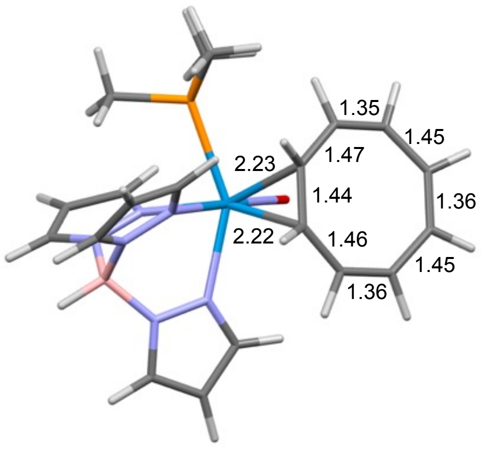

Bond Lengths via DFT.

Figure S18: Comparison of DFT using M062X/6-31G(d,p) & LANL2DZ on W and SC-XRD data of Compound 3.

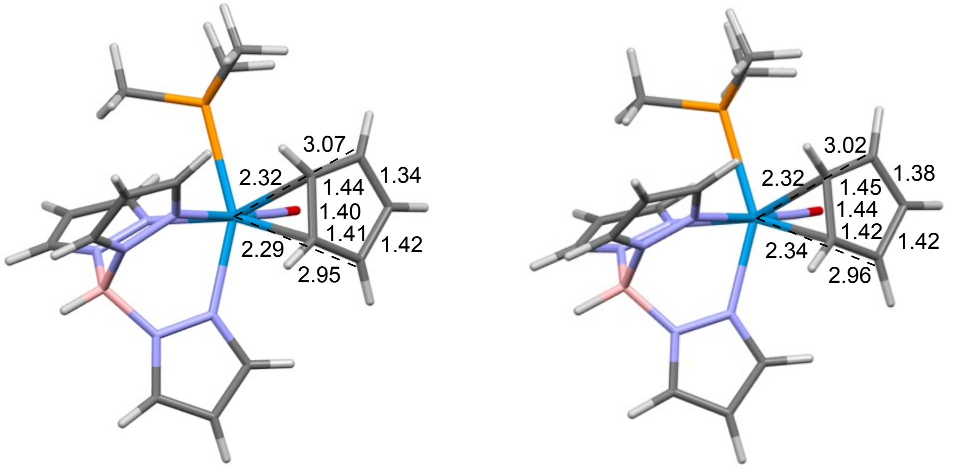

Bond Lengths via SC-XRD. **Bond Lengths via DFT.**

Figure S19: Comparison of DFT using M062X/6-31G(d,p) & LANL2DZ on W and SC-XRD data of Compound 4.

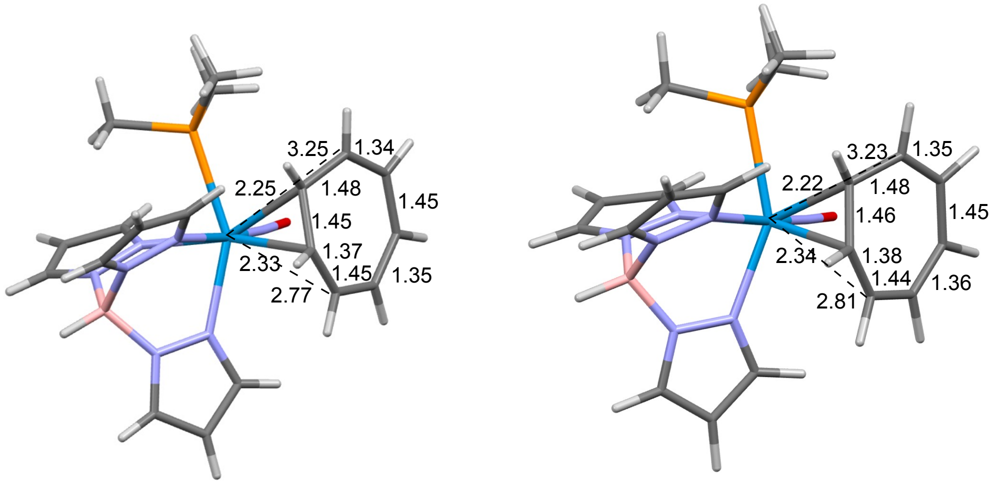

Bond Lengths via SC-XRD. **Bond Lengths via DFT.**

Figure S20: Reaction Mechanism of tungsten-alkene isomerization for η^2-COT.

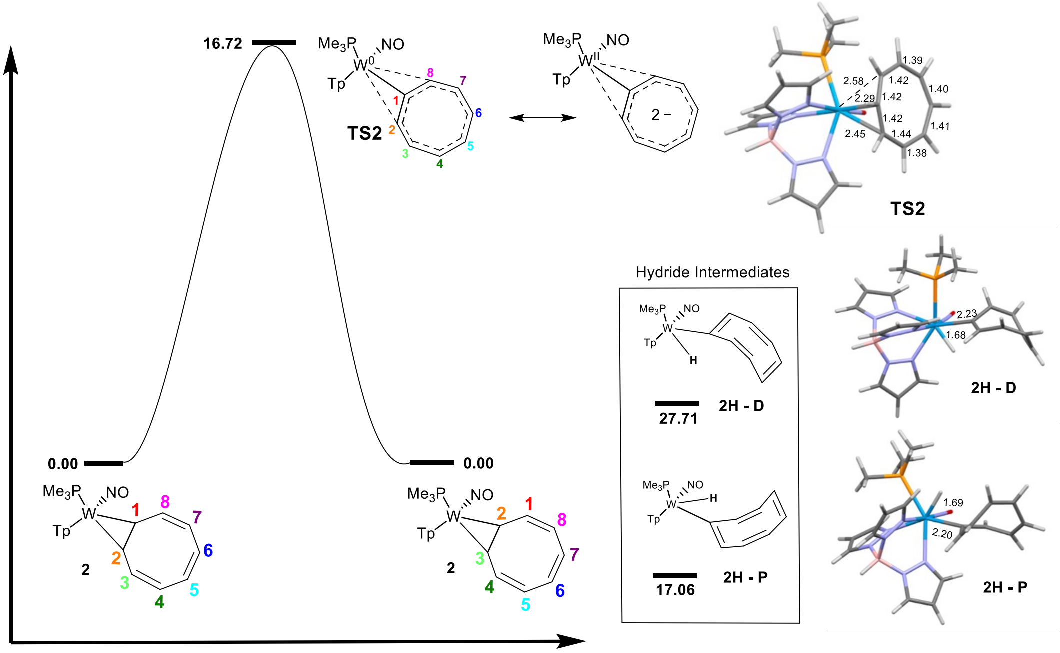

*Mechanistic studies were performed with the conformation resembling the SC-XRD structure, although a lower energy minimum was identified in solution through DFT analysis, named compound 2B.

Figure S21: Reaction Mechanism of tungsten-alkene isomerization for η^2-Cp.

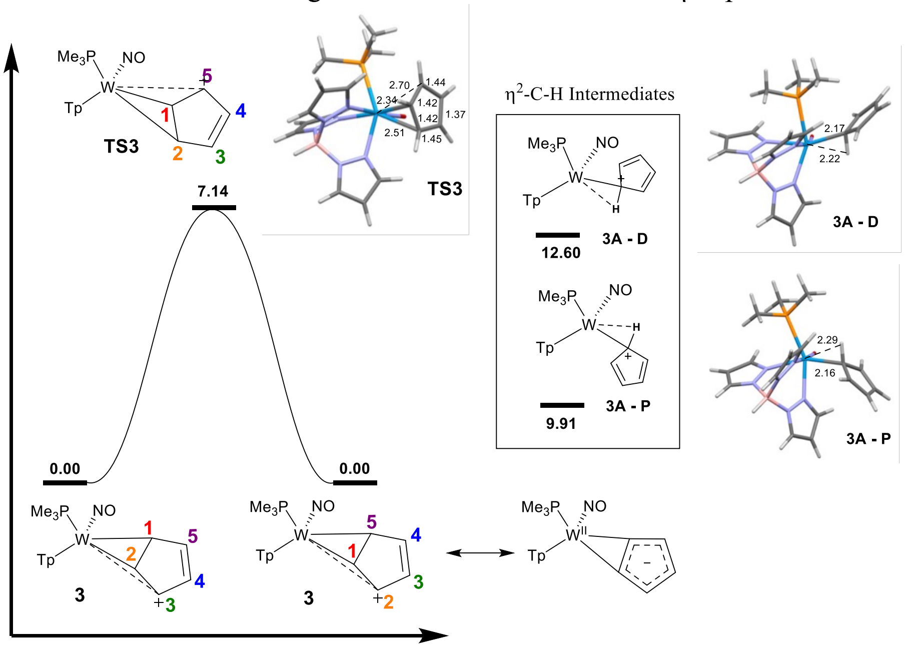

Figure S22: Reaction Mechanism of tungsten-alkene isomerization for η^2-Tropylium.

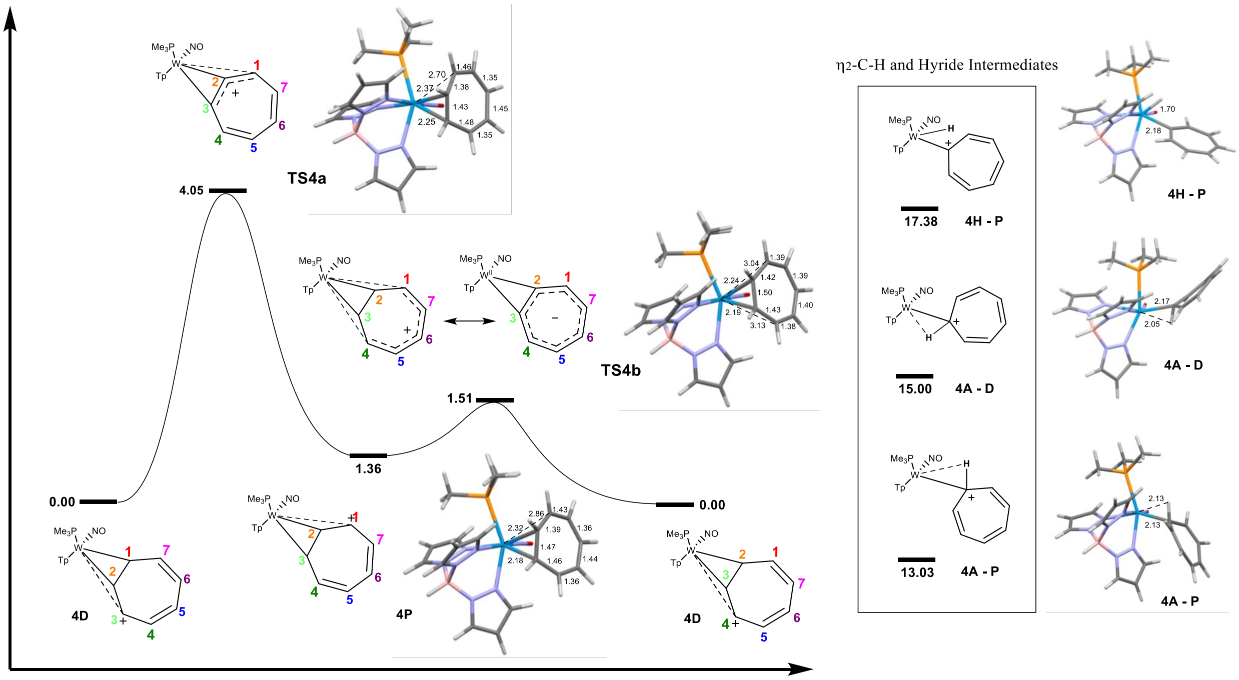

Table S2: Energy values of optimized minima and transition state structures.

Structure	Electronic Energy (Hartree)	Relative Free Energy (kcal/mol)
**2**	-1669.909974	0.00
**TS2**	-1669.883334	+16.72
**2H – D**	-1669.867919	+27.71
**2H – P**	-1669.882783	+17.06
**2B**	-1669.912079	- 1.32
**3**	-1553.774705	0.00
**TS3**	-1553.763299	+ 7.16
**3A – D**	-1553.754627	+12.60
**3A – P**	-1553.758907	+ 9.91
**4D**	-1631.116013	0.00
**4P**	-1631.113800	+ 1.36
**TS4a**	-1631.109536	+ 4.05
**TS4b**	-1631.113580	+ 1.51
**4H – P**	-1631.088290	+17.38
**4A – D**	-1631.092080	+15.00
**4A – P**	-1631.095218	+13.03

Table S3: Compound 2 Electronic Energy: -1669.909974 Hartree

W	-0.269900	0.000800	-0.080800
O	-1.904000	0.345800	-2.519700
N	-1.267800	0.196200	-1.506100
P	0.038500	-2.533100	-0.175300
C	-0.497600	-3.574800	1.235600
H	-1.562100	-3.473500	1.449000
H	-0.280900	-4.620300	0.998100
H	0.071400	-3.292100	2.124900
C	-0.698000	-3.361700	-1.627900
H	-0.286500	-2.922100	-2.540000
H	-0.480400	-4.433300	-1.611300
H	-1.780000	-3.205200	-1.621500
C	1.808900	-3.012500	-0.243900
H	2.340900	-2.536000	0.585900
H	1.893300	-4.098600	-0.146700
H	2.264500	-2.700600	-1.185500
N	1.209400	-0.227000	1.679300
N	2.474400	0.228400	1.574600
N	2.802500	0.189700	-0.934100
N	1.578600	-0.170300	-1.363100
N	1.969500	2.245600	0.155500
N	0.651900	2.071300	-0.097000
C	1.106500	-0.806300	2.880100
H	0.170300	-1.240900	3.202800
C	2.325200	-0.731700	3.562600
H	2.556600	-1.105400	4.547800
C	3.166500	-0.059800	2.690400
H	4.200600	0.239000	2.784300
C	1.710400	-0.621200	-2.612700
H	0.841500	-0.963600	-3.159000
C	3.054400	-0.566200	-3.001300
H	3.483600	-0.860900	-3.946200
C	3.710100	-0.032900	-1.902100
H	4.749400	0.218100	-1.745000
C	0.168800	3.255300	-0.481000
H	-0.874700	3.353800	-0.744500
C	1.181000	4.221700	-0.464500
H	1.100200	5.268500	-0.712500
C	2.309300	3.530200	-0.058800
H	3.329800	3.854000	0.087400
B	2.930300	1.044100	0.351700
H	4.054900	1.421400	0.497200
C	-1.986300	-0.593300	1.217200
H	-1.625400	-1.216500	2.036900
C	-1.708500	0.805400	1.408200
H	-1.097300	0.980000	2.296800
C	-2.397100	2.036000	1.028900
H	-1.787400	2.905300	1.273600
C	-3.593400	2.359900	0.481300

H	-3.744700	3.432100	0.365400
C	-4.750500	1.598000	0.054700
H	-5.600000	2.244800	-0.161600
C	-5.006600	0.284100	-0.161100
H	-6.018600	0.094500	-0.516900
C	-4.235500	-0.942400	-0.073200
H	-4.757700	-1.779300	-0.535000
C	-3.048600	-1.287900	0.477100
H	-2.827900	-2.348500	0.360800

Table S4: Compound TS2 Electronic Energy: -1669.883334 Hartree

W	-0.315900	0.161400	0.070800
O	-1.936800	0.601300	-2.340600
N	-1.356100	0.471100	-1.303600
P	-0.412900	-2.387800	-0.335900
C	-1.023000	-3.610400	0.886600
H	-2.101900	-3.524800	1.023200
H	-0.795000	-4.609400	0.504400
H	-0.519500	-3.475200	1.847000
C	-1.385100	-2.807600	-1.822300
H	-0.953500	-2.330700	-2.704400
H	-1.397700	-3.891300	-1.967400
H	-2.406200	-2.441400	-1.684200
C	1.257400	-3.081400	-0.654000
H	1.904000	-2.869100	0.204300
H	1.173600	-4.164800	-0.775800
H	1.703100	-2.657600	-1.555300
N	1.295600	-0.516800	1.579700
N	2.608000	-0.344500	1.336800
N	2.608700	-0.125600	-1.178600
N	1.291100	-0.166500	-1.455000
N	2.354900	1.887600	0.208900
N	1.010300	2.003300	0.100700
C	1.197900	-1.302000	2.658100
H	0.225200	-1.582400	3.041200
C	2.472000	-1.644000	3.122700
H	2.724900	-2.255400	3.974800
C	3.340000	-1.007700	2.248100
H	4.419500	-0.973100	2.212600
C	1.163600	-0.423600	-2.760600
H	0.180500	-0.477200	-3.209000
C	2.426700	-0.573800	-3.341200
H	2.659500	-0.790100	-4.372100
C	3.315500	-0.362500	-2.297200
H	4.395800	-0.346800	-2.273300
C	0.742300	3.306300	-0.023700
H	-0.279800	3.638000	-0.128400
C	1.923900	4.051700	0.009400
H	2.032400	5.122500	-0.062600
C	2.923500	3.104700	0.156300

H	3.997300	3.205600	0.222500
B	3.073400	0.516600	0.151900
H	4.259900	0.653500	0.166500
C	-1.687800	0.426500	1.887000
H	-1.016500	0.252200	2.723700
C	-1.633900	1.752500	1.378900
H	-0.893800	2.343000	1.916600
C	-2.540700	2.573600	0.629300
H	-2.235800	3.617400	0.598900
C	-3.727700	2.310600	-0.025900
H	-4.168800	3.213800	-0.451100
C	-4.516200	1.169800	-0.290500
H	-5.414900	1.409900	-0.856600
C	-4.374900	-0.202800	-0.062400
H	-5.183200	-0.777400	-0.518800
C	-3.478300	-1.023800	0.618500
H	-3.708000	-2.085900	0.556200
C	-2.329400	-0.750200	1.406600
H	-1.985800	-1.615400	1.968100

Table S5: Compound 2H – D Electronic Energy: -1669.867919 Hartree

W	-0.049300	0.278800	-0.386200
O	-0.607000	0.299500	-3.283600
N	-0.417800	0.327100	-2.093300
H	-0.920600	1.683200	-0.101400
P	-0.413400	-2.312600	-0.140200
C	-1.782100	-2.958400	0.891200
H	-2.748900	-2.641500	0.495000
H	-1.732400	-4.051000	0.894400
H	-1.683000	-2.591500	1.915100
C	-0.627300	-3.244700	-1.704000
H	0.261900	-3.133100	-2.327500
H	-0.773200	-4.305900	-1.482300
H	-1.491900	-2.870000	-2.256900
C	1.042000	-3.139200	0.612800
H	1.262300	-2.694200	1.587400
H	0.825000	-4.203200	0.745100
H	1.914100	-3.030800	-0.036700
N	0.755300	-0.035600	1.795200
N	2.073500	0.101300	2.055500
N	3.102800	-0.418600	-0.178500
N	1.994200	-0.570300	-0.924000
N	2.562600	1.927700	0.395200
N	1.375000	2.021700	-0.240500
C	0.200200	-0.541700	2.902800
H	-0.858700	-0.750400	2.934000
C	1.167600	-0.733400	3.893800
H	1.026700	-1.117200	4.892200
C	2.347800	-0.308300	3.305900
H	3.358800	-0.263900	3.684800

C	2.345100	-1.237500	-2.026600
H	1.607700	-1.444900	-2.790400
C	3.709700	-1.546200	-1.984900
H	4.290800	-2.080900	-2.720000
C	4.152300	-0.992300	-0.792800
H	5.136200	-0.952000	-0.347700
C	1.257200	3.280600	-0.669600
H	0.366900	3.587600	-1.200300
C	2.390100	4.019300	-0.314500
H	2.593600	5.058900	-0.518600
C	3.191600	3.115800	0.364300
H	4.161600	3.229700	0.826700
B	3.071400	0.592300	0.989500
H	4.159000	0.728300	1.466200
C	-2.191300	0.099600	0.210200
C	-2.695900	0.384800	1.423800
H	-2.027100	0.771700	2.195400
C	-4.087700	0.186900	1.890900
H	-4.210600	-0.445200	2.772800
C	-5.178100	0.801400	1.413900
H	-6.128500	0.643200	1.923900
C	-5.204700	1.757600	0.293800
H	-5.733000	2.692700	0.479900
C	-4.724600	1.540900	-0.939900
H	-4.889800	2.311900	-1.692600
C	-4.116100	0.289000	-1.421100
H	-4.549300	-0.110500	-2.338900
C	-3.069900	-0.363200	-0.889700
H	-2.743300	-1.256800	-1.424000

Table S6: Compound 2H - P Electronic Energy: -1669.882783 Hartree

W	0.031300	-0.364100	-0.469300
O	-0.208800	-1.359300	-3.245600
N	-0.109700	-0.945700	-2.116700
H	-0.961200	-1.563100	0.192600
P	1.260200	-2.441400	0.307000
C	0.355800	-3.596800	1.394500
H	-0.532700	-3.959300	0.871800
H	0.998400	-4.441900	1.657300
H	0.045300	-3.083500	2.307200
C	1.814600	-3.543400	-1.040600
H	2.507500	-3.021400	-1.703100
H	2.310200	-4.424600	-0.623500
H	0.940700	-3.856500	-1.618400
C	2.774800	-2.093900	1.277100
H	2.514700	-1.460100	2.130900
H	3.202600	-3.032100	1.642000
H	3.513300	-1.576400	0.660100
N	0.348900	0.286700	1.703300
N	1.188200	1.290600	2.025800

N	2.762000	1.235700	0.052300
N	2.196800	0.285200	-0.716200
N	0.771100	2.707700	-0.008900
N	-0.033000	1.900900	-0.735600
C	-0.108700	-0.223000	2.850500
H	-0.821700	-1.036000	2.834300
C	0.447800	0.457900	3.938400
H	0.273900	0.284300	4.988800
C	1.266800	1.417000	3.361700
H	1.892600	2.179500	3.803000
C	3.131600	-0.142500	-1.568500
H	2.892300	-0.903300	-2.298700
C	4.335700	0.532600	-1.338800
H	5.273400	0.410600	-1.858100
C	4.047400	1.408200	-0.303200
H	4.656900	2.149100	0.194000
C	-0.792000	2.687800	-1.500600
H	-1.529700	2.248900	-2.157700
C	-0.478000	4.031700	-1.271700
H	-0.914800	4.902200	-1.735600
C	0.523100	3.993800	-0.314400
H	1.078400	4.785000	0.168600
B	1.864600	2.139500	0.931500
H	2.503400	3.023000	1.421500
C	-2.116400	0.113800	-0.370800
C	-2.960200	-0.115000	-1.394700
H	-2.568600	-0.592200	-2.296700
C	-4.388400	0.257300	-1.486400
H	-4.683100	0.797100	-2.388200
C	-5.366900	-0.111900	-0.647000
H	-6.392800	0.149500	-0.907800
C	-5.215200	-0.931400	0.568600
H	-5.900900	-1.774500	0.655900
C	-4.393800	-0.681000	1.598900
H	-4.456600	-1.332800	2.470700
C	-3.497800	0.483100	1.717900
H	-3.598900	1.060700	2.637700
C	-2.552900	0.848500	0.839700
H	-1.949200	1.723600	1.092500

Table S7: Compound 2B Electronic Energy-1669.912079 Hartree

W	-0.282100	0.040000	-0.020200
O	-1.796900	0.190100	-2.545400
N	-1.274900	0.198100	-1.452800
P	-0.458900	-2.478300	-0.101500
C	-1.057300	-3.429200	1.343900
H	-2.082000	-3.149200	1.591500
H	-1.025600	-4.496200	1.105300
H	-0.413700	-3.241300	2.206900
C	-1.539300	-3.067600	-1.451100

H	-1.219400	-2.621500	-2.396200
H	-1.513100	-4.158100	-1.527600
H	-2.561600	-2.736900	-1.243800
C	1.147100	-3.316500	-0.397300
H	1.866300	-2.977500	0.355800
H	1.020800	-4.399500	-0.312600
H	1.536300	-3.076500	-1.388300
N	1.323400	-0.373500	1.604300
N	2.614400	-0.043200	1.392600
N	2.725900	-0.118400	-1.125300
N	1.437300	-0.373300	-1.414100
N	2.219900	2.022600	0.017200
N	0.873400	1.998500	-0.092400
C	1.271200	-0.971500	2.799400
H	0.329600	-1.323000	3.197500
C	2.546500	-1.034000	3.370300
H	2.825700	-1.453800	4.324000
C	3.370000	-0.427400	2.435800
H	4.433600	-0.236500	2.439100
C	1.392300	-0.840700	-2.663500
H	0.438600	-1.095500	-3.108400
C	2.686500	-0.907000	-3.194600
H	2.984700	-1.246200	-4.174400
C	3.501300	-0.426000	-2.180900
H	4.569400	-0.267500	-2.139300
C	0.473500	3.258200	-0.274700
H	-0.579700	3.477700	-0.382100
C	1.576300	4.120900	-0.280600
H	1.578300	5.192600	-0.404500
C	2.665900	3.287500	-0.091500
H	3.723700	3.499300	-0.028500
B	3.055100	0.722400	0.129200
H	4.223000	0.973500	0.179500
C	-4.198000	-0.379400	0.298600
H	-5.089600	-1.007100	0.288800
C	-3.246100	-0.756900	1.173300
H	-3.526300	-1.622600	1.774200
C	-1.884800	-0.270400	1.494500
H	-1.482700	-0.850800	2.324100
C	-1.439500	1.110900	1.508300
H	-0.710100	1.279800	2.303200
C	-2.243700	2.336100	1.315000
H	-1.932800	3.136900	1.987500
C	-3.230900	2.688700	0.469200
H	-3.575000	3.719400	0.556400
C	-3.839800	1.956800	-0.642700
H	-4.042700	2.574300	-1.518300
C	-4.226700	0.674800	-0.719500
H	-4.702500	0.370400	-1.651400

Table S8: Compound 3 Electronic Energy: -1553.774705 Hartree

O	-1.782400	-1.327200	2.517700
N	-1.224000	-0.987700	1.529900
W	-0.417700	-0.413800	0.081600
N	1.469700	-1.553300	0.184800
N	2.661700	-0.967800	-0.061000
N	0.678500	0.545200	-1.688500
N	1.967700	0.922800	-1.546200
N	0.893600	0.816100	1.354700
N	2.118700	1.217400	0.955900
C	3.640700	-1.880000	0.042100
C	3.072200	-3.104700	0.361000
C	1.703700	-2.845200	0.444800
C	2.393600	1.553600	-2.652200
C	1.346600	1.597000	-3.558700
C	0.295100	0.948700	-2.907100
C	2.706300	1.915300	1.940200
C	1.833800	1.981100	3.018100
C	0.713400	1.259900	2.605700
B	2.771600	0.562500	-0.286800
H	3.910200	0.900100	-0.397000
H	4.670400	-1.591400	-0.113000
H	3.576500	-4.045700	0.515000
H	0.883800	-3.510600	0.675500
H	3.409000	1.917700	-2.715300
H	1.345800	2.025900	-4.548400
H	-0.703500	0.756400	-3.270500
H	3.708400	2.298800	1.811700
H	1.993300	2.468800	3.966900
H	-0.203000	1.038800	3.135800
C	-0.498900	3.244700	0.098900
H	-1.077900	4.167300	0.003400
H	0.092100	3.284500	1.016100
H	0.169800	3.147200	-0.762100
C	-2.704900	2.058900	1.593700
H	-2.113800	1.986100	2.507900
H	-3.182900	3.041300	1.549100
H	-3.471600	1.279100	1.600900
P	-1.650500	1.827800	0.126100
C	-2.776200	2.214600	-1.256300
H	-3.176500	3.219600	-1.095500
H	-2.231100	2.193600	-2.202500
H	-3.601700	1.500800	-1.289900
C	-3.209300	-1.314200	-0.632000
C	-2.089200	-0.893300	-1.449200
C	-1.166500	-1.991500	-1.471500
C	-1.700500	-2.988800	-0.615200
C	-2.979100	-2.591600	-0.152600
H	-2.173500	-0.135500	-2.215900
H	-0.335900	-2.122300	-2.155600

H	-3.594600	-3.137400	0.549600
H	-4.067500	-0.689900	-0.404800
H	-1.200600	-3.917900	-0.362600

Table S9: Compound TS3 Electronic Energy: -1553.763299 Hartree

W	-0.427300	0.430500	0.000800
O	-1.681600	1.524500	-2.409000
N	-1.171200	1.113400	-1.426200
P	-1.706300	-1.787800	-0.315800
C	-0.551300	-3.202600	-0.394500
H	-1.137000	-4.122500	-0.472400
H	0.106000	-3.122600	-1.262000
H	0.050600	-3.235300	0.518700
C	-2.667500	-1.844800	-1.862000
H	-2.012600	-1.738600	-2.728500
H	-3.193800	-2.801400	-1.924200
H	-3.393400	-1.026900	-1.855300
C	-2.922300	-2.337800	0.933100
H	-3.110800	-3.403200	0.772600
H	-2.551300	-2.186000	1.948000
H	-3.858700	-1.793600	0.797700
B	2.766500	-0.611600	0.300200
H	3.903100	-0.963400	0.382200
N	1.491900	1.549300	-0.040900
N	2.667600	0.933700	0.214900
N	0.649200	-0.781800	1.644300
N	1.946400	-1.119500	1.494500
N	0.893300	-0.682600	-1.364700
N	2.117600	-1.126600	-1.010600
C	1.756100	2.841200	-0.265400
H	0.952100	3.529500	-0.487000
C	3.127500	3.072100	-0.148800
H	3.651500	4.007300	-0.267600
C	3.665900	1.829600	0.151200
H	4.687000	1.517100	0.316100
C	0.209700	-1.419900	2.737000
H	-0.813600	-1.308700	3.067000
C	1.236000	-2.181100	3.300500
H	1.193500	-2.797900	4.184400
C	2.325500	-1.957100	2.473100
H	3.339900	-2.327300	2.512100
C	0.723300	-0.970700	-2.662800
H	-0.187500	-0.687100	-3.171800
C	1.847400	-1.634700	-3.152100
H	2.013700	-2.002700	-4.152200
C	2.713000	-1.697900	-2.068500
H	3.715600	-2.091400	-1.980400
C	-1.581200	1.316700	1.837700
H	-1.157200	0.802500	2.688500
C	-1.083300	2.503200	1.247000

H	-0.211800	3.044200	1.591700
C	-2.136300	3.042900	0.405400
H	-2.067700	3.971500	-0.148600
C	-3.165000	2.137700	0.360800
H	-4.061600	2.203200	-0.242000
C	-2.789900	1.001800	1.170400
H	-3.427100	0.161700	1.408600

Table S10: Compound 3A – D Electronic Energy: -1553.754627 Hartree

W	0.034200	0.121400	0.596400
O	-0.338400	2.577000	2.175200
N	-0.204400	1.538400	1.602400
C	-5.176000	-0.436300	0.494200
H	-6.192300	-0.069700	0.375400
C	-4.861800	-1.602300	-0.110900
H	-5.646200	-2.101300	-0.674000
C	-3.599500	-2.314300	-0.039400
H	-3.631200	-3.331700	-0.423200
C	-2.402900	-1.909400	0.438700
H	-1.614700	-2.660300	0.426100
C	-4.319100	0.378000	1.337600
H	-4.832800	1.179800	1.863000
C	-2.988700	0.299700	1.558100
H	-2.582000	1.031100	2.254600
C	-1.967800	-0.605900	0.985400
P	-1.216100	1.279000	-1.285900
C	-0.202600	1.544900	-2.783300
H	-0.852400	1.905500	-3.585400
H	0.586100	2.276400	-2.602800
C	-2.711300	0.494900	-1.976100
H	-2.484500	-0.486800	-2.393800
H	-3.474700	0.399000	-1.204600
C	-1.834600	2.920300	-0.791300
H	-2.345400	3.388300	-1.637200
H	-2.541300	2.790100	0.033900
H	-3.086300	1.142600	-2.774000
H	0.249200	0.594200	-3.083400
H	-1.016400	3.561600	-0.459500
N	0.511600	-1.420000	-1.070200
N	1.793300	-1.598500	-1.461300
N	2.752400	0.649400	-0.932400
N	1.670500	1.241800	-0.387200
N	2.897400	-1.164900	0.755700
N	1.848600	-0.765800	1.506600
C	-0.244600	-2.130000	-1.918700
H	-1.317500	-2.160200	-1.801900
C	0.553100	-2.770200	-2.868900
H	0.235100	-3.413700	-3.674000
C	1.846400	-2.400400	-2.536400
H	2.797900	-2.649600	-2.984000

C	3.127300	2.833000	-0.987800
H	3.583200	3.796500	-1.153300
C	3.644300	1.583700	-1.298800
H	4.586700	1.292800	-1.740500
C	2.118800	-1.085400	2.777700
H	1.406700	-0.852300	3.557600
C	3.367700	-1.704200	2.854900
H	3.870100	-2.074200	3.734700
C	3.826100	-1.726800	1.545100
H	4.749200	-2.095000	1.120700
B	2.957200	-0.874700	-0.766500
H	4.005900	-1.217100	-1.221800
C	1.890400	2.562200	-0.400800
H	1.156600	3.242800	0.008700
H	-1.259400	-0.853800	1.852200

Table S11: Compound 3A – P Electronic Energy: -1553.758907 Hartree

W	0.050800	-0.591500	-0.201000
O	0.232800	-2.112600	-2.714200
N	0.140400	-1.516500	-1.695600
C	-2.543400	-1.177100	1.519600
H	-2.314700	-0.495200	2.329600
C	-2.316000	-2.679200	-0.263200
H	-1.874300	-3.318200	-1.019400
C	-1.536600	-1.801500	0.634100
H	-0.663900	-2.294800	1.153900
C	-3.775100	-1.545800	1.092100
C	-3.634500	-2.484400	-0.021400
H	-4.721800	-1.205400	1.496200
H	-4.461200	-2.934500	-0.559400
P	2.489800	-1.182600	0.382200
C	3.440500	0.250100	0.981400
H	4.442400	-0.076700	1.273500
H	3.518600	0.999100	0.188700
C	2.582500	-2.424700	1.715300
H	2.112700	-2.040300	2.622300
H	2.066900	-3.334000	1.396500
C	3.467000	-1.916900	-0.966400
H	4.456700	-2.187800	-0.588400
H	2.955500	-2.813300	-1.326700
H	3.632200	-2.654200	1.919900
H	2.930400	0.687700	1.843800
H	3.578100	-1.209800	-1.790200
N	0.070100	0.665900	1.676600
N	-0.133700	1.996700	1.641500
N	0.778700	2.426100	-0.655800
N	0.997600	1.170400	-1.101100
N	-1.636400	2.036200	-0.370800
N	-1.598200	0.743900	-0.764600
C	0.265000	0.330900	2.957200

H	0.434800	-0.702100	3.228900
C	0.188300	1.466900	3.766400
H	0.300000	1.526100	4.837600
C	-0.067000	2.505200	2.882000
H	-0.206300	3.564100	3.046700
C	2.285000	2.572700	-2.276700
H	2.994300	2.961200	-2.990300
C	1.536900	3.287000	-1.348200
H	1.483400	4.346600	-1.142800
C	-2.716400	0.497200	-1.453700
H	-2.896100	-0.490700	-1.854800
C	-3.503400	1.649800	-1.502600
H	-4.461300	1.775600	-1.982400
C	-2.773500	2.601800	-0.806700
H	-2.976000	3.642800	-0.598800
B	-0.410000	2.702000	0.301800
H	-0.580400	3.872100	0.456700
C	1.899300	1.246700	-2.091500
H	2.211800	0.352200	-2.612800

Table S12: Compound 4D Electronic Energy: -1631.116013 Hartree

W	-0.367000	0.164300	-0.017500
O	-2.113700	0.831200	-2.293400
N	-1.397000	0.588200	-1.376700
P	-0.739500	-2.339500	-0.327700
C	0.825000	-3.263000	-0.554100
H	0.605600	-4.334000	-0.561600
H	1.317600	-2.992100	-1.489100
C	-1.519200	-3.328900	0.997600
H	-0.954000	-3.227500	1.927200
H	-2.553300	-3.024000	1.163100
C	-1.766600	-2.729100	-1.783300
H	-1.899600	-3.810800	-1.870200
H	-2.741200	-2.247400	-1.661400
H	-1.506000	-4.377200	0.686800
H	1.492700	-3.038900	0.284300
H	-1.293600	-2.347400	-2.690400
N	1.052100	-0.555400	1.631600
N	2.385900	-0.428900	1.471000
N	2.552800	-0.379700	-1.046900
N	1.252600	-0.395200	-1.403100
N	2.352500	1.742700	0.198600
N	1.026200	1.931100	0.005200
C	0.864500	-1.204100	2.787800
H	-0.132700	-1.418400	3.145900
C	2.092600	-1.504900	3.381300
H	2.268300	-2.017000	4.314300
C	3.034200	-0.988800	2.505200
H	4.113900	-0.973600	2.545200
C	2.483300	-0.939400	-3.191200

H	2.769500	-1.220400	-4.192600
C	3.314400	-0.698900	-2.106700
H	4.391200	-0.714100	-2.017500
C	0.850200	3.229800	-0.259600
H	-0.137600	3.617000	-0.468600
C	2.074600	3.900800	-0.223500
H	2.257800	4.951000	-0.387500
C	3.001100	2.912000	0.065900
H	4.074700	2.953200	0.180500
B	2.975500	0.323200	0.268800
H	4.164400	0.386200	0.350900
C	1.194500	-0.720300	-2.699500
H	0.239500	-0.785200	-3.203200
C	-1.576900	1.041500	1.782900
H	-0.906300	1.065000	2.638300
C	-1.826000	2.248500	1.162900
H	-1.229200	3.077500	1.539000
C	-2.848700	2.632300	0.220100
H	-2.791500	3.660100	-0.125500
C	-3.887900	1.876200	-0.209700
H	-4.611000	2.368200	-0.855600
C	-2.068100	-0.262900	1.341600
H	-1.863100	-1.034200	2.078500
C	-3.432600	-0.393700	0.790700
H	-3.894300	-1.364900	0.963200
C	-4.193200	0.500000	0.120300
H	-5.168800	0.153900	-0.209500

Table S13: Compound 4P Electronic Energy: -1631.113800 Hartree

W	-0.392900	0.041300	-0.046400
O	-2.046600	0.698700	-2.394000
N	-1.404400	0.407100	-1.434700
C	-1.488300	1.166700	1.468500
H	-0.795000	1.377900	2.281600
C	-2.350000	2.313900	1.176000
H	-2.030100	3.243600	1.643700
C	-3.471600	2.389500	0.415700
H	-3.924700	3.370600	0.307800
C	-4.149500	1.318700	-0.262000
H	-4.965600	1.626600	-0.911900
C	-2.052100	-0.187700	1.565100
H	-1.722000	-0.846600	2.362700
C	-3.046100	-0.690400	0.734600
H	-3.255600	-1.752500	0.853900
C	-3.949600	-0.024700	-0.151500
H	-4.591800	-0.677000	-0.734700
P	-0.455200	-2.525800	-0.172200
C	1.210600	-3.236900	-0.435100
H	1.159900	-4.321000	-0.300500
H	1.577500	-3.016500	-1.438800

C	-0.984100	-3.472700	1.302900
H	-0.245400	-3.339400	2.096600
H	-1.964900	-3.168100	1.671000
C	-1.475900	-3.189700	-1.529700
H	-1.386100	-4.278400	-1.578000
H	-2.520700	-2.914700	-1.359800
H	-1.020700	-4.532300	1.033500
H	1.900500	-2.812800	0.301400
H	-1.144900	-2.752000	-2.475100
N	1.145200	-0.370900	1.619900
N	2.437000	-0.012500	1.453700
N	2.607700	-0.058900	-1.058800
N	1.327200	-0.307600	-1.391900
N	2.051300	2.054700	0.080600
N	0.717600	1.984200	-0.111900
C	1.065900	-0.968900	2.815700
H	0.122300	-1.342700	3.186800
C	2.320300	-1.003200	3.428800
H	2.575000	-1.417300	4.391800
C	3.162500	-0.379400	2.522300
H	4.221000	-0.165900	2.561800
C	2.627700	-0.827800	-3.136800
H	2.955000	-1.158900	-4.109900
C	3.411500	-0.358300	-2.092500
H	4.478100	-0.202100	-2.016300
C	0.289200	3.218000	-0.386800
H	-0.758200	3.397500	-0.587600
C	1.363600	4.113500	-0.362800
H	1.341300	5.177200	-0.540700
C	2.464100	3.326400	-0.065400
H	3.509800	3.574700	0.046600
B	2.905900	0.770000	0.214000
H	4.067700	1.032000	0.293600
C	1.319900	-0.764700	-2.648900
H	0.386200	-1.035000	-3.125000

Table S14: Compound TS4a Electronic Energy: -1631.109536 Hartree

W	0.374600	0.175300	-0.013100
O	2.059300	0.881500	2.294000
N	1.385400	0.613500	1.354700
P	0.792900	-2.366500	0.319900
C	1.712000	-2.740100	1.851400
H	1.181300	-2.357300	2.724600
H	1.837800	-3.821800	1.951500
H	2.694300	-2.262400	1.795100
C	-0.810600	-3.238100	0.487700
H	-0.617800	-4.310000	0.585600
H	-1.358300	-2.892300	1.366100
H	-1.413800	-3.062500	-0.408900
C	1.648400	-3.434500	-0.898700

H	1.306200	-3.229700	-1.915300
H	2.728300	-3.284300	-0.837300
H	1.428600	-4.476500	-0.649900
B	-2.990900	0.343500	-0.249100
H	-4.180700	0.402000	-0.320100
N	-2.410700	-0.425000	-1.448200
N	-1.079100	-0.564500	-1.621100
N	-2.545600	-0.350700	1.063500
N	-1.239900	-0.334200	1.397400
N	-2.363300	1.755700	-0.185100
N	-1.038100	1.925100	0.013300
C	-0.912800	-1.339000	-2.702400
H	0.076800	-1.610300	-3.042000
C	-2.150500	-1.699500	-3.238100
H	-2.343600	-2.307000	-4.108200
C	-3.076400	-1.092900	-2.403200
H	-4.156900	-1.086000	-2.421500
C	-1.153400	-0.652600	2.694400
H	-0.190900	-0.682100	3.186200
C	-2.427900	-0.903900	3.206800
H	-2.690600	-1.189300	4.213300
C	-3.282200	-0.686700	2.134900
H	-4.359500	-0.731000	2.062700
C	-0.834500	3.227200	0.227600
H	0.162900	3.599300	0.416100
C	-2.046800	3.920400	0.162800
H	-2.210500	4.978800	0.291400
C	-2.991300	2.941500	-0.098600
H	-4.062900	2.999000	-0.224200
C	1.377400	1.690000	-1.341900
H	0.635100	2.168800	-1.975500
C	2.333500	2.649600	-0.744300
H	2.062200	3.694200	-0.884000
C	3.492500	2.412700	-0.096700
H	4.040000	3.282400	0.256100
C	4.116100	1.131700	0.193900
H	4.988300	1.177300	0.841300
C	3.767200	-0.090700	-0.263700
H	4.359900	-0.942600	0.057900
C	2.720600	-0.408600	-1.223900
H	2.811600	-1.390100	-1.676900
C	1.825700	0.431500	-1.863500
H	1.334500	0.045600	-2.750500

Table S15: Compound TS4b Electronic Energy: -1631.113580 Hartree

W	-0.377300	0.075000	-0.044300
O	-2.157900	0.786900	-2.287900
N	-1.432300	0.477800	-1.388200
C	-1.484900	1.140500	1.521700
H	-0.822600	1.284300	2.373000

C	-2.197900	2.319100	1.153200
H	-1.785600	3.234700	1.576200
C	-3.290700	2.502300	0.329100
H	-3.575900	3.530000	0.128500
C	-4.115900	1.511200	-0.226500
H	-4.937200	1.883800	-0.835200
C	-2.033600	-0.246800	1.431000
H	-1.763100	-0.913500	2.245100
C	-3.232700	-0.601900	0.765900
H	-3.532100	-1.640000	0.907200
C	-4.100800	0.131400	-0.036000
H	-4.900700	-0.429500	-0.507900
P	-0.552200	-2.472000	-0.212900
C	1.077500	-3.271300	-0.455500
H	0.959600	-4.355900	-0.383400
H	1.498600	-3.019800	-1.430200
C	-1.177700	-3.438700	1.210500
H	-1.173200	-4.497100	0.934600
H	-0.515300	-3.297000	2.067600
C	-1.578400	-3.042700	-1.609100
H	-1.587000	-4.134900	-1.660300
H	-2.598800	-2.672600	-1.473000
H	-2.192500	-3.152800	1.490400
H	1.760400	-2.929600	0.329100
H	-1.178500	-2.635600	-2.541100
N	1.131500	-0.427300	1.623900
N	2.440800	-0.142100	1.459300
N	2.611200	-0.169700	-1.057400
N	1.321900	-0.349600	-1.402700
N	2.165100	1.960300	0.109500
N	0.827700	1.974100	-0.077500
C	1.017200	-1.029700	2.813700
H	0.052000	-1.350300	3.180000
C	2.268300	-1.141800	3.425800
H	2.499200	-1.578400	4.384800
C	3.144900	-0.559500	2.524400
H	4.213900	-0.406800	2.565200
C	2.608000	-0.893700	-3.151900
H	2.924900	-1.217900	-4.130700
C	3.406600	-0.485100	-2.093800
H	4.478800	-0.381100	-2.008900
C	0.480000	3.234400	-0.346700
H	-0.552700	3.483400	-0.549800
C	1.608300	4.060600	-0.323500
H	1.652600	5.124400	-0.497000
C	2.657300	3.203800	-0.033400
H	3.716900	3.385200	0.075900
B	2.947600	0.627500	0.227000
H	4.121900	0.827700	0.308600
C	1.301300	-0.778100	-2.668900
H	0.358500	-0.989300	-3.156500

Table S16: Compound 4H – P Electronic Energy: -1631.088290 Hartree

W	0.011600	-0.472600	-0.324500
O	-0.262800	-2.038900	-2.818800
N	-0.149200	-1.378600	-1.822700
C	-5.204900	0.145200	0.840800
H	-6.115200	0.533200	1.288500
C	-5.347400	-0.909500	-0.038600
H	-6.359300	-1.263600	-0.214000
C	-4.332100	-1.580300	-0.736700
H	-4.674700	-2.379300	-1.389800
C	-2.958200	-1.396700	-0.708300
H	-2.415100	-2.097100	-1.339800
C	-4.015900	0.793500	1.205600
H	-4.140700	1.629000	1.890200
C	-2.707200	0.543200	0.820100
H	-1.992100	1.244200	1.246100
C	-2.150100	-0.458100	-0.016900
P	1.577500	-2.216800	0.627500
C	3.072600	-1.502500	1.402400
H	3.667700	-2.303000	1.851100
H	3.674800	-0.976400	0.658400
C	0.931800	-3.310800	1.937500
H	0.616100	-2.715800	2.797300
H	0.072800	-3.865200	1.551700
C	2.198400	-3.406800	-0.608500
H	2.846300	-4.142000	-0.123100
H	1.345300	-3.916100	-1.064900
H	1.711200	-4.011100	2.250300
H	2.768000	-0.797100	2.181500
H	2.760000	-2.890100	-1.388800
N	0.338700	0.583300	1.659400
N	0.959100	1.777800	1.730000
N	2.391600	1.642000	-0.340700
N	1.992900	0.466000	-0.866700
N	0.146100	2.685900	-0.468700
N	-0.515100	1.618300	-0.966400
C	0.053600	0.214400	2.913300
H	-0.467000	-0.715800	3.097700
C	0.502400	1.184800	3.813700
H	0.420500	1.179600	4.889300
C	1.072200	2.163200	3.011900
H	1.544900	3.102600	3.260500
C	4.003100	0.984100	-1.711600
H	4.918800	0.940700	-2.280100
C	3.595400	1.978200	-0.835000
H	4.067500	2.904100	-0.539100
C	-1.464100	2.084700	-1.782700
H	-2.127500	1.396600	-2.289900
C	-1.420800	3.481100	-1.820100
H	-2.051600	4.148500	-2.386100

C	-0.378600	3.817300	-0.969500
H	0.030400	4.777400	-0.689400
B	1.388300	2.517800	0.447000
H	1.865700	3.579800	0.712600
C	2.952100	0.061900	-1.704700
H	2.837200	-0.861700	-2.255300
H	-0.726600	-1.641000	0.658900

Table S17: Compound 4A – D Electronic Energy: -1631.092080 Hartree

W	0.034200	0.121400	0.596400
O	-0.338400	2.577000	2.175200
N	-0.204400	1.538400	1.602400
C	-5.176000	-0.436300	0.494200
H	-6.192300	-0.069700	0.375400
C	-4.861800	-1.602300	-0.110900
H	-5.646200	-2.101300	-0.674000
C	-3.599500	-2.314300	-0.039400
H	-3.631200	-3.331700	-0.423200
C	-2.402900	-1.909400	0.438700
H	-1.614700	-2.660300	0.426100
C	-4.319100	0.378000	1.337600
H	-4.832800	1.179800	1.863000
C	-2.988700	0.299700	1.558100
H	-2.582000	1.031100	2.254600
C	-1.967800	-0.605900	0.985400
P	-1.216100	1.279000	-1.285900
C	-0.202600	1.544900	-2.783300
H	-0.852400	1.905500	-3.585400
H	0.586100	2.276400	-2.602800
C	-2.711300	0.494900	-1.976100
H	-2.484500	-0.486800	-2.393800
H	-3.474700	0.399000	-1.204600
C	-1.834600	2.920300	-0.791300
H	-2.345400	3.388300	-1.637200
H	-2.541300	2.790100	0.033900
H	-3.086300	1.142600	-2.774000
H	0.249200	0.594200	-3.083400
H	-1.016400	3.561600	-0.459500
N	0.511600	-1.420000	-1.070200
N	1.793300	-1.598500	-1.461300
N	2.752400	0.649400	-0.932400
N	1.670500	1.241800	-0.387200
N	2.897400	-1.164900	0.755700
N	1.848600	-0.765800	1.506600
C	-0.244600	-2.130000	-1.918700
H	-1.317500	-2.160200	-1.801900
C	0.553100	-2.770200	-2.868900
H	0.235100	-3.413700	-3.674000
C	1.846400	-2.400400	-2.536400
H	2.797900	-2.649600	-2.984000

C	3.127300	2.833000	-0.987800
H	3.583200	3.796500	-1.153300
C	3.644300	1.583700	-1.298800
H	4.586700	1.292800	-1.740500
C	2.118800	-1.085400	2.777700
H	1.406700	-0.852300	3.557600
C	3.367700	-1.704200	2.854900
H	3.870100	-2.074200	3.734700
C	3.826100	-1.726800	1.545100
H	4.749200	-2.095000	1.120700
B	2.957200	-0.874700	-0.766500
H	4.005900	-1.217100	-1.221800
C	1.890400	2.562200	-0.400800
H	1.156600	3.242800	0.008700
H	-1.259400	-0.853800	1.852200

Table S18: Compound 4A – P Electronic Energy: -1631.095218 Hartree

W	0.137600	-0.575000	-0.144800
O	0.043700	-2.139100	-2.637900
N	0.041000	-1.544700	-1.606900
C	-4.714700	-0.249800	0.740400
H	-5.572300	0.405800	0.870000
C	-4.784700	-1.181300	-0.234900
H	-5.693600	-1.217400	-0.830100
C	-3.790400	-2.189900	-0.553900
H	-4.137300	-2.980300	-1.215600
C	-2.497700	-2.270400	-0.170500
H	-1.940800	-3.129900	-0.539700
C	-3.643300	-0.071500	1.703900
H	-3.903400	0.544900	2.561700
C	-2.383800	-0.558500	1.692300
H	-1.771400	-0.309300	2.555100
C	-1.688400	-1.333000	0.640400
P	2.410400	-1.635200	0.501500
C	3.668100	-0.369800	0.882500
H	4.596700	-0.862000	1.185500
H	3.855000	0.247000	-0.000500
C	2.384900	-2.714100	1.973700
H	2.078500	-2.140600	2.851000
H	1.675900	-3.529800	1.811500
C	3.155000	-2.688600	-0.785500
H	4.105100	-3.092200	-0.424400
H	2.471200	-3.510800	-1.011500
H	3.383600	-3.127000	2.142500
H	3.308300	0.265900	1.696300
H	3.331500	-2.110200	-1.694500
N	0.497500	0.837800	1.611200
N	0.588500	2.171200	1.450000
N	1.457100	2.188000	-0.899300
N	1.386000	0.878700	-1.213400

N	-0.971100	2.362600	-0.509500
N	-1.238700	1.062800	-0.762000
C	0.720100	0.582900	2.906200
H	0.687400	-0.434600	3.273200
C	0.958800	1.773900	3.596100
H	1.166200	1.901500	4.647000
C	0.865800	2.760000	2.624100
H	0.976800	3.833100	2.684300
C	2.907200	1.844100	-2.540300
H	3.661000	1.995200	-3.296900
C	2.361900	2.792000	-1.683600
H	2.546600	3.852300	-1.586400
C	-2.410100	1.011200	-1.397900
H	-2.819000	0.056000	-1.696600
C	-2.923500	2.301900	-1.556900
H	-3.849900	2.591700	-2.027400
C	-1.970900	3.127500	-0.980400
H	-1.927000	4.202200	-0.877000
B	0.401100	2.799300	0.056900
H	0.495300	3.987900	0.104100
C	2.249200	0.658000	-2.214200
H	2.343700	-0.330800	-2.642100
H	-0.861900	-1.953900	1.132800

Crystallography.

A single crystal of each of the molecules listed in the tables below was coated with Paratone oil and mounted on a MiTeGen micromount. Data were collected on a Bruker D8 VENTURE dual wavelength Mo/Cu Kappa four-circle diffractometer using a PHOTON III detector. The diffractometer was equipped with an Oxford Cryostream 800Plus low temperature device and used Cu K_{α} radiation (λ = 1.54178Å) from an Incoatec Iμs 3.0 microfocus sealed X-ray tube with a HELIOS EF double bounce multilayer mirror as monochromator for **3[DDQ]**, or Mo K_{α} radiation (λ = 0.71073Å) from an Incoatec IμS 3.0 microfocus sealed X-ray tube with a HELIOS double bounce multilayer mirror as monochromator (all other crystals).

Data collection and processing were done within the Bruker APEX5 or APEX6 software suite.[1] All data were integrated with SAINT 8.40B using a narrow-frame algorithm and a Multi-Scan absorption correction using SADABS was applied.[2] Using Olex2 as a graphical interface,[3] each structure was solved by dual methods with SHELXT[4] and refined by full-matrix least-squares methods against F^2 using SHELXL.[5] All non-hydrogen atoms were refined with anisotropic displacement parameters. In all structures except **3[DDQ], 7**, **8** the B-H hydrogen atoms were located in the electron density map and refined isotropically. In all structures except **3[DDQ], 6, 8, 10** the hydrogen atoms on carbons directly bound to W were also located in the electron density map and refined isotropically. In **3[DDQ]** and **8**, all hydrogen atoms were placed in calculated positions, while in **3[OTf]** and **6**, all the hydrogen atoms bound to carbon in the coordinated 5-membered ring were found. All other hydrogen atoms in all structures were placed in calculated positions using a riding model with their U_{iso} values constrained to 1.5 times the U_{eq} of their pivot atoms for terminal sp^3 carbon atoms and 1.2 times for all other carbon atoms. Most CIF files were generated using FinalCif.[6]

For **3**, the relative occupancy of the disordered atoms was freely refined. Constraints and restraints were used as needed on the anisotropic displacement parameters and/or bond lengths of the disordered atoms. In **6**, one solvent molecule was refined at 50% occupancy to account for the symmetry of the site. For **7a** and **12**, the structures were refined as a two-domain twins on HLKF 5 data, with the BASF refining to 0.397 for **7a** and 0.4807 for **12**.

	**2**	**3[DDQ]**	**3[OTf]**	**4**	**6**
CCDC number	2432184	2432185	2432811	2432186	2432187
Empirical formula	$C_{20}H_{27}BN_7OPW$	$C_{26}H_{26}BCl_4N_9O_3PW$	$C_{18}H_{24}BF_3N_7O_4PSW$	$C_{20}H_{26}BF_3N_7O_4PSW$	$C_{19}H_{28}BN_8OPW$
Formula weight	607.11	879.99	717.13	743.17	610.12
Temp [K]	100(2)	100(2)	100(2)	100(2)	100(2)
Cryst. system	monoclinic	monoclinic	triclinic	monoclinic	monoclinic
Space group	P2$_1$/c	P2$_1$/c	P -1	P2$_1$/n	I2/a
a [Å]	12.2214(5)	9.5771(3)	7.7218(3)	14.6316(6)	15.6936(6)
b [Å]	10.1317(4)	25.8938(9)	12.2636(5)	10.9406(3)	12.3105(5)
c [Å]	19.2532(6)	13.4294(3)	13.5389(6)	18.1297(7)	25.7530(14)
α [°]	90	90	85.6380(10)	90	90
β [°]	105.3930(10)	107.546(2)	76.4100(10)	113.3850(10)	92.2240(10)
γ [°]	90	90	84.2940(10)	90	90
Volume [Å^3]	2298.48(15)	3175.38(17)	1238.18(9)	2663.79(17)	4971.6(4)
Z	4	4	2	4	8
ρ_{calc} [gcm^{-3}]	1.754	1.841	1.924	1.853	1.630
μ [mm^{-1}]	5.122	10.708	4.877	4.537	4.738
F(000)	1192	1724	700	1456	2400
Crystal size [mm^3]	0.124×0.076×0.048	0.127×0.027×0.018	0.04×0.052×0.094	0.478×0.235×0.128	0.029×0.084×0.089
Crystal colour	red	yellow	yellow	orange	yellow

Crystal shape	plate	needle	needle	needle	plate
λ [Å]	0.71073	1.54178	0.71073	0.71073	0.71073
2Θ range [°]	4.58 to 55.08	7.70 to 133.22	4.43 to 59.17	4.46 to 59.18	4.21 to 52.78
Index ranges	$-15 \le h \le 15$<br>$-13 \le k \le 13$<br>$-25 \le l \le 23$	$-11 \le h \le 11$<br>$-29 \le k \le 30$<br>$-15 \le l \le 15$	$-10 \le h \le 9$<br>$-17 \le k \le 16$<br>$-18 \le l \le 18$	$-20 \le h \le 20$<br>$-15 \le k \le 11$<br>$-25 \le l \le 25$	$-19 \le h \le 18$<br>$-15 \le k \le 15$<br>$-32 \le l \le 32$
Reflections collected	67248	25375	42140	47564	47812
Independent reflections	5292 [R_{int} = 0.0595]	5606 [R_{int} = 0.1649]	6924 [R_{int} = 0.0585]	7467 [R_{int} = 0.0453]	5081 [R_{int} = 0.0791]
Completeness	99.9 %	99.9 %	100.0	99.9 %	100.0
Data / Restraints / Parameters	5292/0/295	5606/20/425	6924 / 0 / 351	7467/0/358	5081 / 1 / 340
Goodness-of-fit on F^2	1.099	1.046	1.005	1.051	1.034
Final *R* indexes [$I \geq 2\sigma(I)$]	R_1 = 0.0231<br>wR_2 = 0.0482	R_1 = 0.0624<br>wR_2 = 0.1326	R_1 = 0.0275<br>wR_2 = 0.0572	R_1 = 0.0213<br>wR_2 = 0.0505	R_1 = 0.0310<br>wR_2 = 0.0705
Final *R* indexes [all data]	R_1 = 0.0278<br>wR_2 = 0.0498	R_1 = 0.1120<br>wR_2 = 0.1550	R_1 = 0.0368<br>wR_2 = 0.0602	R_1 = 0.0243<br>wR_2 = 0.0520	R_1 = 0.0485<br>wR_2 = 0.0774
Largest peak/hole [eÅ^{-3}]	0.86/-1.50	1.28/-0.76	1.29/−0.65	0.94/-1.03	1.12/−0.71

	**7A**	**7**	**8**	**10**	**12**	**13**
CCDC number	2432188	2432189	2432190	2432191	2441187	2432192
Empirical formula	$C_{20}H_{28}BF_3N_7O_4PSW$	$C_{20}H_{28}BCl_3N_7OPW$	$C_{21}H_{28}BF_3N_7O_4PSW$	$C_{22}H_{32}BCl_3N_7OPW$	$C_{20}H_{29}BN_7OPW$	$C_{30}H_{40}BN_8OPW$
Formula weight	745.18	714.47	757.19	742.52	609.13	754.33
Temp [K]	100(2)	100.00	100(2)	100(2)	100(2)	100(2)
Cryst. system	triclinic	monoclinic	triclinic	monoclinic	monoclinic	monoclinic
Space group	P -1	$P2_1/n$	P -1	$P2_1/c$	$P2_1/c$	$P2_1/c$
a [Å]	10.0480(5)	10.3682(7)	9.5771(5)	11.6338(9)	9.6307(6)	16.8919(6)
b [Å]	10.4236(6)	12.4816(9)	10.7462(5)	14.6139(10)	16.9167(11)	18.6996(6)
c [Å]	12.7780(8)	20.3456(17)	13.8016(7)	16.7964(12)	14.5107(8)	10.1419(4)
α [°]	85.658(2)	90	93.960(2)	90	90	90
β [°]	86.388(2)	90.196(3)	109.386(2)	92.492(2)	104.832(2)	97.0400(10)
γ [°]	82.542(2)	90	91.001(2)	90	90	90
Volume [Å^3]	1321.28(13)	2632.9(3)	1335.46(12)	2852.9(4)	2285.3(2)	3179.4(2)
Z	2	4	2	4	4	4
ρ_{calc} [gcm^{-3}]	1.873	1.802	1.883	1.729	1.770	1.576
μ [mm^{-1}]	4.574	4.781	4.527	4.416	5.152	3.721
F(000)	732	1400	744	1464	1200	1512
Crystal size [mm^3]	0.096×0.008×0.006	0.066×0.12×0.125	0.089×0.069×0.050	0.072×0.054×0.043	0.044×0.086×0.122	0.059×0.081×0.151
Crystal colour	yellow	yellow	yellow	yellow	yellow	colourless

Crystal shape	block	needle	plate	block	needle	needle
λ [Å]	0.71073	0.71073	0.71073	0.71073	0.71073	0.71073
2Θ range [°]	4.09 to 50.07	5.11 to 55.00	3.80 to 56.56	3.50 to 46.52	3.77 to 52.79	4.60 to 61.19
Index ranges	-11 ≤ h ≤ 11<br>-12 ≤ k ≤ 12<br>0 ≤ l ≤ 15	−13 ≤ h ≤ 13<br>−16 ≤ k ≤ 13<br>−23 ≤ l ≤ 26	-12 ≤ h ≤ 12<br>-14 ≤ k ≤ 14<br>-18 ≤ l ≤ 14	-12 ≤ h ≤ 12<br>-16 ≤ k ≤ 16<br>-17 ≤ l ≤ 18	−12 ≤ h ≤ 11<br>0 ≤ k ≤ 21<br>0 ≤ l ≤ 18	−24 ≤ h ≤ 24<br>−20 ≤ k ≤ 26<br>−14 ≤ l ≤ 14
Reflections collected	4657	34496	36468	21647	4669	71130
Independent reflections	4657 [R_{int} = 0.1535]	6026 [R_{int} = 0.1138]	6614 [R_{int} = 0.0378]	4096 [R_{int} = 0.1635]	4669 [R_{int} = 0.0905]	9765 [R_{int} = 0.0909]
Completeness	99.8 %	99.9	99.7 %	99.9 %	100.0	99.9%
Data / Restraints / Parameters	4657/3/360	6026 / 0 / 317	6614/0/355	4096/0/332	4669 / 0 / 297	9765 / 0 / 400
Goodness-of-fit on F^2	1.083	1.019	1.046	1.003	1.042	1.005
Final R indexes [$I \geq 2\sigma(I)$]	R_1 = 0.0548<br>wR_2 = 0.1230	R_1 = 0.0441<br>wR_2 = 0.0874	R_1 = 0.0223<br>wR_2 = 0.0511	R_1 = 0.0608<br>wR_2 = 0.1309	R_1 = 0.0306<br>wR_2 = 0.0627	R_1 = 0.0367<br>wR_2 = 0.0724
Final R indexes [all data]	R_1 = 0.0741<br>wR_2 = 0.1331	R_1 = 0.0784<br>wR_2 = 0.0998	R_1 = 0.0259<br>wR_2 = 0.0528	R_1 = 0.1265<br>wR_2 = 0.1587	R_1 = 0.0481<br>wR_2 = 0.0691	R_1 = 0.0627<br>wR_2 = 0.0813
Largest peak/hole [eÅ^{-3}]	3.03/-0.80	1.41/−1.50	1.40/-0.53	1.60/-1.57	1.56/−0.71	1.14/−1.24

1. Bruker (2019). *Saint; APEX5*. Bruker AXS Inc., Madison, Wisconsin, USA
2. Krause, L.; Herbst-Irmer, R; Sheldrick, G. M.; Stalke, D., Comparison of silver and molybdenum microfocus X-ray sources for single-crystal structure determination. *J. Appl. Cryst.* **2015**, *48*, 3-10. doi:10.1107/S1600576714022985
3. Dolomanov, O. V.; Bourhis, L. J.; Gildea, R. J.; Howard, J. A. K.; Puschmann, H., OLEX2: a complete structure solution, refinement and analysis program. *J. Appl. Cryst.* **2009**, ***42***, 339-341. doi:10.1107/S0021889808042726
4. Sheldrick, G. M., SHELXT – Integrated space-group and crystal structure determination. *Acta Crystallogr. Sect. A: Found. Adv.* **2015**, *A71*, 3-8. doi:10.1107/S2053273314026370
5. Sheldrick, G. M., Crystal structure refinement with SHELXL. *Acta Crystallogr. Sect C: Struct. Chem.* **2015**, *C71*, 3-8. doi: 10.1107/S2053229614024218
6. Kratzert, D. FinalCif, https://dkratzert.de/finalcif.html.
